# Supplementary material for: Molecular mechanism of decision-making in glycosaminoglycan biosynthesis
Source: Nat Commun. 2023 Oct 13;14:6425. doi: 10.1038/s41467-023-42236-z (PMC10570366; doi:10.1038/s41467-023-42236-z)
Supplement: Supplementary file 1 — Supplementary Information [file 41467_2023_42236_MOESM1_ESM.pdf]

Supplementary Information

Molecular mechanism of decision-making in glycosaminoglycan biosynthesis

Douglas Sammon[1], Anja Krueger[1], Marta Busse-Wicher[1†], Rhodri Marc Morgan[1‡], Stuart M. Haslam[1], Benjamin Schumann[2,3], David C. Briggs[1,4]* and Erhard Hohenester[1]*

[1]Department of Life Sciences, Imperial College London, London SW7 2AZ, UK

[2]Department of Chemistry, Imperial College London, London W12 0BZ, UK

[3]Chemical Glycobiology Laboratory, The Francis Crick Institute, London NW1 1AT, UK

[4]Signalling and Structural Biology Laboratory, The Francis Crick Institute, London NW1 1AT, UK

[†]Present address: Abzena, Babraham Research Campus, Cambridge, CB22 3AT, UK

[‡]Present address: ZoBio, 2333 CH Leiden, Netherlands

*Corresponding authors: e.hohenester@imperial.ac.uk, david.briggs@crick.ac.uk

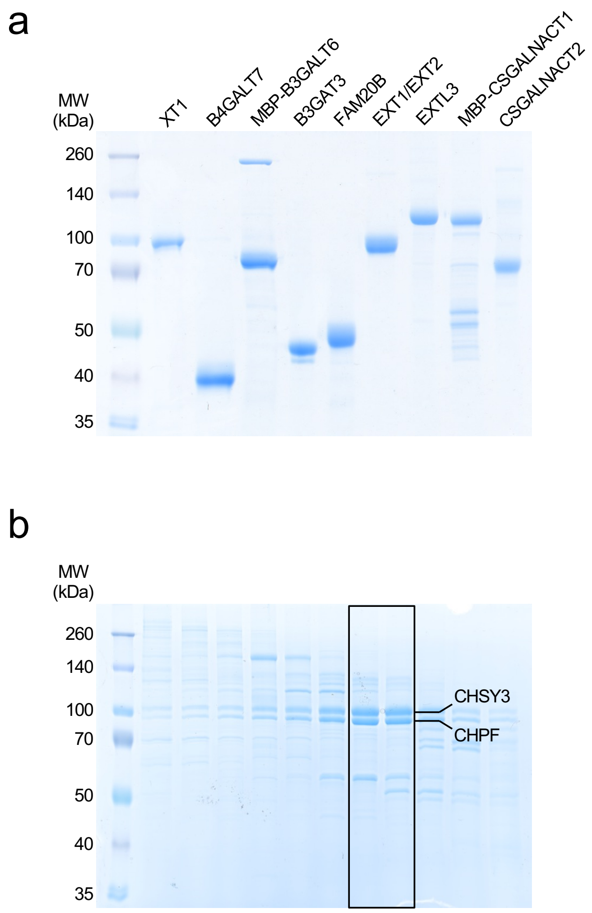

Supplementary Fig. 1 | Purified enzymes used in this study.

a Soluble enzymes were purified from the culture medium of transiently transfected Expi293 cells. Protein samples were boiled, separated by SDS-PAGE, and stained with Coomassie Brilliant Blue.

b CHSY3 and CHPF were co-expressed in Expi293 cells. The CHSY3/CHPF complex was purified by HisTrap affinity chromatography followed by size exclusion chromatography. Aliquots of fractions were boiled and analysed by SDS-PAGE and Coomassie staining. The black box indicates the fractions pooled for biochemical analysis. Molecular weight markers are labelled in a and b.

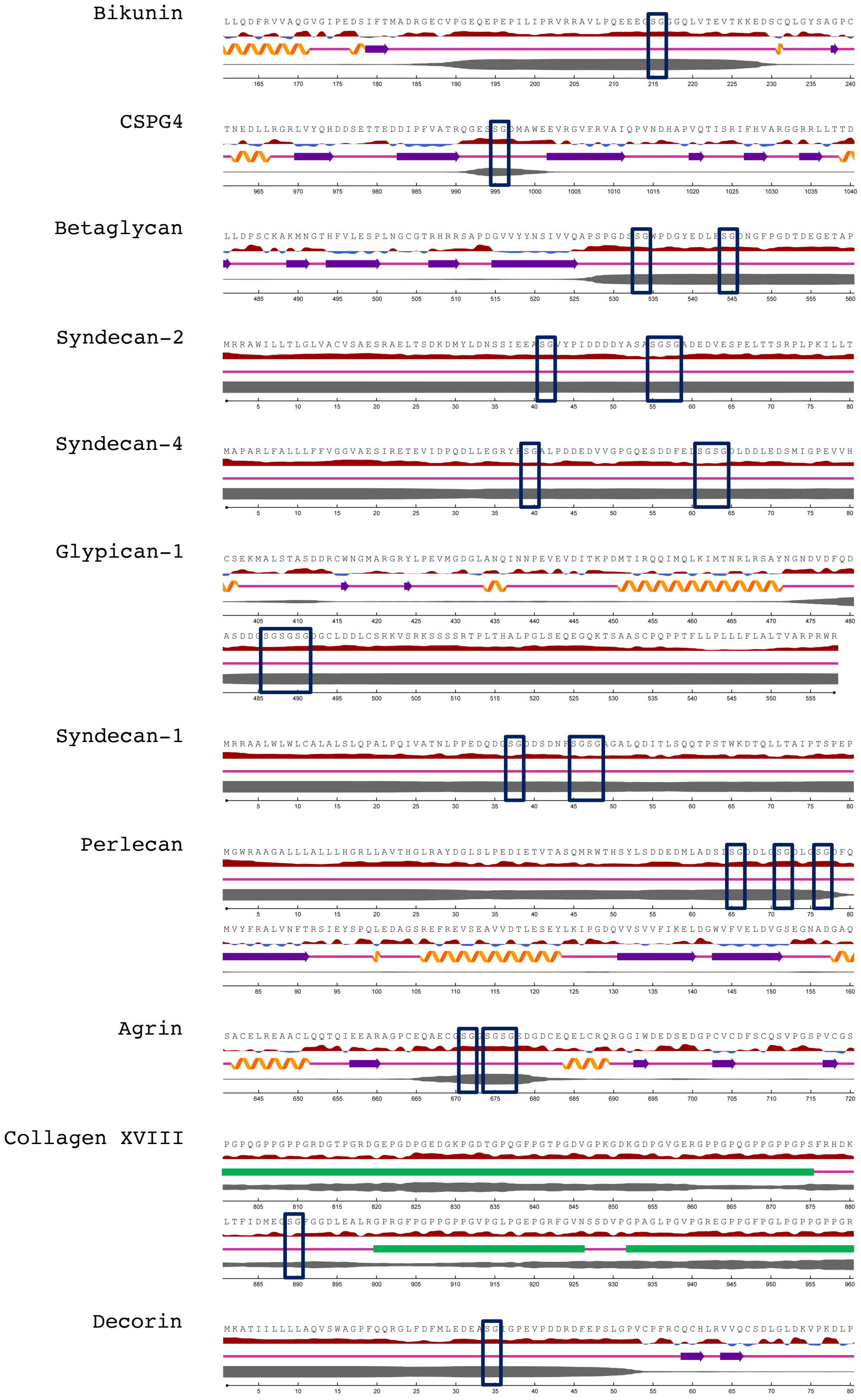

Supplementary Fig. 2 | Location of GAG attachment sites in unstructured protein regions. NetSurfP 3.0[1] was used to predict the structural context of the Ser-Gly sequons analysed in this study (first six proteins) and of five other important proteoglycans. All proteins are human, except for betaglycan which is murine. Solvent accessibility and disorder are shown in red and grey, respectively, with the width of the grey ribbon indicating the probability of disorder. The Ser-Gly sequons are boxed. The triple helix-forming Gly-X-Y repeats of collagen XVIII are indicated by horizontal green bars.

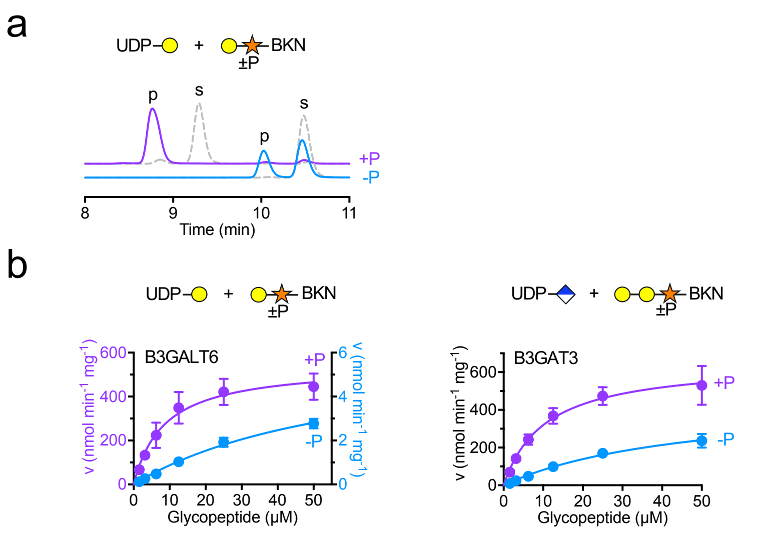

Acceptor	B3GALT6			B3GAT3		
	K_M (μM)	k_{cat} (min^{-1})	k_{cat}/K_M ($μM^{-1}min^{-1}$)	K_M (μM)	k_{cat} (min^{-1})	k_{cat}/K_M ($μM^{-1}min^{-1}$)
Gal-Xyl-BKN	63±11[a]	0.48±0.06	0.0076			
Gal-Xyl2P-BKN	8.5±1.7	41±3	4.8			
Gal-Gal-Xyl-BKN				47±10	17±2	0.36
Gal-Gal-Xyl2P-BKN				11±2	24±2	2.3

[a]K_M exceeds the highest substrate concentration tested and is therefore not well determined by the data.

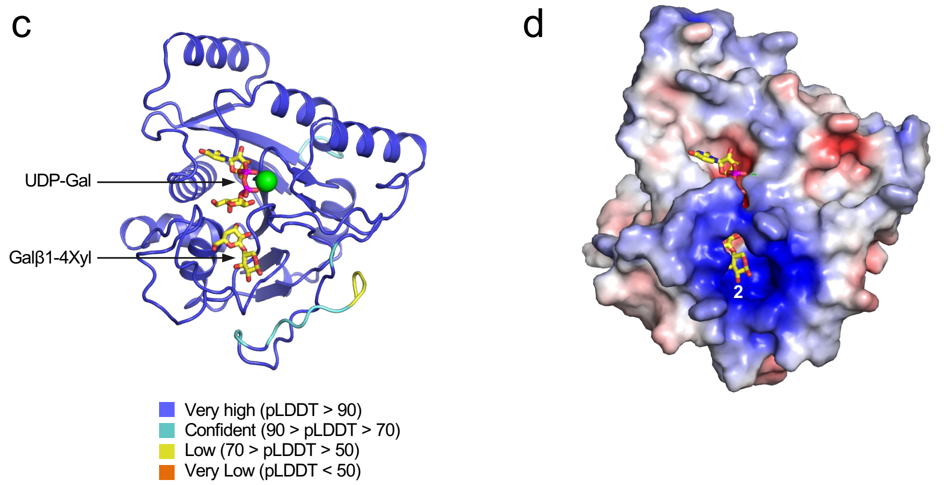

Supplementary Fig. 3 | Xyl phosphorylation enhances B3GALT6 and B3GAT3 activity.
a High-performance liquid chromatography analysis of the reaction catalysed by MBP-B3GALT6, using Gal-Xyl(2P)-BKN as acceptors. Substrate (s) and product (p) peaks are labelled. The identity of the products was verified by mass spectrometry (Supplementary Table 2). The dashed grey lines represent the acceptor glycopeptides alone. **b** Kinetic analysis of the reactions catalysed by MBP-B3GALT6 and B3GAT3, using the indicated glycopeptides as acceptors. Initial rates were determined using the UDP-Glo assay, over a range of glycopeptide concentrations in the presence of constant 100 μM UDP-sugar (UDP-Gal for MBP-B3GALT6, UDP-GlcA for B3GAT3). Data points are shown as mean ± SEM (standard error of the mean) from n=3 independent experiments and were fitted with the Michaelis-Menten equation. The kinetic parameters and their standard deviations from the non-linear fit are given in the table below the graphs. **c** AlphaFold[2] prediction of the B3GALT6 structure with UDP-Gal and Galβ1-4Xyl modelled as described in Methods. The cartoon is coloured by the per-residue confidence score, pLDDT. A Mn^{2+} ion is shown as a green sphere. **d** Electrostatic surface presentation of the predicted B3GALT6 structure (blue, positive potential; red, negative potential). The 2-OH group of Xyl, which becomes phosphorylated by FAM20B, is labelled.

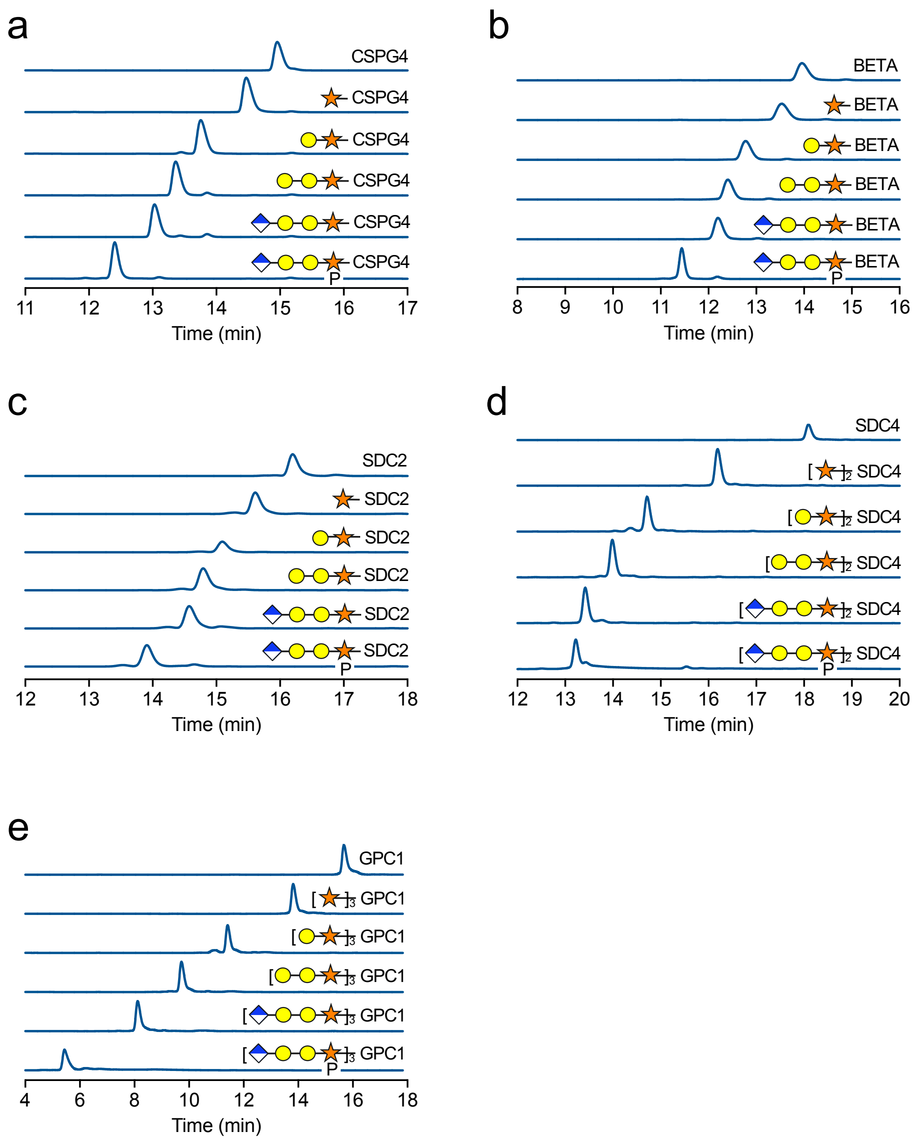

Supplementary Fig. 4 | One-pot multienzyme synthesis of GAG tetrasaccharide linkers.
a-e High-performance liquid chromatography analysis of one-pot multienzyme reaction products obtained with the indicated peptides. SDC4 and GPC1 contain 2 and 3 Ser-Gly sequons, respectively. All Tetra and TetraP products were verified by mass spectrometry (Supplementary Table 2).

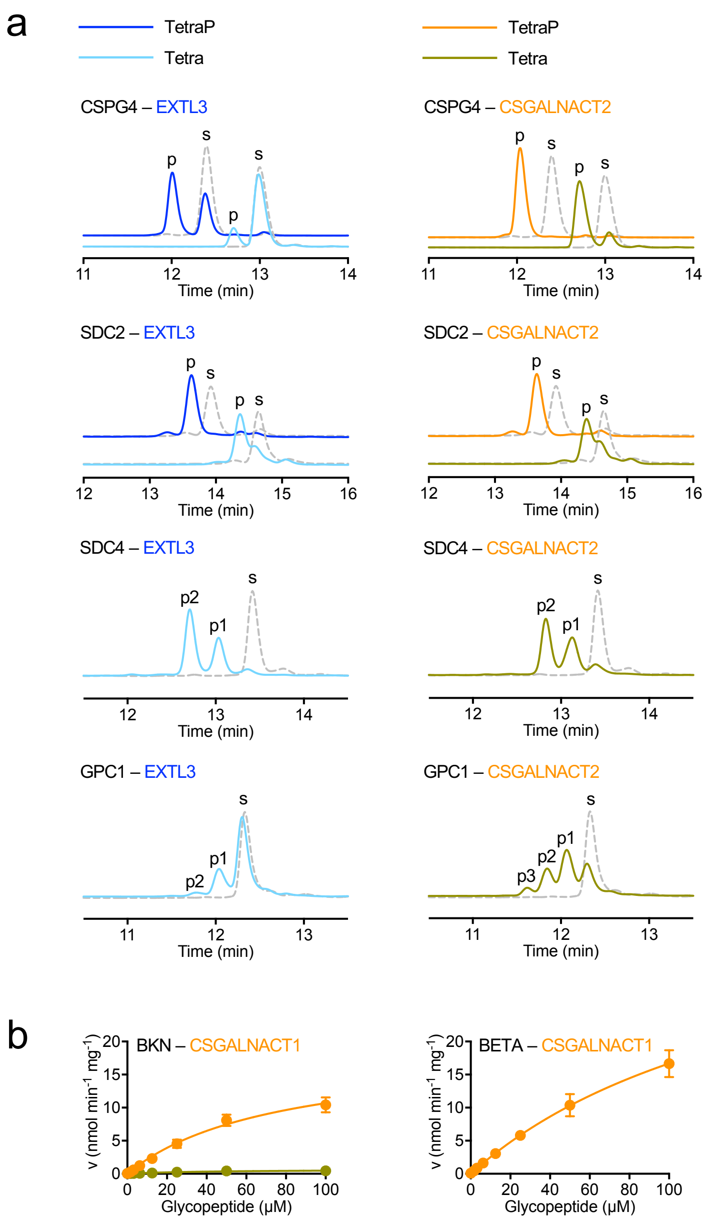

Acceptor	CSGALNACT1		
	K_M (µM)	k_{cat} (min^{-1})	k_{cat}/K_M ($\mu M^{-1} min^{-1}$)
Tetra-BKN	45±22	0.068±0.015	0.0015
TetraP-BKN	76±13[a]	1.9±0.2	0.025
TetraP-BETA	160±40[a]	4.4±0.7	0.027

[a]K_M exceeds the highest substrate concentration tested and is therefore not well determined by the data.

Supplementary Fig. 5 | HS but not CS initiation is peptide sequence-dependent.
a High-performance liquid chromatography analysis of the priming reactions catalysed by EXTL3 and CSGALNACT2, using the indicated acceptor glycopeptides. Substrate (s) and product (p) peaks are labelled. For SDC4 and GPC1, which contain 2 and 3 Ser-Gly sequons, respectively, multiple products were obtained (p1-p3, verified by mass spectrometry; Supplementary Table 2). The dashed grey lines represent the acceptor glycopeptides alone. **b** Kinetic analysis of priming reactions catalysed by MBP-CSGALNACT1, using the indicated glycopeptides as acceptors. The colour code is the same as in a: orange for TetraP-peptides, olive for Tetra-peptides. Initial rates were determined using the UDP-Glo assay, over a range of glycopeptide concentrations in the presence of constant 100 µM UDP-GalNAc. Data points are shown as mean ± SEM (standard error of the mean) from n=3 independent experiments and were fitted with the Michaelis-Menten equation. The kinetic parameters and their standard deviations from the non-linear fit are given in the table below the graphs.

SDC4-A ESDDFELAGSGDLDDLEDW

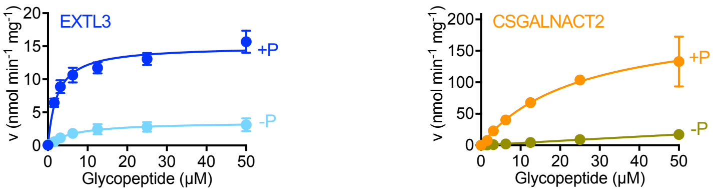

Acceptor	EXTL3			CSGALNACT2		
	K_M (μM)	k_{cat} (min^{-1})	k_{cat}/K_M ($μM^{-1}min^{-1}$)	K_M (μM)	k_{cat} (min^{-1})	k_{cat}/K_M ($μM^{-1}min^{-1}$)
Tetra-SDC4-A	6.4±2.1	0.36±0.04	0.056	NF	NF	
TetraP-SDC4-A	2.3±0.4	1.5±0.1	0.65	24.5±7.1	12.5±1.7	0.51

Supplementary Fig. 6 | Priming of syndecan-4 glycopeptides with a single Ser-Gly sequon. Tetra and TetraP linkers were assembled on the SDC4-A peptide using one-pot multienzyme synthesis and verified by mass spectrometry (Supplementary Table 2). Initial rates were determined using the UDP-Glo assay, over a range of glycopeptide concentrations in the presence of constant 100 µM UDP-sugar (UDP-GlcNAc for EXTL3, UDP-GalNAc for CSGALNACT2). Data points are shown as mean ± SEM (standard error of the mean) from n=3 independent experiments and were fitted with the Michaelis-Menten equation. The kinetic parameters and their standard deviations from the non-linear fit are given in the table below the graphs. NF, no fit obtained.

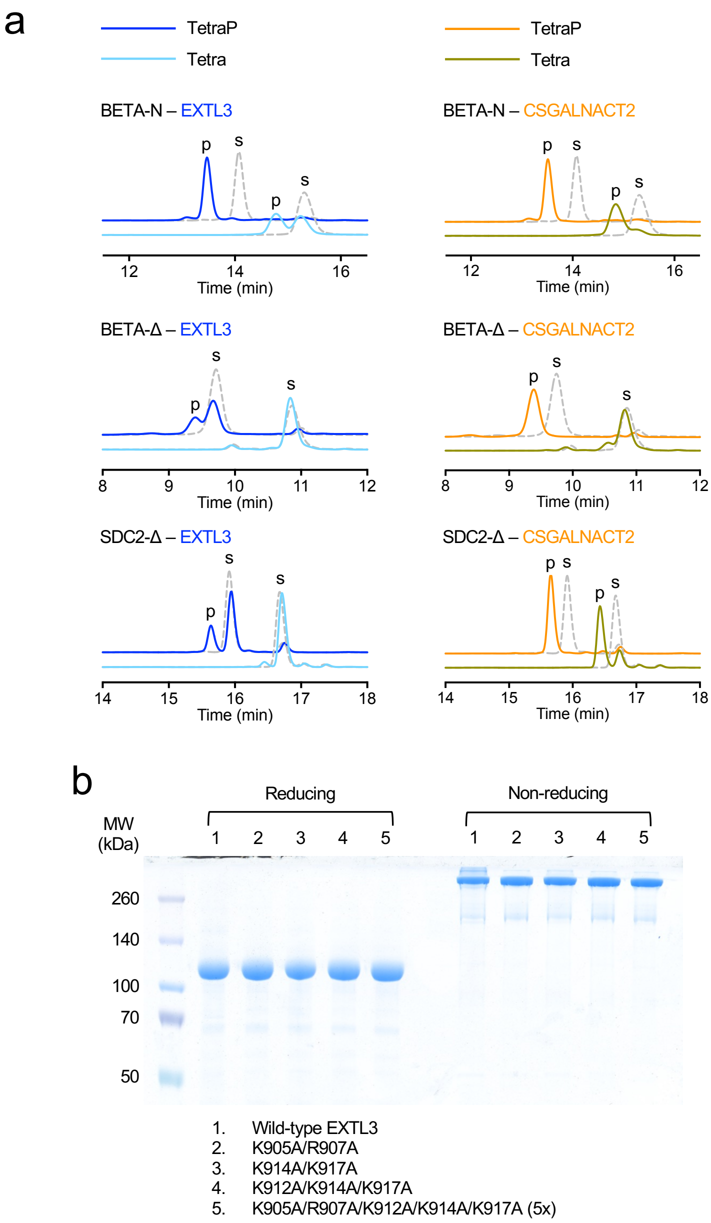

Supplementary Fig. 7 | EXTL3 peptide specificity is governed by a basic exosite.
a High-performance liquid chromatography analysis of the priming reactions catalysed by EXTL3 and CSGALNACT2, using the indicated acceptor glycopeptides. Substrate (s) and product (p) peaks are labelled. The identity of the products was verified by mass spectrometry (Supplementary Table 2). The dashed grey lines represent the acceptor glycopeptides alone. **b** Reducing and non-reducing SDS-PAGE analysis of wild-type and mutant EXTL3. The indicated protein samples were boiled, separated by SDS-PAGE, and stained with Coomassie Brilliant Blue.

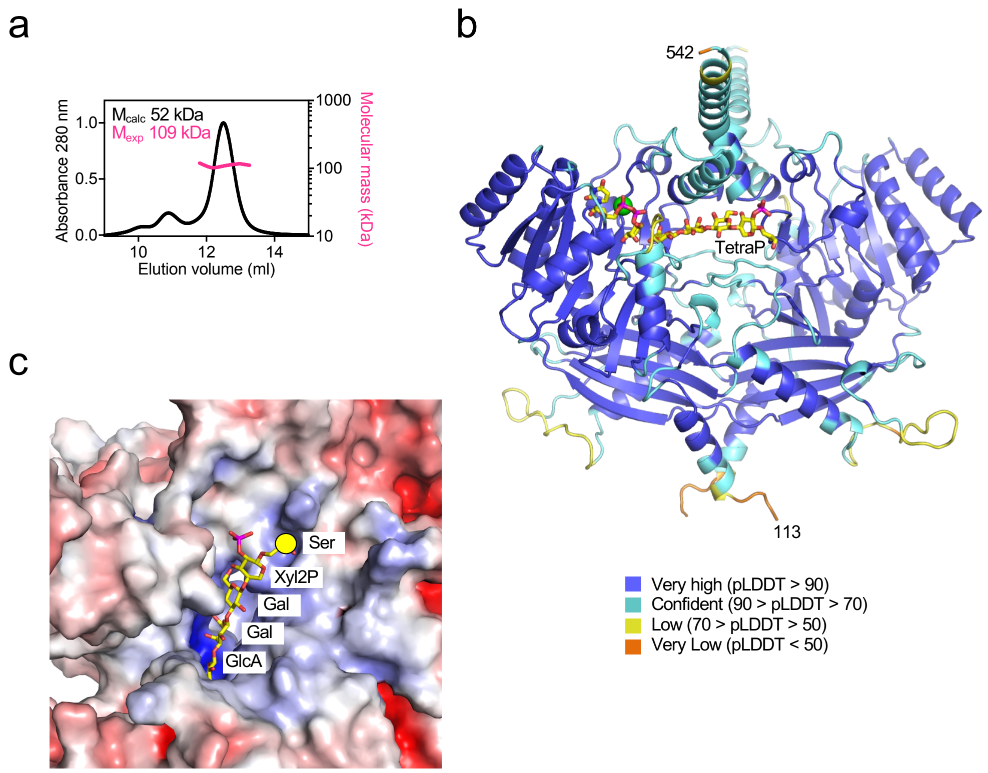

Supplementary Fig. 8 | Predicted CSGALNACT2 structure.
a Size exclusion chromatography with multi-angle light scattering analysis of CSGALNACT2 lacking the N-terminal coiled coil (ΔCC). Calculated and experimental molecular masses are indicated. **b** ColabFold[3] prediction of the dimeric CSGALNACT2 ΔCC structure. The cartoon is coloured by the per-residue confidence score, pLDDT. Substrates were modelled as described in Methods. A Mn^{2+} ion is shown as a green sphere. **c** Electrostatic surface representation of the predicted CSGALNACT2 structure in the vicinity of the GalNAc transferase site (blue, positive potential; red, negative potential). The phosphorylated tetrasaccharide linker is shown in stick representation. The yellow circle indicates the Cα atom of the modified serine.

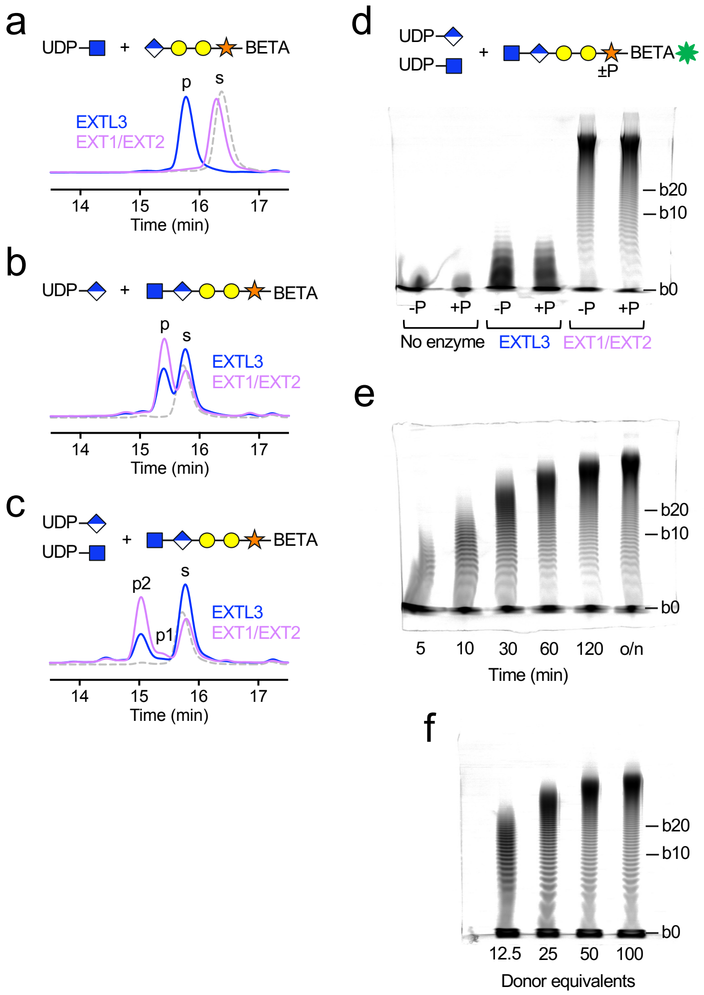

Supplementary Fig. 9 | Xyl phosphorylation does not affect HS backbone polymerisation.
a-c High-performance liquid chromatography analysis of priming and elongation reactions catalysed by EXTL3 and EXT1/EXT2, using the indicated donors and acceptors. Substrate (s) and product (p) peaks are labelled. p1 is GlcA-GlcNAc-Tetra-BETA and p2 is GlcNAc-GlcA-GlcNAc-Tetra-BETA (verified by mass spectrometry; Supplementary Table 2). The dashed grey lines represent the acceptor glycopeptides alone. **d** GlcNAc-Tetra(P)-BETA-5-FAM was incubated overnight with EXTL3 or EXT1/EXT2 and 100 equivalents each of UDP-GlcA and UDP-GlcNAc. Reactions were stopped by boiling, separated by SDS-PAGE, and detected by in-gel fluorescence. The fastest migrating band (b0) corresponds to the unmodified glycopeptide. Each sugar addition results in an additional, slower migrating, band; the 10th and 20th band are labelled. **e** Time course of polymerisation catalysed by EXT1/EXT2 using GlcNAc-Tetra-BETA as the initial acceptor (o/n, overnight). **f** Overnight reactions catalysed by EXT1/EXT2 using GlcNAc-TetraP-BETA as the initial acceptor and varying amounts of donors, as indicated. Representative gels from three independent experiments are shown.

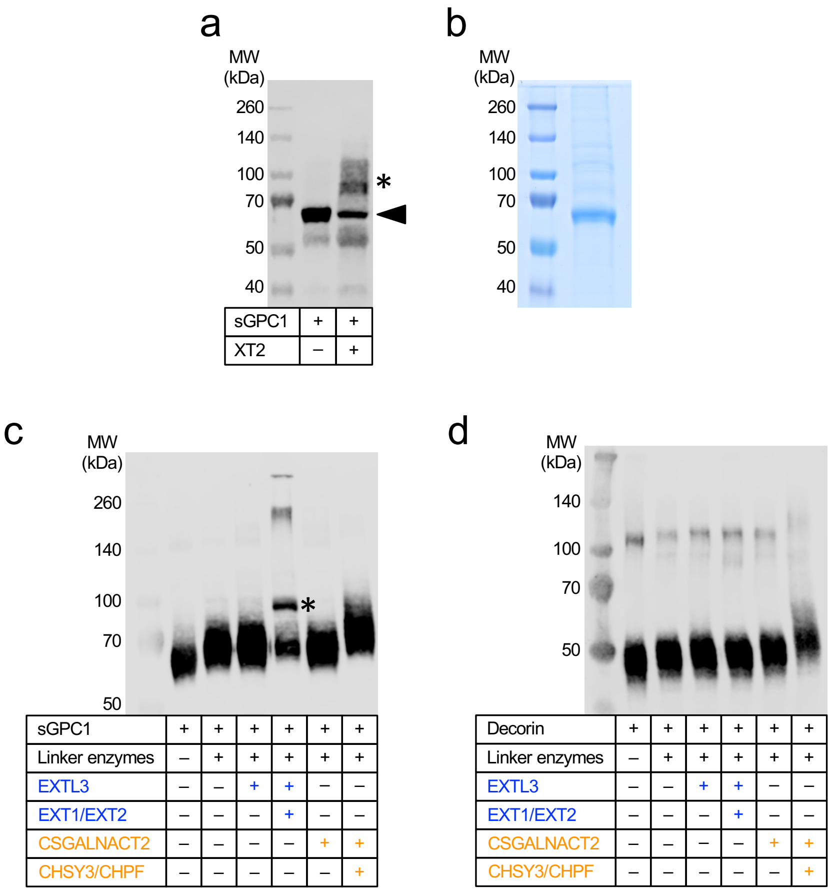

Supplementary Fig. 10 | Polymerisation of HS and CS backbones on folded core proteins.
a Xylosyltransferase-deficient CHO pgsA-745 cells were transfected with a vector encoding soluble glypican-1 with a C-terminal FLAG tag (sGPC1), either alone or together with a vector encoding full-length XT2. sGPC1 was pulled down from the cell culture medium using anti-FLAG sepharose beads. The beads were boiled, and proteins were separated by SDS-PAGE, transferred onto a nitrocellulose membrane, and detected by an anti-FLAG antibody. Molecular weight markers are labelled. The sGPC1 core protein and GAG-modified sGPC1 are indicated by an arrowhead and asterisk, respectively. A representative Western blot from three independent experiments is shown.
b sGPC1 was purified by anti-FLAG affinity chromatography from the supernatant of transfected CHO pgsA-745 cells. An aliquot of the purified protein was boiled and analysed by SDS-PAGE and Coomassie Brilliant Blue staining. Molecular weight markers are labelled. **c** Purified sGPC1 was incubated overnight with the indicated biosynthetic enzymes (linker enzymes: XT1, B4GALT7, B3GALT6, B3GAT3, FAM20B), their cognate UDP-sugars, and ATP. Reaction products were boiled, separated by SDS-PAGE, transferred onto a nitrocellulose membrane, and detected by an anti-FLAG antibody. The asterisk indicates the band corresponding to FLAG-EXT1, which was used in this experiment instead of His-EXT1. Molecular weight markers are labelled. A representative Western blot from three independent experiments is shown. **d** FLAG-tagged decorin was expressed in CHO pgsA-745 cells and analysed as described in c. The faint bands at ≈100 kDa likely are SDS-resistant decorin dimers. A representative Western blot from three independent experiments is shown.

Supplementary Table 1. Enzyme constructs.
ΔCC indicates constructs lacking the coiled coil preceding the catalytic domain(s).

Enzyme	UniProt	Residues	Tag
XT1	Q86Y38	149-959	His-TEV
B4GALT7	Q9UBV7	52-327	His-TEV
B3GALT6	Q96L58	35-329	His-MBP-TEV
B3GAT3	O94766	28-335	His-TEV
FAM20B	O75063	55-409	His-TEV
EXTL3	O43909	52-919	His-TEV
EXTL3 ΔCC	O43909	154-919	His-TEV
CSGALNACT1	Q8TDX6	36-532	His-MBP-TEV
CSGALNACT2	Q8N6G5	33-542	His-TEV
CSGALNACT2 ΔCC	Q8N6G5	116-542	His-TEV
EXT1	Q16394	29-746	FLAG, His-TEV
EXT2	Q93063	47-718	His-TEV
CHSY3	Q70JA7	168-882	His-TEV
CHPF	Q8IZ52	106-775	His-TEV

Supplementary Table 2. Summary of mass spectrometry results.

The modified serine(s) are indicated in bold. Tetra(P) indicates the (phosphorylated) linker tetrasaccharide, GlcA-Gal-Gal-Xyl(2P)-*O*-serine. Ahx, 6-aminohexanoic acid.

Glycopeptide name	Peptide sequence	Figure reference	Ion	M_{calc}	M_{expt}
BKN	AEEEG**S**GGGQW	1b	$[M+Na]^+$	1128.42	1128.36
Xyl-BKN	AEEEG**S**GGGQW	1b	$[M+Na]^+$	1260.46	1260.61
Gal-Xyl-BKN	AEEEG**S**GGGQW	1b	$[M+Na]^+$	1422.51	1422.71
Gal-Xyl2P-BKN	AEEEG**S**GGGQW	S3a, S3b	$[M+Na]^+$	1502.48	1502.60
Gal-Gal-Xyl-BKN	AEEEG**S**GGGQW	1b	$[M+Na]^+$	1584.56	1584.52
Gal-Gal-Xyl2P-BKN	AEEEG**S**GGGQW	S3a, S3a	$[M+Na]^+$	1664.53	1664.63
Tetra-BKN	AEEEG**S**GGGQW	1b	$[M+H]^+$	1738.62	1738.67
TetraP-BKN	AEEEG**S**GGGQW	1b	$[M+Na]^+$	1840.57	1840.47
GlcNAc-Tetra-BKN	AEEEG**S**GGGQW	2a (p)	$[M+H]^+$	1941.70	1941.92
GlcNAc-TetraP-BKN	AEEEG**S**GGGQW	2a (p)	$[M+Na]^+$	2043.65	2043.69
GalNAc-Tetra-BKN	AEEEG**S**GGGQW	2a (p)	$[M+Na]^+$	1963.68	1963.76
GalNAc-TetraP-BKN	AEEEG**S**GGGQW	2a (p)	$[M+Na]^+$	2043.65	2043.69
Xyl-BETA	SPGDS**S**GWPDGYEDLE	S4	$[M+Na]^+$	1864.70	1864.67
Gal-Xyl-BETA	SPGDS**S**GWPDGYEDLE	S4	$[M+Na]^+$	2026.75	2026.72
Gal-Gal-Xyl-BETA	SPGDS**S**GWPDGYEDLE	S4	$[M+Na]^+$	2188.81	2188.75
Tetra-BETA	SPGDS**S**GWPDGYEDLE	S4, 2a (s)	$[M+Na]^+$	2364.84	2364.77
TetraP-BETA	SPGDS**S**GWPDGYEDLE	S4, 2a (s)	$[M+H]^+$	2422.82	2423.27
GlcNAc-Tetra-BETA	SPGDS**S**GWPDGYEDLE	2a (p)	$[M+Na]^+$	2567.92	2568.35
GlcNAc-TetraP-BETA	SPGDS**S**GWPDGYEDLE	2a (p)	$[M+H]^+$	2625.90	2626.21
GalNAc-Tetra-BETA	SPGDS**S**GWPDGYEDLE	2a (p)	$[M+H]^+$	2545.94	2545.49
GalNAc-TetraP-BETA	SPGDS**S**GWPDGYEDLE	2a (p)	$[M+H]^+$	2625.90	2626.17
GlcA-GlcNAc-Tetra-BETA	SPGDS**S**GWPDGYEDLE	S9b (p)	$[M+Na]^+$	2743.95	2744.35
GlcA-GlcNAc-TetraP-BETA	SPGDS**S**GWPDGYEDLE	4b (p)	$[M-H]^-$	2799.92	2799.92
GlcNAc-GlcA-GlcNAc-TetraP-BETA	SPGDS**S**GWPDGYEDLE	4c (p2)	$[M-H]^-$	3003.00	3002.91
Tetra-BETA-Δ	SPGDS**S**GWPDG	S4, S7a (s)	$[M+Na]^+$	1715.58	1715.49
TetraP-BETA-Δ	SPGDS**S**GWPDG	S4, S7a (s)	$[M+Na]^+$	1795.55	1795.45
GlcNAc-TetraP-BETA-Δ	SPGDS**S**GWPDG	S7a (p)	$[M+H]^+$	1976.64	1976.80
GalNAc-Tetra-BETA-Δ	SPGDS**S**GWPDG	S7a (p)	$[M+Na]^+$	1918.66	1918.84
GalNAc-TetraP-BETA-Δ	SPGDS**S**GWPDG	S7a (p)	$[M+H]^+$	1976.64	1976.76
Tetra-BETA-N	SPGDS**S**GWPDGYENLE	S7a (s)	$[M+Na]^+$	2363.85	2363.73
TetraP-BETA-N	SPGDS**S**GWPDGYENLE	3a, S7a (s)	$[M+K]^+$	2459.79	2460.06
GlcNAc-Tetra-BETA-N	SPGDS**S**GWPDGYENLE	S7a (p)	$[M+Na]^+$	2566.93	2567.01
GlcNAc-TetraP-BETA-N	SPGDS**S**GWPDGYENLE	S7a (p)	$[M+H]^+$	2624.92	2624.93
GalNAc-Tetra-BETA-N	SPGDS**S**GWPDGYENLE	S7a (p)	$[M+Na]^+$	2566.93	2567.03
GalNAc-TetraP-BETA-N	SPGDS**S**GWPDGYENLE	S7a (p)	$[M+H]^+$	2624.92	2624.93
Tetra-BETA-Y	SPGDS**S**GYPDGYEDLE	3a	$[M+Na]^+$	2341.82	2342.04
TetraP-BETA-Y	SPGDS**S**GYPDGYEDLE	3a	$[M+Na]^+$	2421.79	2421.97
Tetra-BETA-5-FAM	AhxSPGDS**S**GWPDGYEDLE	S9a	$[M+Na]^+$	2835.96	2834.96
TetraP-BETA-5-FAM	AhxSPGDS**S**GWPDGYEDLE	4a, 4d, 4e	$[M-H]^-$	2891.94	2890.77
GlcNAc-Tetra-BETA-5-FAM	AhxSPGDS**S**GWPDGYEDLE	S9b-f	$[M-H]^-$	3015.05	3013.89
GlcNAc-TetraP-BETA-5-FAM	AhxSPGDS**S**GWPDGYEDLE	4b, 4c, 4f, S9d	$[M-H]^-$	3095.02	3093.93
GalNAc-TetraP-BETA-5-FAM	AhxSPGDS**S**GWPDGYEDLE	4g	$[M-H]^-$	3095.02	3094.05
Tetra-CSPG4	RQGES**S**GDMAWE	S4, S5a (s)	$[M+H]^+$	1984.73	1984.84
TetraP-CSPG4	RQGES**S**GDMAWE	S4, S5a (s)	$[M+H]^+$	2064.70	2064.76
GlcNAc-Tetra-CSPG4	RQGES**S**GDMAWE	S5a (p)	$[M+H]^+$	2187.81	2188.18
GlcNAc-TetraP-CSPG4	RQGES**S**GDMAWE	S5a (p)	$[M+H]^+$	2267.78	2268.09
GalNAc-Tetra-CSPG4	RQGES**S**GDMAWE	S5a (p)	$[M+H]^+$	2187.81	2188.25
GalNAc-TetraP-CSPG4	RQGES**S**GDMAWE	S5a (p)	$[M+H]^+$	2267.78	2268.13

Supplementary Table 2, continued

Glycopeptide name	Peptide sequence	Figure reference	Ion	M_{calc}	M_{expt}
Tetra-SDC2	SIEEA**S**GVYPIDDDD	S4, S5a (s)	$[M+Na]^+$	2278.85	2278.83
TetraP-SDC2	SIEEA**S**GVYPIDDDD	S4, S5a (s)	$[M+K]^+$	2374.79	2374.73
GlcNAc-Tetra-SDC2	SIEEA**S**GVYPIDDDD	S5a (p)	$[M+K]^+$	2497.90	2497.58
GlcNAc-TetraP-SDC2	SIEEA**S**GVYPIDDDD	S5a (p)	$[M+Na]^+$	2561.89	2561.86
GalNAc-Tetra-SDC2	SIEEA**S**GVYPIDDDD	S5a (p)	$[M+Na]^+$	2481.93	2481.77
GalNAc-TetraP-SDC2	SIEEA**S**GVYPIDDDD	S5a (p)	$[M+Na]^+$	2561.89	2561.97
Tetra-SDC2-Δ	SIEEA**S**GVYPI	S7a (s)	$[M+Na]^+$	1818.74	1818.63
TetraP-SDC2-Δ	SIEEA**S**GVYPI	S7a (s)	$[M+Na]^+$	1898.71	1898.57
GlcNAc-TetraP-SDC2-Δ	SIEEA**S**GVYPI	S7a (p)	$[M+Na]^+$	2101.79	2101.98
GalNAc-Tetra-SDC2-Δ	SIEEA**S**GVYPI	S7a (p)	$[M+Na]^+$	2021.82	2022.08
GalNAc-TetraP-SDC2-Δ	SIEEA**S**GVYPI	S7a (p)	$[M+Na]^+$	2101.79	2101.98
Tetra$_2$-SDC4	ESDDFEL**S**G**S**GDLDDLEDW	S4	$[M-H]^-$	3406.19	3406.05
TetraP$_2$-SDC4	ESDDFEL**S**G**S**GDLDDLEDW	S4	$[M-H]^-$	3566.12	3566.19
GlcNAc-Tetra$_2$-SDC4	ESDDFEL**S**G**S**GDLDDLEDW	S5a (p1)	$[M-H]^-$	3609.27	3609.25
GlcNAc$_2$-Tetra$_2$-SDC4	ESDDFEL**S**G**S**GDLDDLEDW	S5a (p2)	$[M-H]^-$	3812.35	3812.32
GalNAc-Tetra$_2$-SDC4	ESDDFEL**S**G**S**GDLDDLEDW	S5a (p1)	$[M-H]^-$	3609.27	3609.20
GalNAc$_2$-Tetra$_2$-SDC4	ESDDFEL**S**G**S**GDLDDLEDW	S5a (p2)	$[M-H]^-$	3812.35	3812.23
Tetra-SDC4-A	ESDDFELAG**S**GDLDDLEDW	S6	$[M+K]^+$	2797.99	2798.06
TetraP-SDC4-A	ESDDFELAG**S**GDLDDLEDW	S6	$[M+H]^+$	2840.00	2839.83
Xyl$_3$-GPC1	DASDDG**S**G**S**G**S**GDGALDDW	S4	$[M+Na]^+$	2201.76	2201.98
(Gal-Xyl)$_3$-GPC1	DASDDG**S**G**S**G**S**GDGALDDW	S4	$[M+Na]^+$	2687.92	2688.14
(Gal-Gal-Xyl)$_3$-GPC1	DASDDG**S**G**S**G**S**GDGALDDW	S4	$[M+Na]^+$	3174.08	3174.28
Tetra$_3$-GPC1	DASDDG**S**G**S**G**S**GDGALDDW	S4	$[M-H]^-$	3678.18	3678.01
TetraP$_3$-GPC1	DASDDG**S**G**S**G**S**GDGALDDW	S4	$[M-H]^-$	3918.08	3917.88
GlcNAc-Tetra$_3$-GPC1	DASDDG**S**G**S**G**S**GDGALDDW	S5a (p1)	$[M-H]^-$	3881.26	3881.06
GalNAc-Tetra$_3$-GPC1	DASDDG**S**G**S**G**S**GDGALDDW	S5a (p1)	$[M-H]^-$	3881.26	3881.06
GalNAc$_2$-Tetra$_3$-GPC1	DASDDG**S**G**S**G**S**GDGALDDW	S5a (p2)	$[M-H]^-$	4084.34	4084.12
GalNAc$_3$-Tetra$_3$-GPC1	DASDDG**S**G**S**G**S**GDGALDDW	S5a (p3)	$[M-H]^-$	4287.42	4287.17

Supplementary Table 3. Crystallographic statistics of EXTL3 structure determinations.

	**Apo enzyme**	**UDP and Mn^{2+} soak**
PDB entry	8OG1	8OG4
**Data Collection**		
Resolution range (Å)	34.90-1.58 (1.62-1.58)	55.16-2.10 (2.14-2.10)
Wavelength (Å)	0.6199	0.9212
Space group	$P3_121$	$P3_221$
a, *b*, *c* (Å)	120.90, 120.90, 127.45	121.62, 121.62, 259.54
α, β, γ (°)	90, 90, 120	90, 90, 120
Unique reflections	146847	129790
Multiplicity	11.1 (6.0)	12.4 (10.9)
Completeness (%)	99.9 (98.7)	100 (100)
Mean $I/\sigma(I)$	14.6 (0.9)	7.4 (0.4)
$CC_{1/2}$	0.999 (0.323)	0.998 (0.255)
R_{pim}	0.024 (0.819)	0.048 (0.986)
**Refinement**		
Non-hydrogen atoms<br>Protein<br>Glycan<br>Water<br>Ligand	 5749<br>114<br>327<br>0	 11434<br>178<br>74<br>52
R_{work}<br>R_{free}	0.184<br>0.211	0.192<br>0.231
Root-mean-square deviations<br>Bonds (Å)<br>Angles (°)	 0.010<br>1.10	 0.013<br>1.29
Average *B*-factors ($Å^2$)<br>Protein<br>Glycan<br>Water<br>Ligand	 39.5<br>68.2<br>34.8 	 71.9<br>86.3<br>53.8<br>82.2
Ramachandran plot<br>Favoured (%)<br>Allowed (%)<br>Outliers (%)	 96.2<br>3.8<br>0	 95.5<br>4.4<br>0.1

Supplementary References

1 Høie, M. H. *et al.* NetSurfP-3.0: accurate and fast prediction of protein structural features by protein language models and deep learning. *Nucleic Acids Res.* **50**, W510-515 (2022).

2 Jumper, J. *et al.* Highly accurate protein structure prediction with AlphaFold. *Nature* **596**, 583-589 (2021).

3 Mirdita, M. *et al.* ColabFold: making protein folding accessible to all. *Nat. Methods* **19**, 679-682 (2022).
