# Supplementary material for: Molecular mechanism of decision-making in glycosaminoglycan biosynthesis
Source: Nat Commun. 2023 Oct 13;14:6425. doi: 10.1038/s41467-023-42236-z (PMC10570366; doi:10.1038/s41467-023-42236-z)
Supplement: Supplementary file 4 — Description of Additional Supplementary Files [file 41467_2023_42236_MOESM4_ESM.pdf]

<u>Description of Additional Supplementary Files</u>

File: Supplementary Data 1

Description: Contains the annotated mass spectra of all peptides and glycopeptides described in this study. The spectra are presented in the order in which the samples are listed in Supplementary Table 2.
