# Supplementary material for: Molecular mechanism of decision-making in glycosaminoglycan biosynthesis
Source: Nat Commun. 2023 Oct 13;14:6425. doi: 10.1038/s41467-023-42236-z (PMC10570366; doi:10.1038/s41467-023-42236-z)

MS-Spectra

All MS-peaks are indicated as monoisotopic mass. Only main product peaks are assigned in this document. Salt adducts were excluded in this document. DHB was used for negative mode spectra recording. DABP and DHB were used for positive mode spectra recording.

BKN

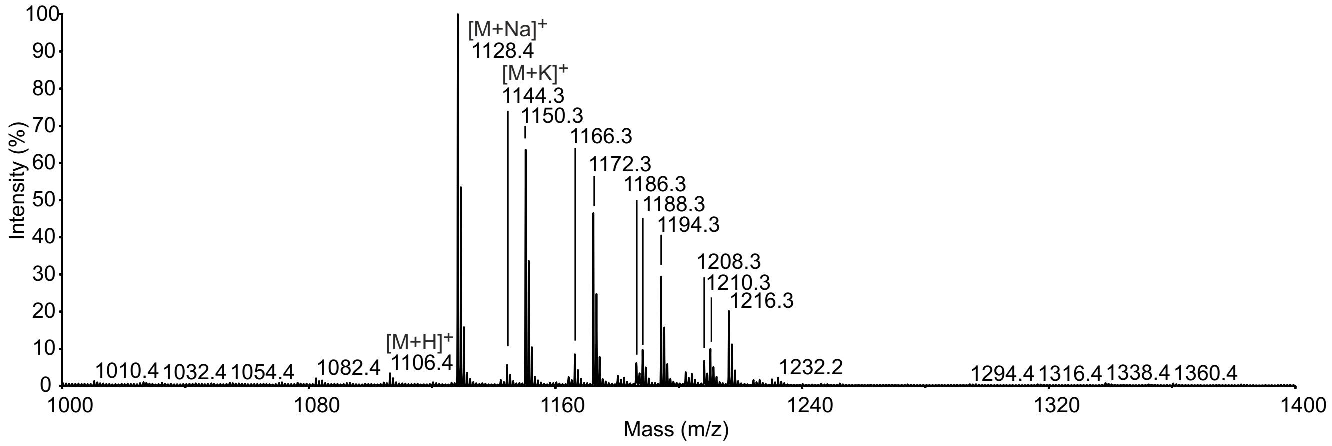

Xyl-BKN

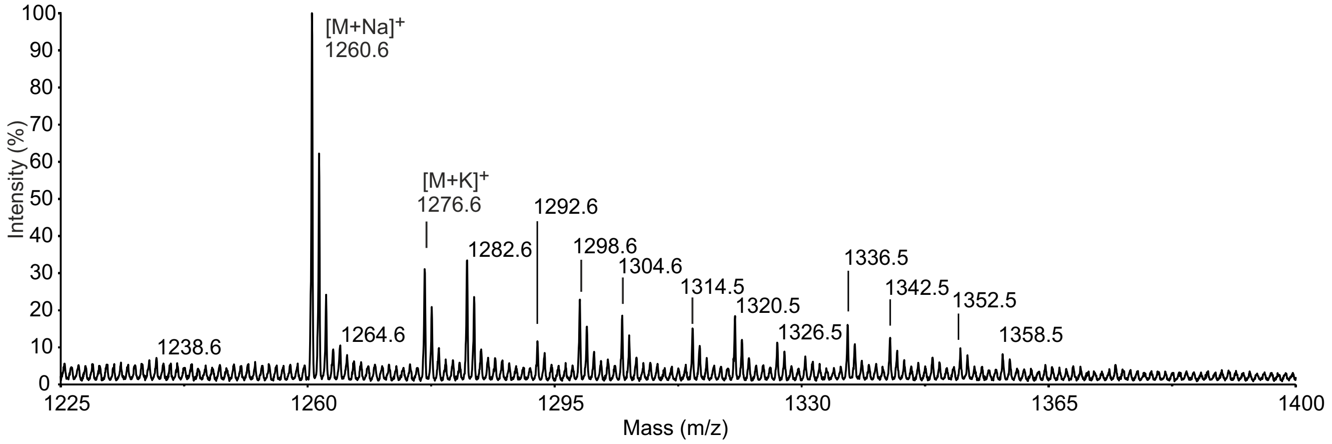

Gal-Xyl-BKN

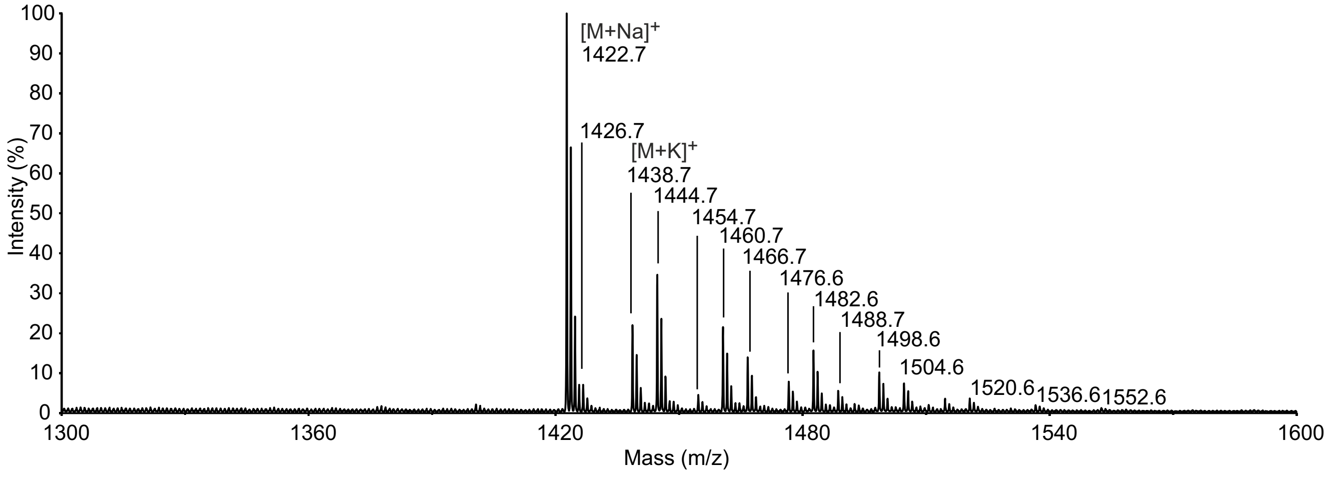

Gal-Xyl2P-BKN

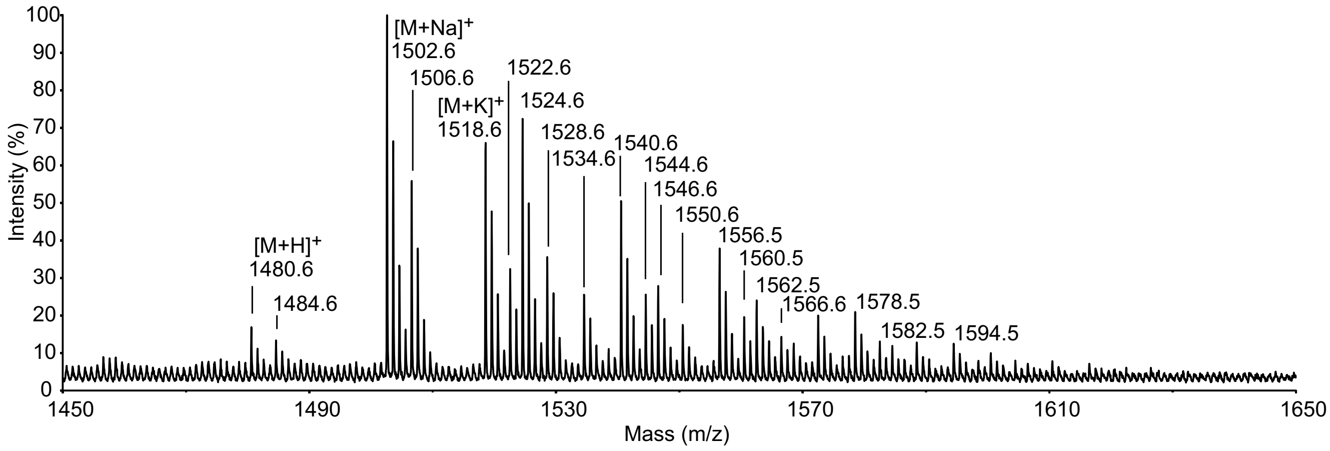

Gal-Gal-Xyl-BKN

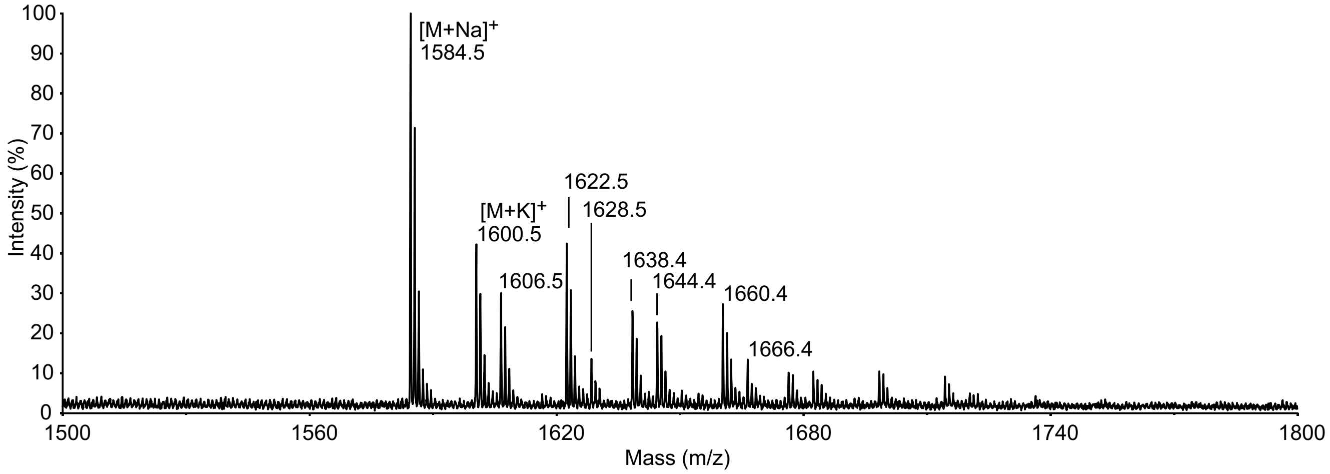

Gal-Gal-Xyl2P-BKN

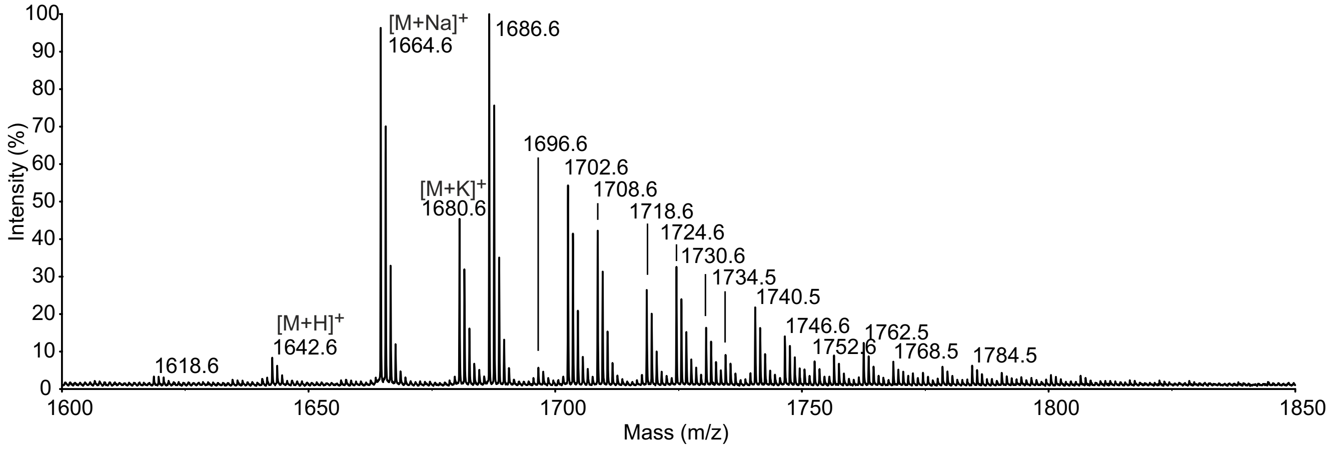

Tetra-BKN

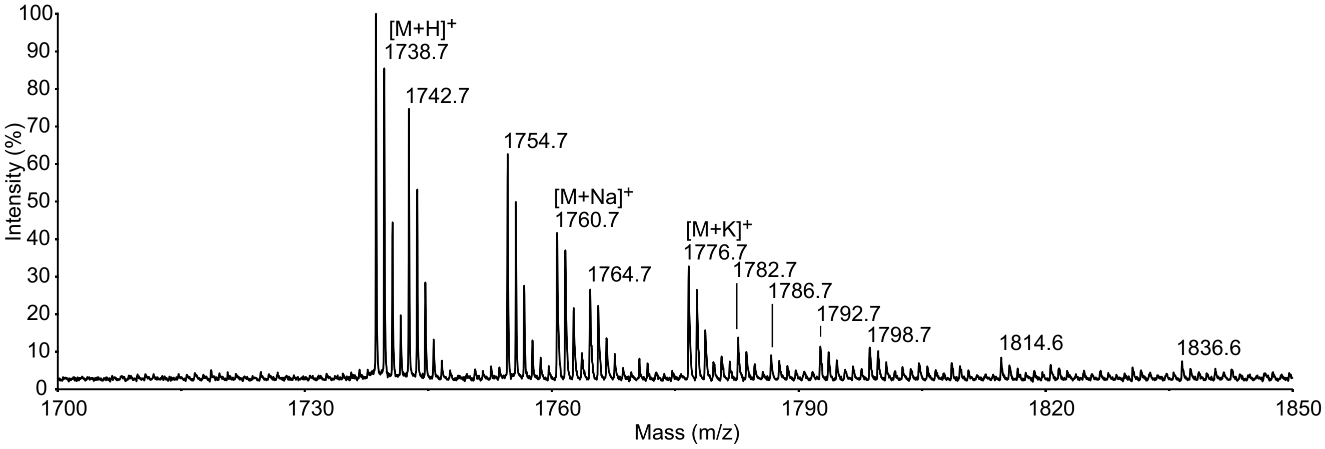

TetraP-BKN

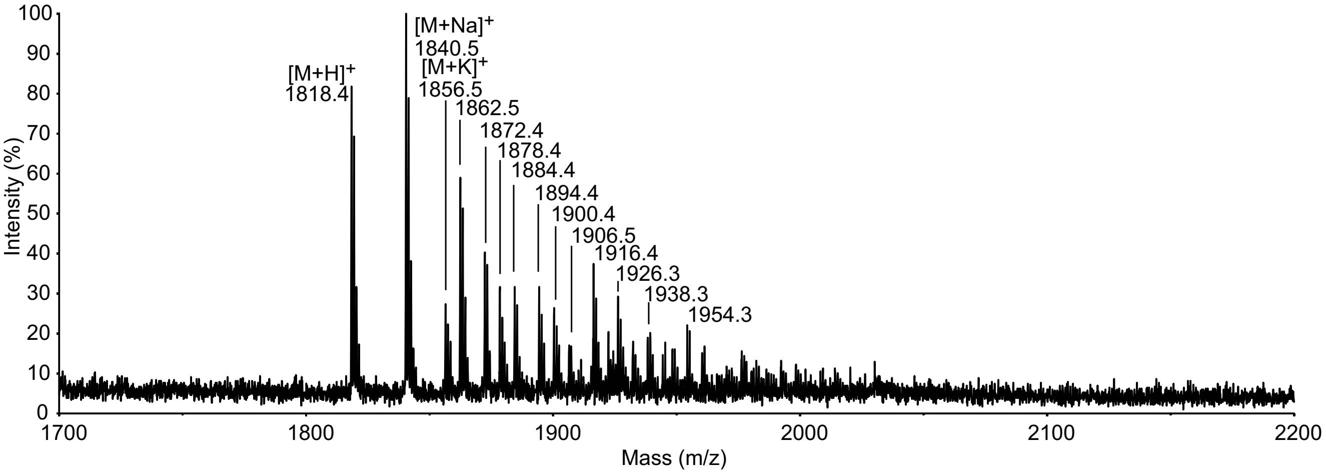

GlcNAc-Tetra-BKN

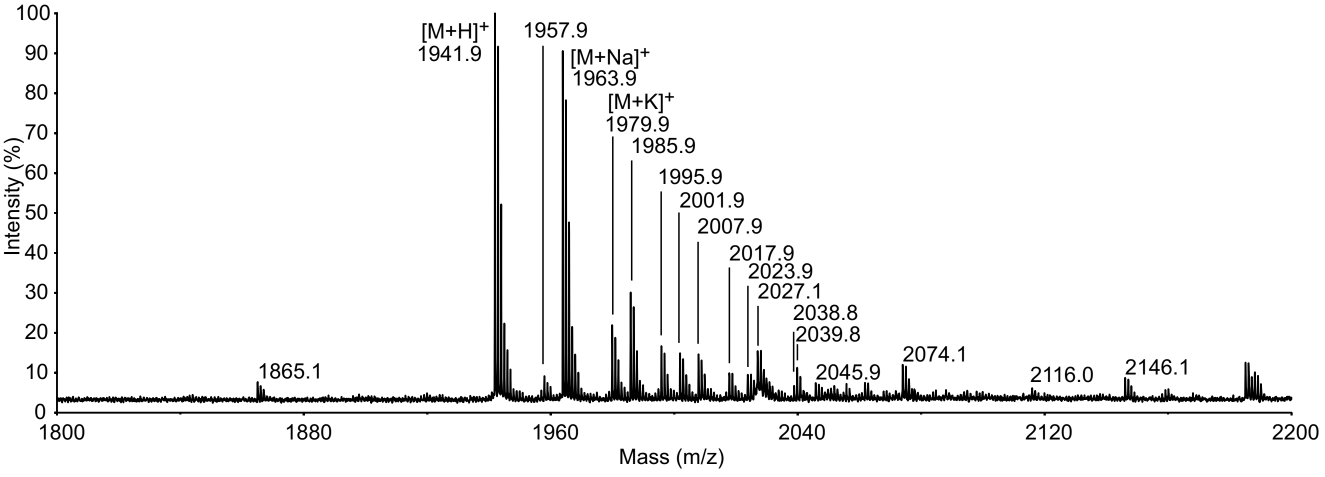

GlcNAc-TetraP-BKN

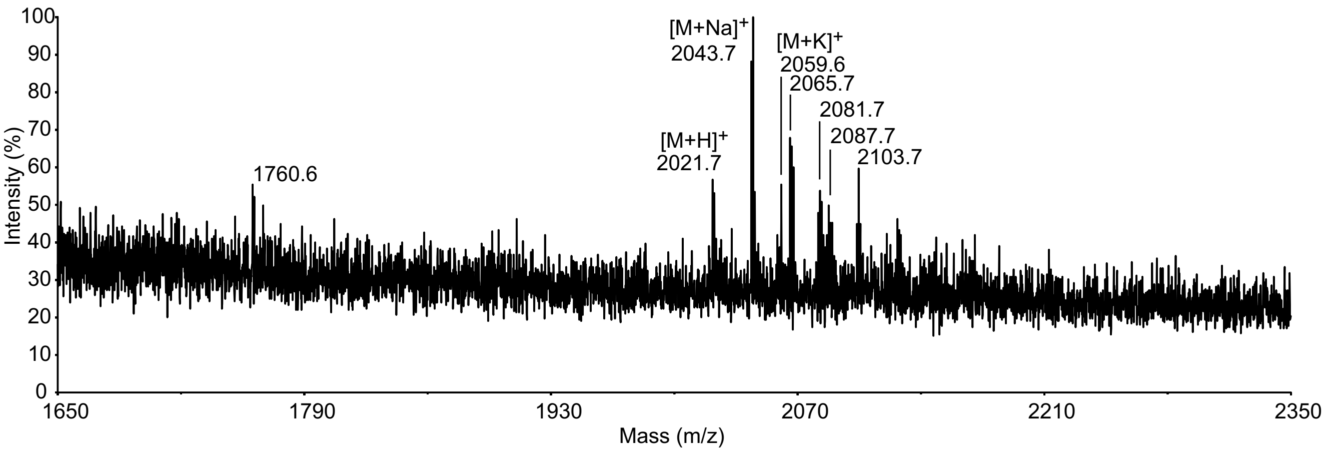

GalNAc-Tetra-BKN

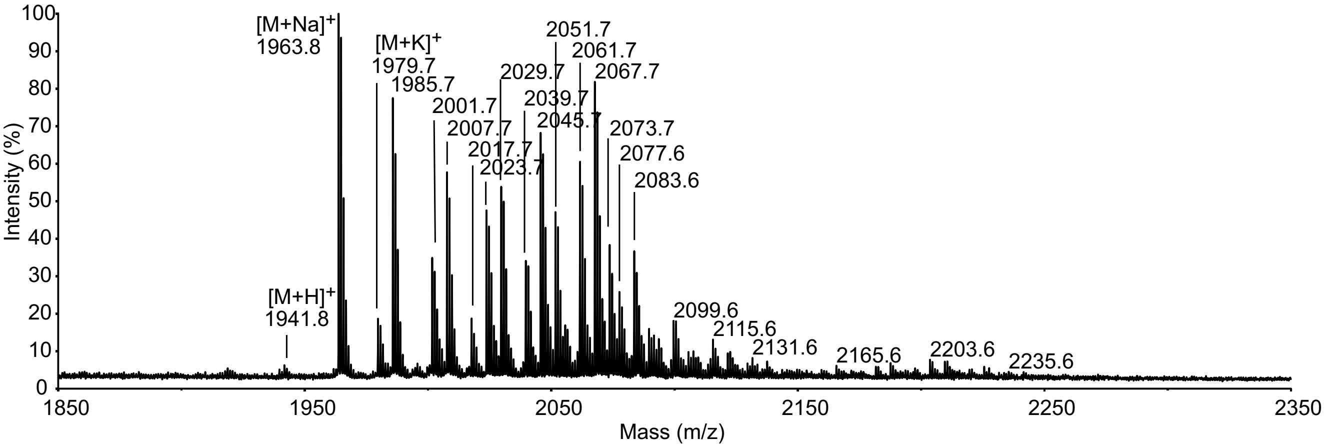

GalNAc-TetraP-BKN

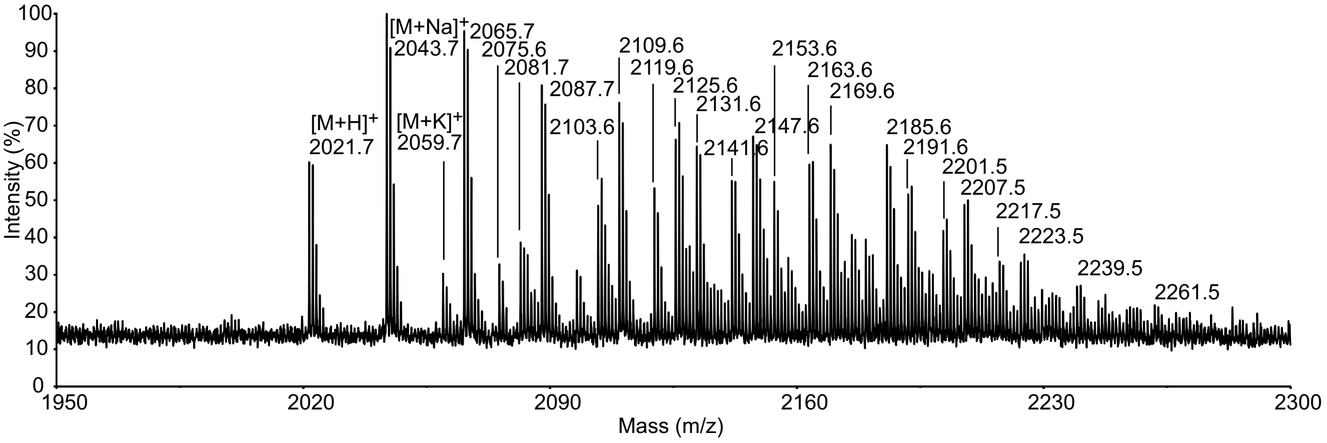

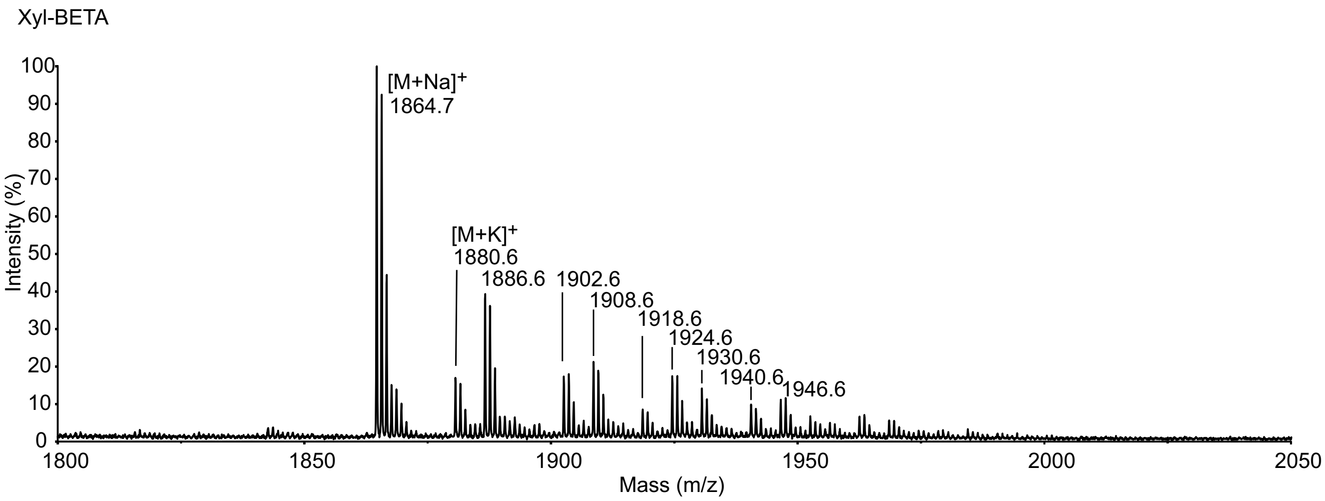
Xyl-BETA
[M+Na]+
1864.7
[M+K]+
1880.6
1886.6
1902.6
1908.6
1918.6
1924.6
1930.6
1940.6
1946.6
Intensity (%)
Mass (m/z)
0
10
20
30
40
50
60
70
80
90
100
1800
1850
1900
1950
2000
2050

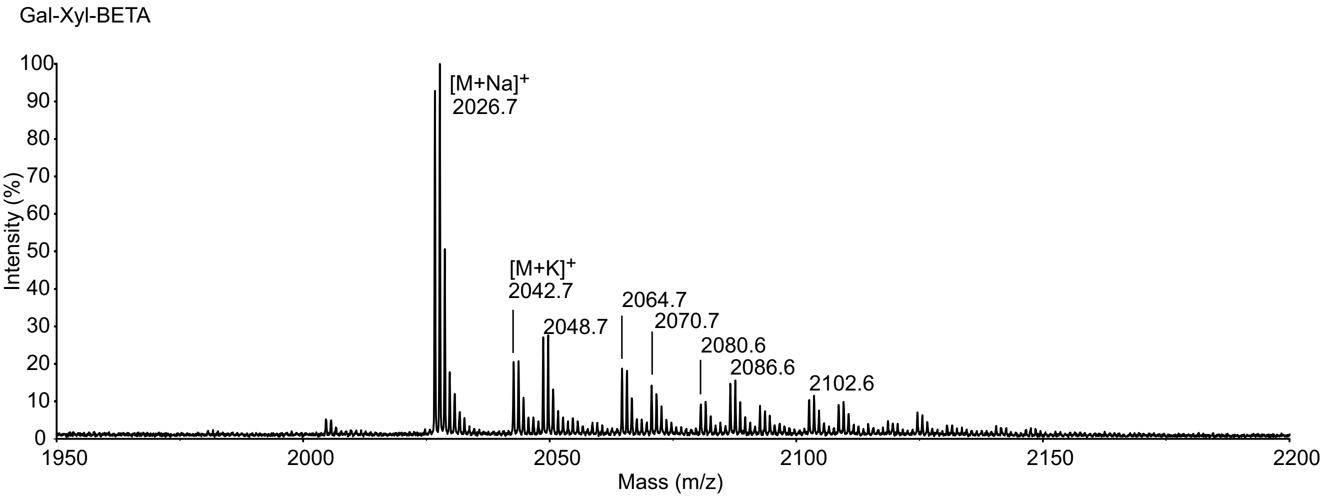
Gal-Xyl-BETA
[M+Na]+
2026.7
[M+K]+
2042.7
2048.7
2064.7
2070.7
2080.6
2086.6
2102.6
Intensity (%)
Mass (m/z)
0
10
20
30
40
50
60
70
80
90
100
1950
2000
2050
2100
2150
2200

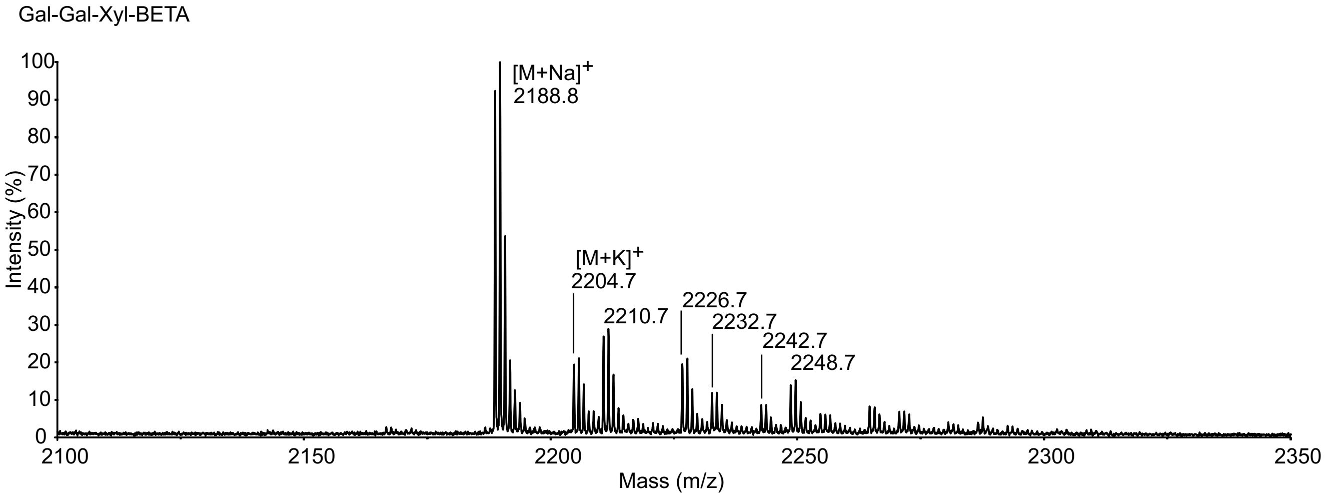
Gal-Gal-Xyl-BETA
[M+Na]+
2188.8
[M+K]+
2204.7
2210.7
2226.7
2232.7
2242.7
2248.7
Intensity (%)
Mass (m/z)
0
10
20
30
40
50
60
70
80
90
100
2100
2150
2200
2250
2300
2350

Tetra-BETA

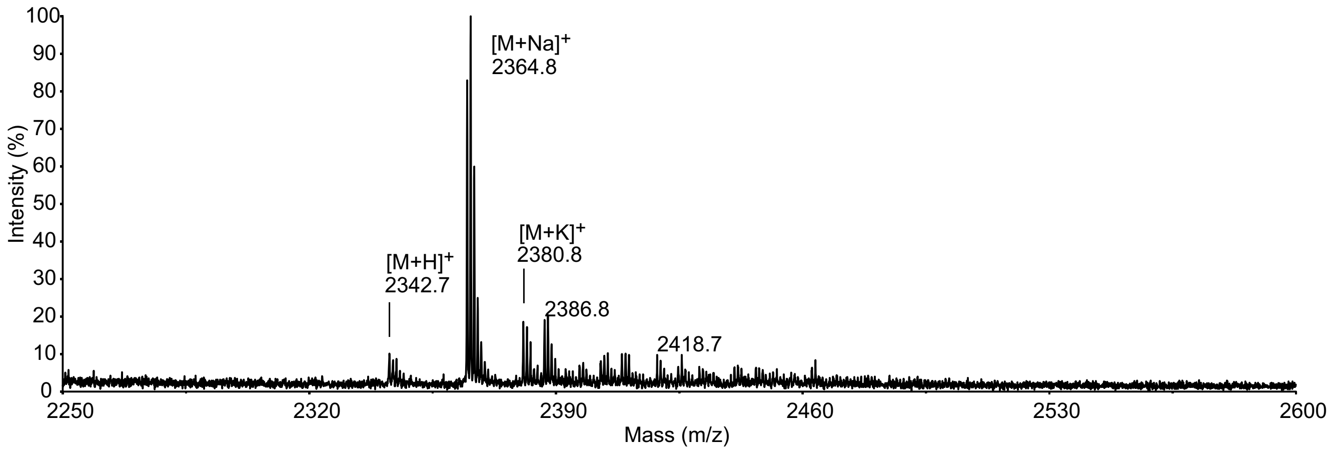

TetraP-BETA

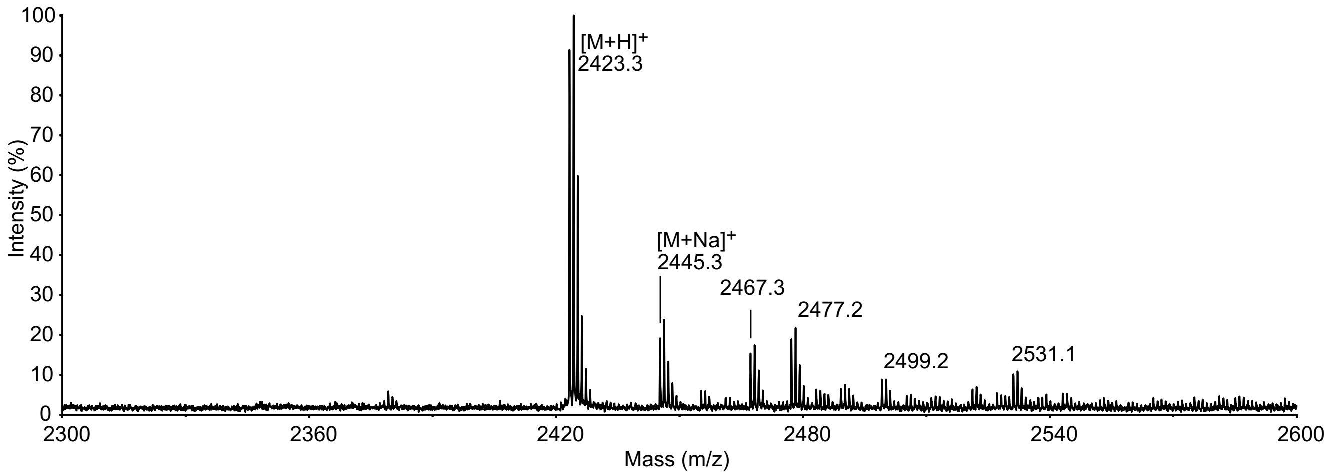

GlcNAc-Tetra-BETA

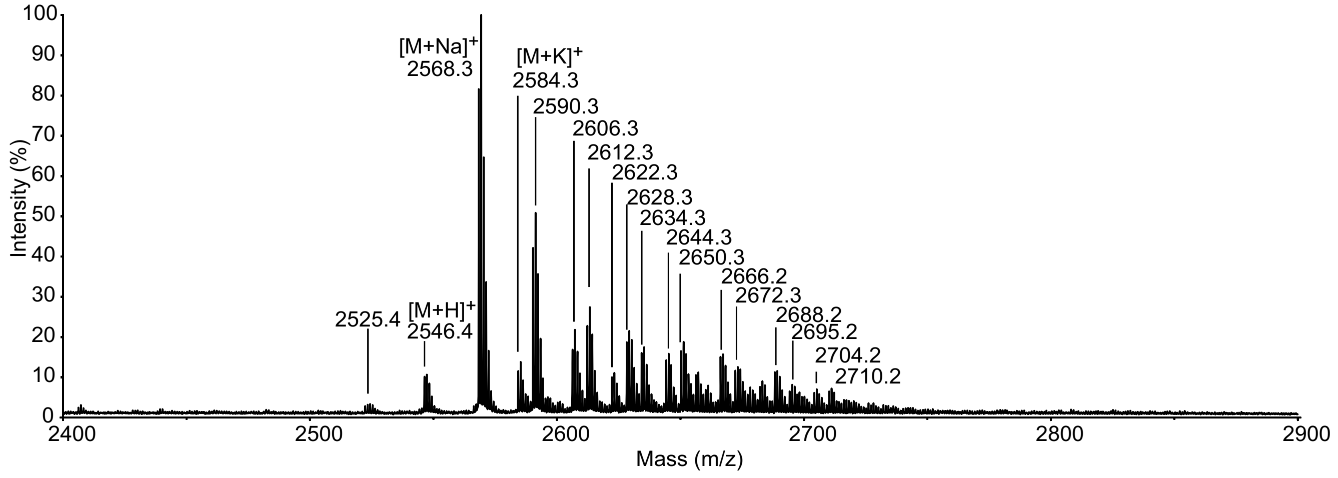

GlcNAc-TetraP-BETA

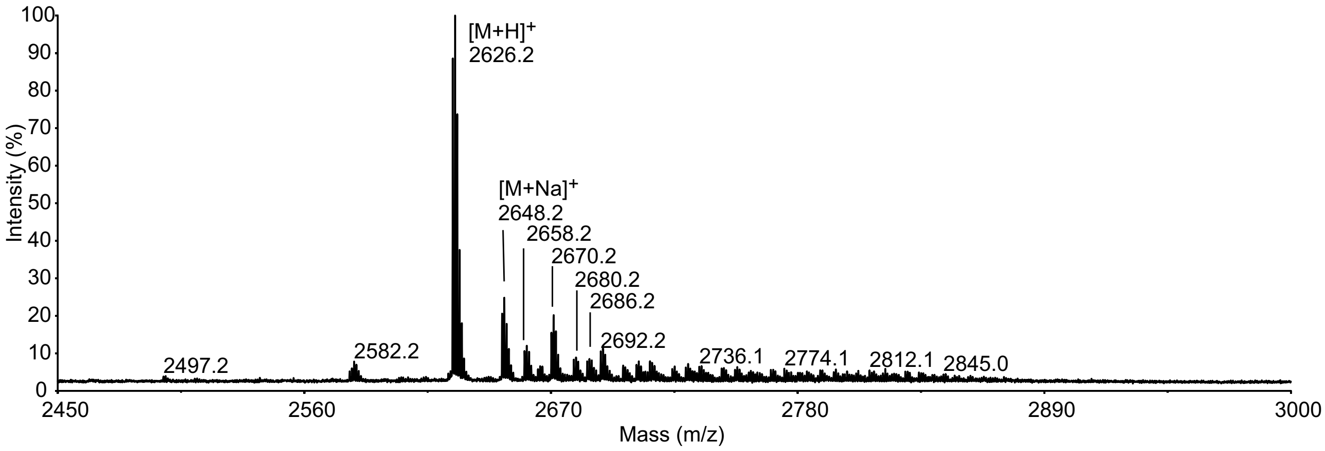

GalNAc-Tetra-BETA

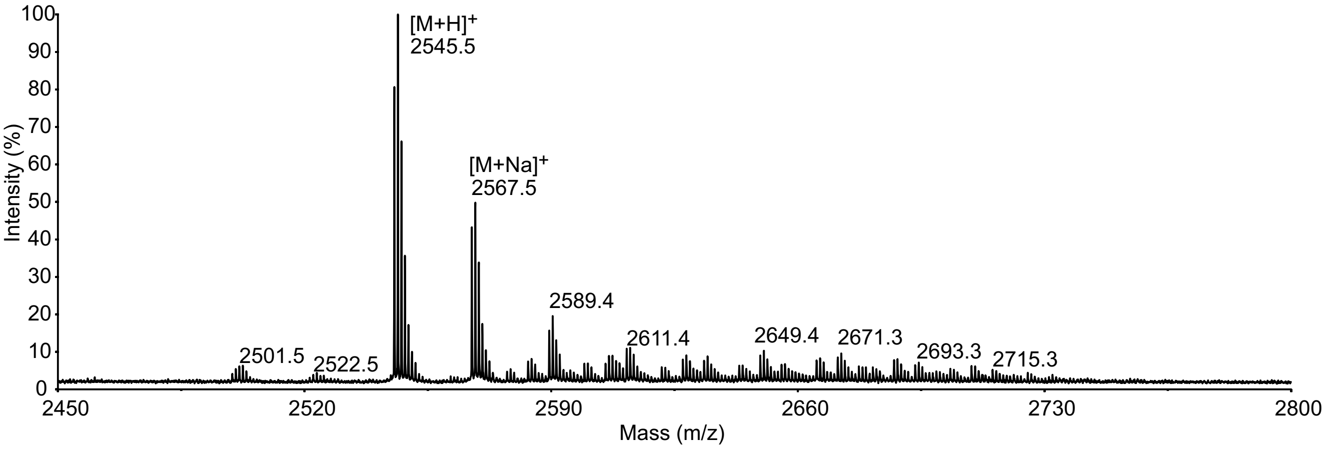

GalNAc-TetraP-BETA

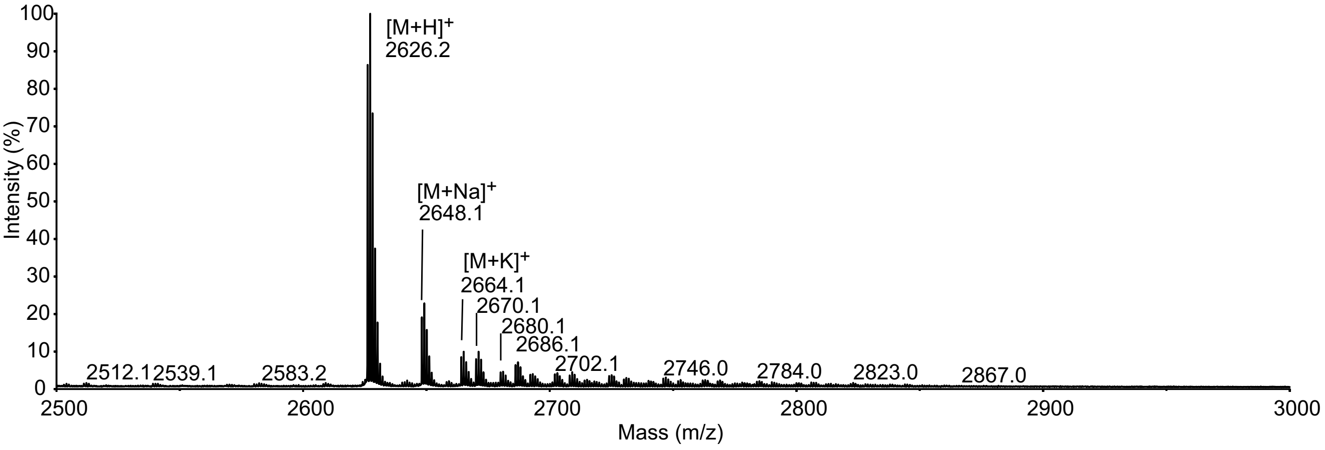

GlcA-GlcNAc-Tetra-BETA

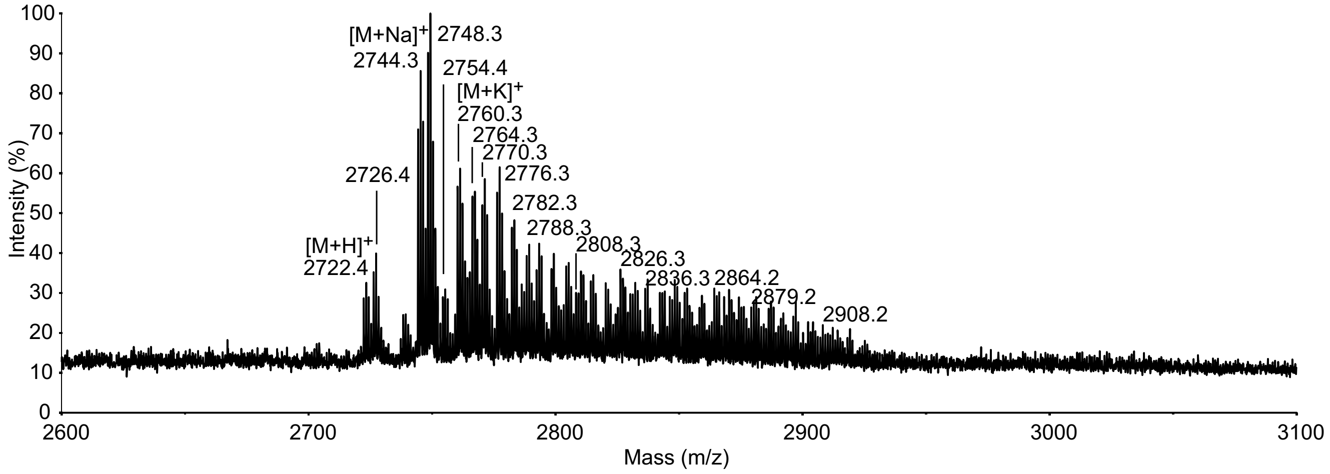

GlcA-GlcNAc-TetraP-BETA

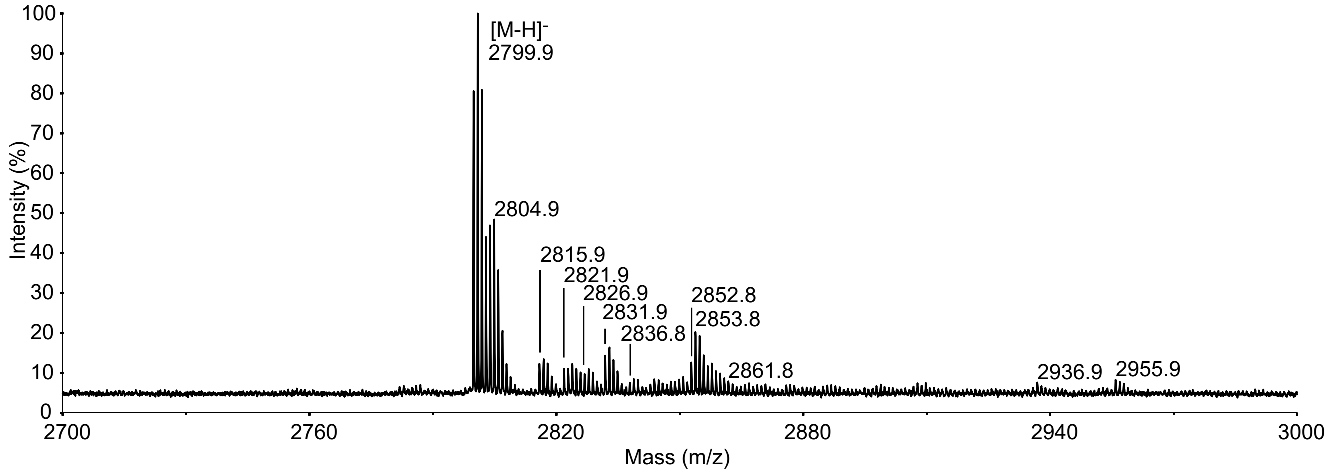

GlcNAc-GlcA-GlcNAc-TetraP-BETA

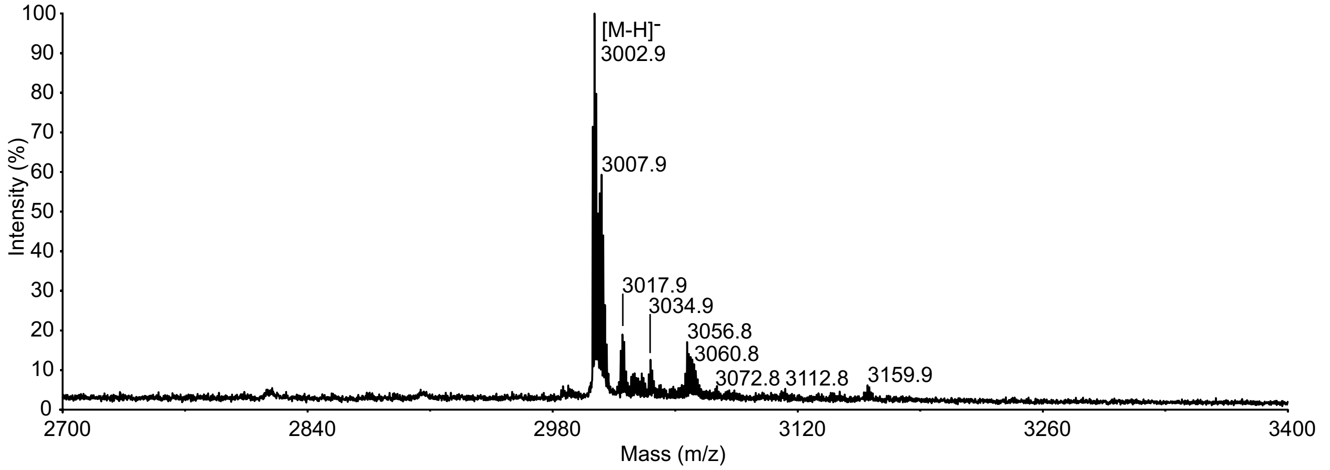

Tetra-BETA-Δ

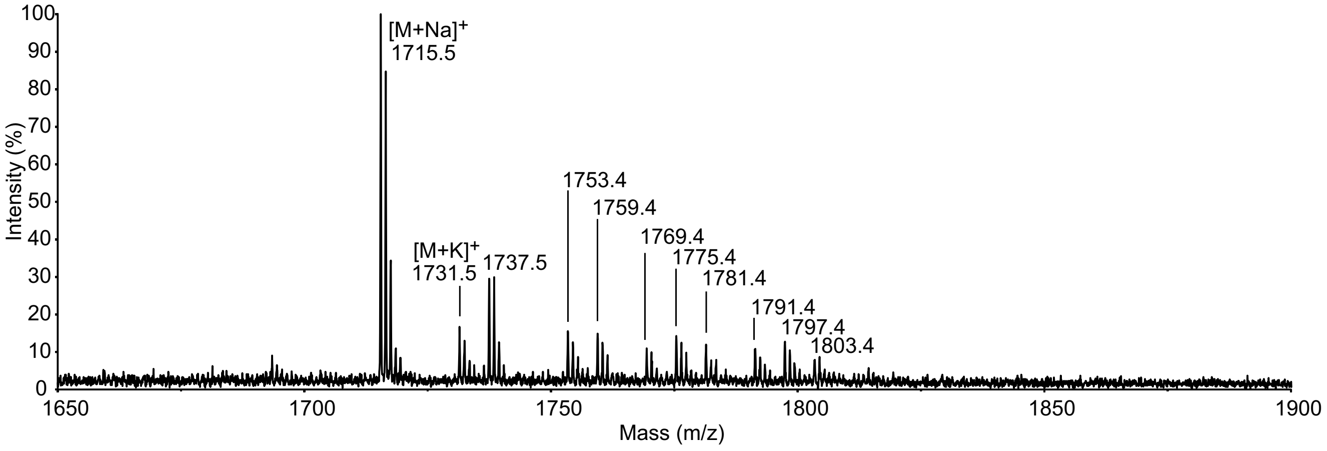

TetraP-BETA-Δ

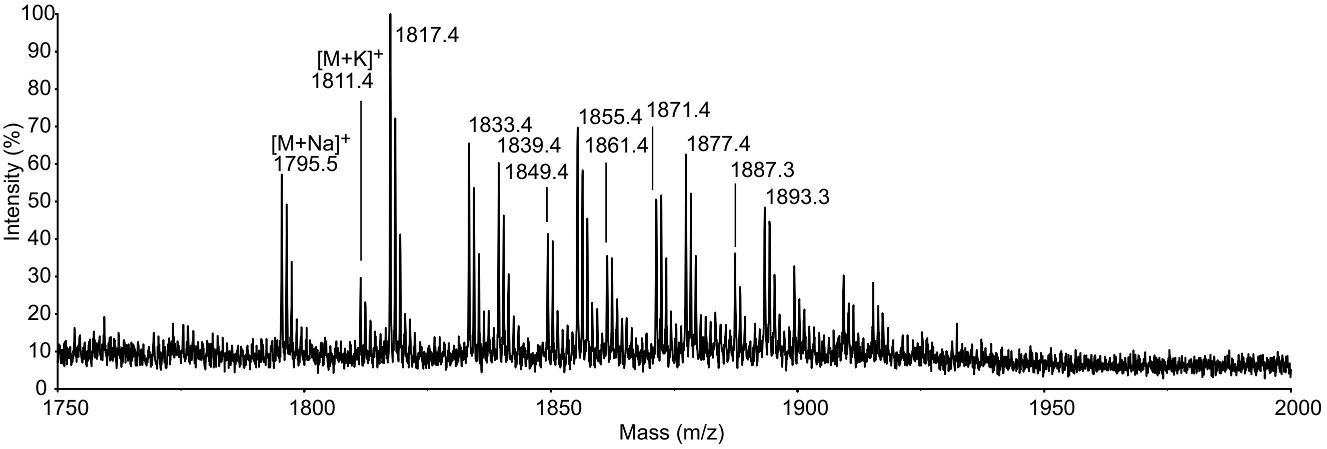

GlcNAc-TetraP-BETA-Δ

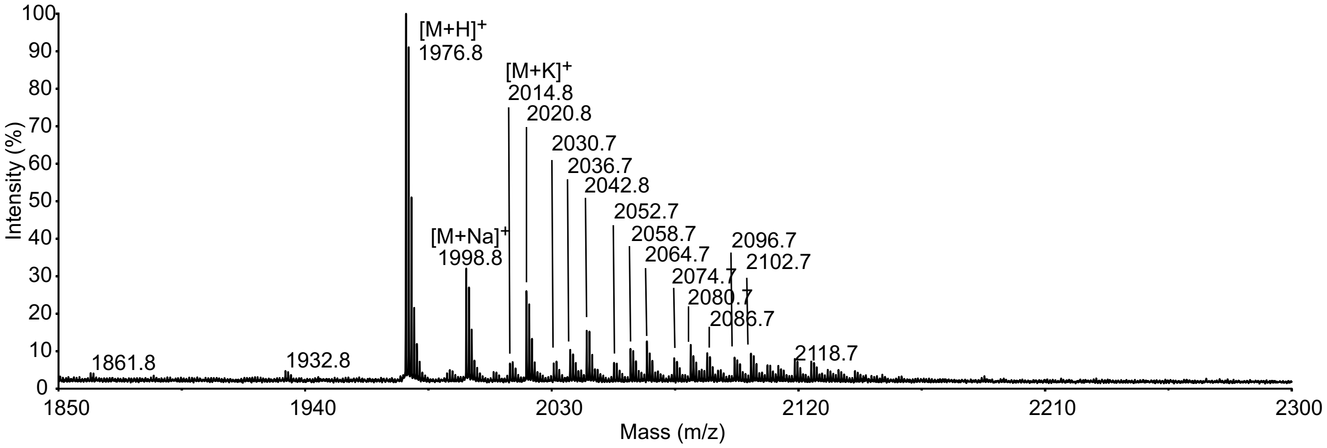

GalNAc-Tetra-BETA-Δ

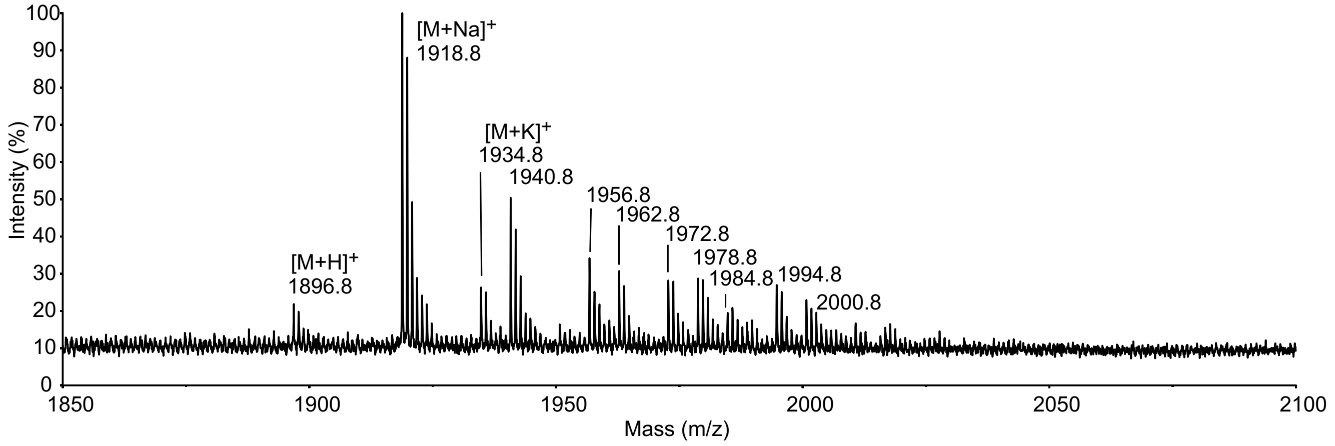

GalNAc-TetraP-BETA-Δ

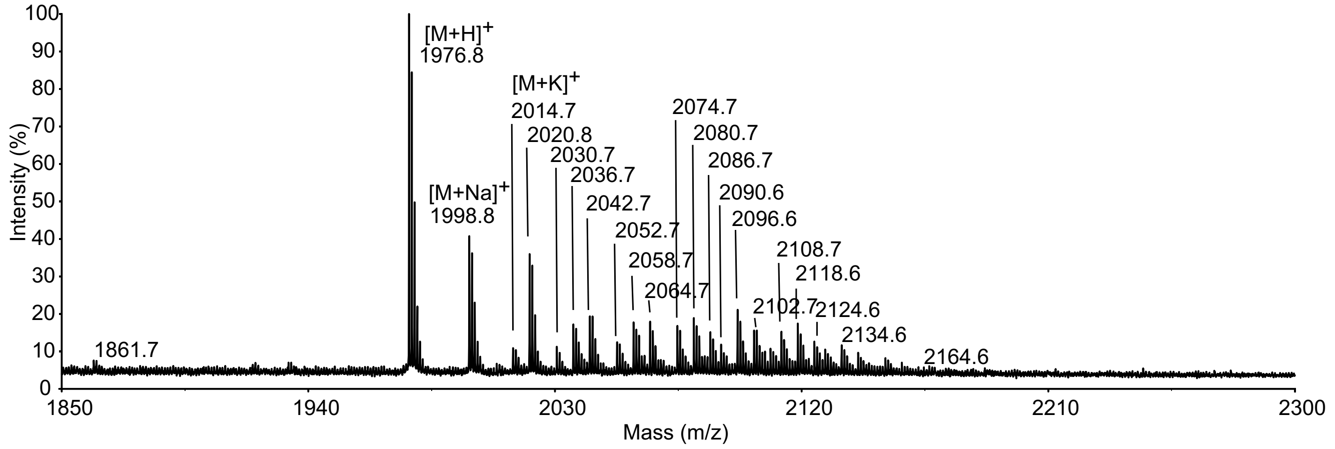

Tetra-BETA-N

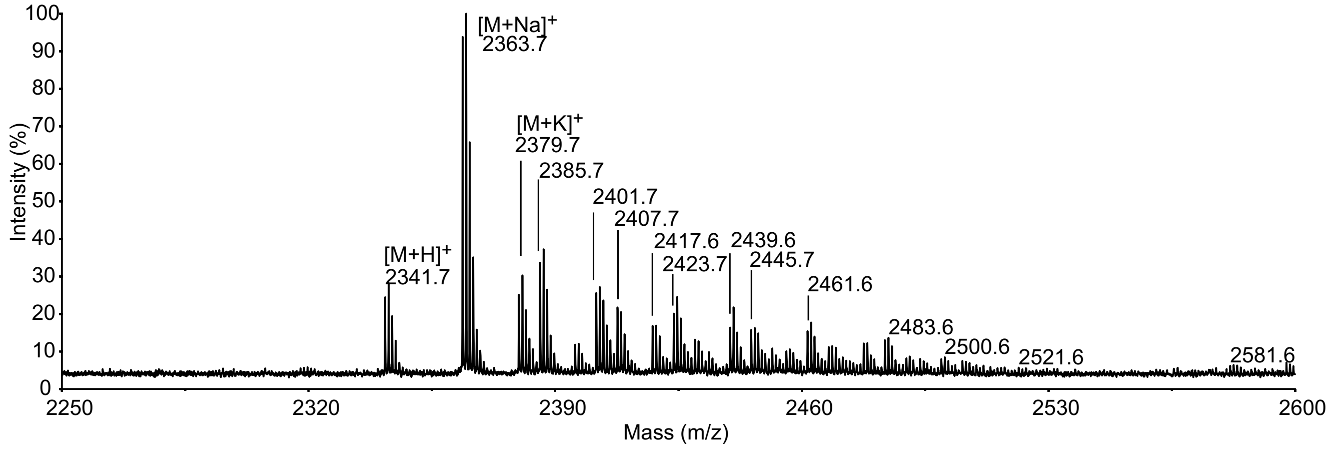

TetraP-BETA-N

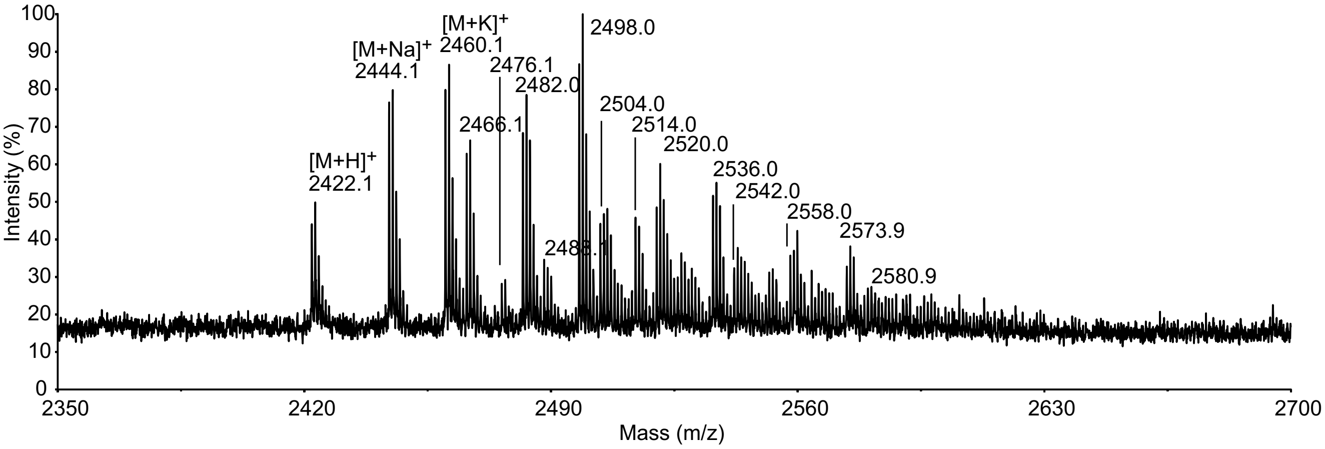

GlcNAc-Tetra-BETA-N

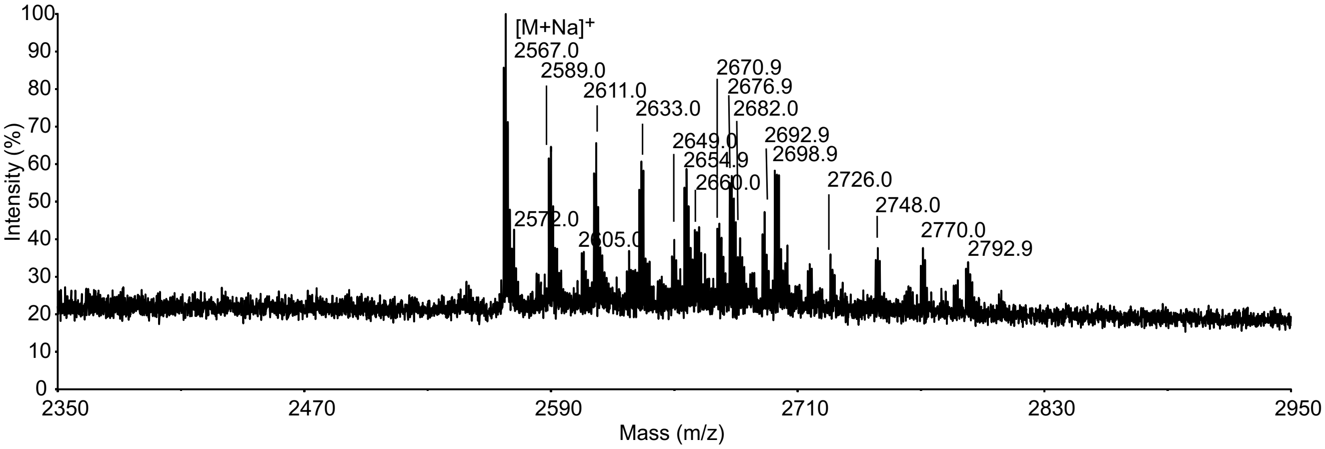

GlcNAc-TetraP-BETA-N

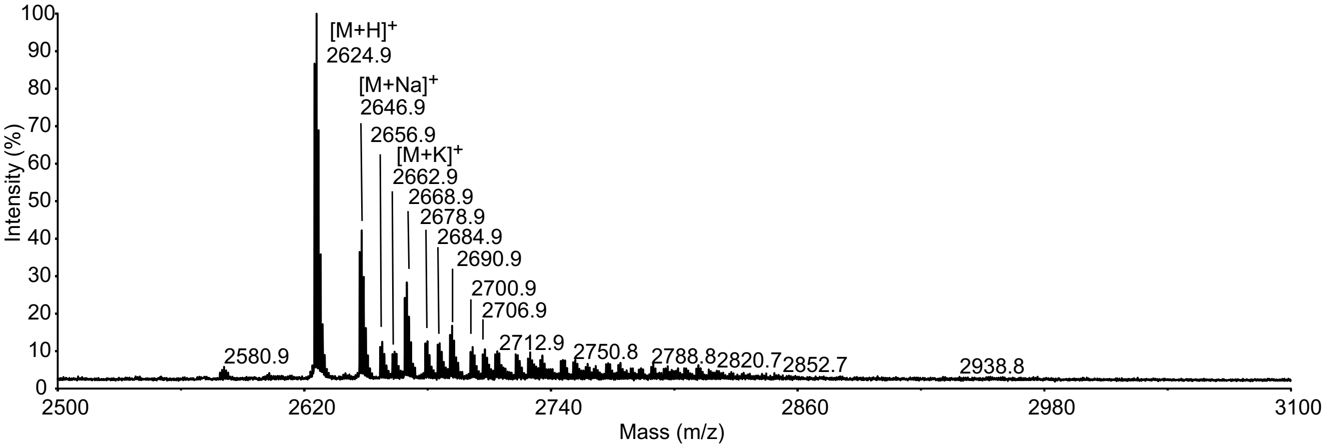

GalNAc-Tetra-BETA-N

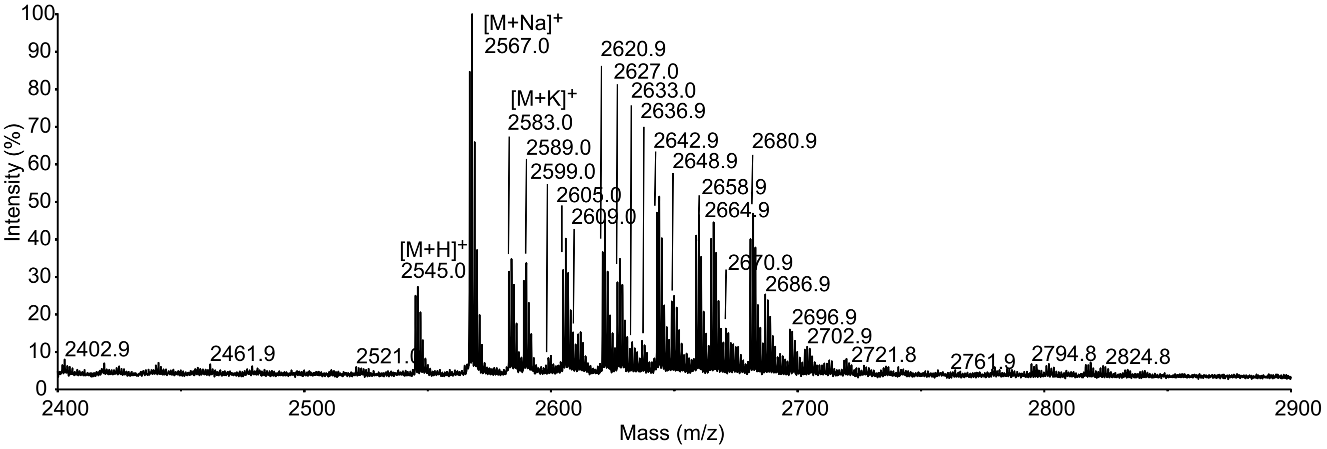

GalNAc-TetraP-BETA-N

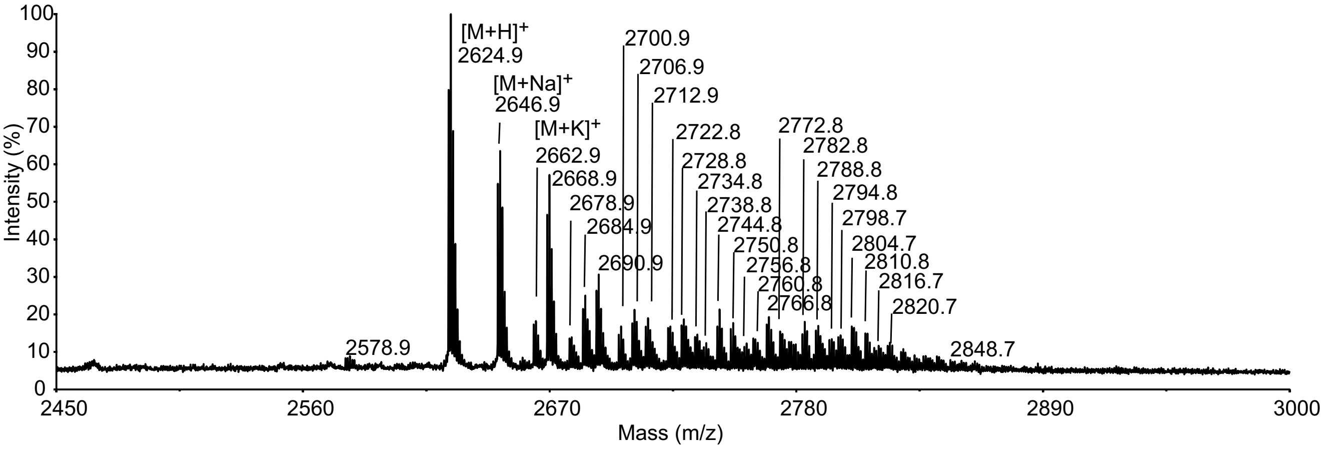

Tetra-BETA-Y

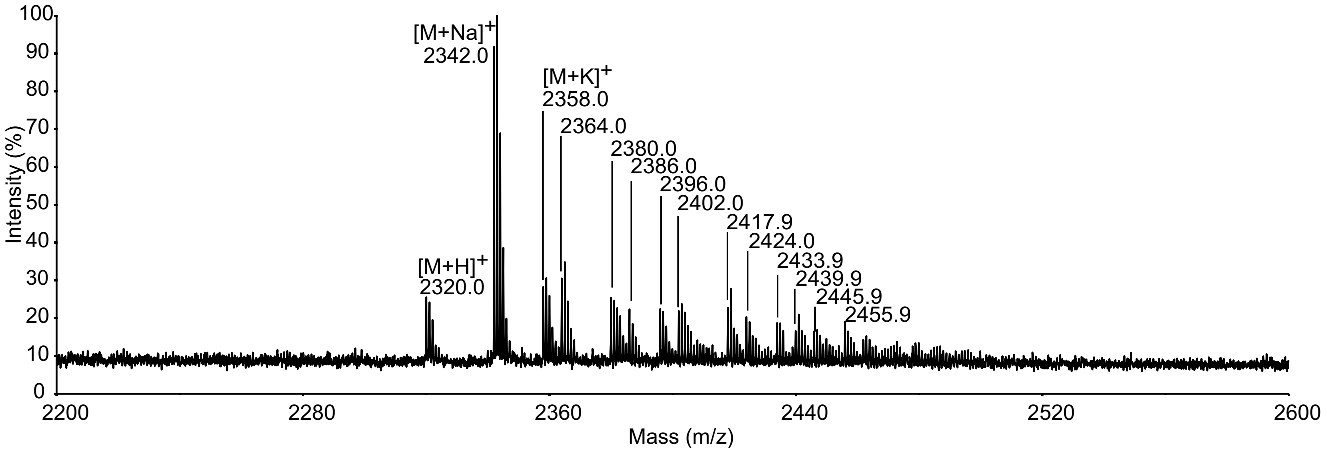

TetraP -BETA-Y

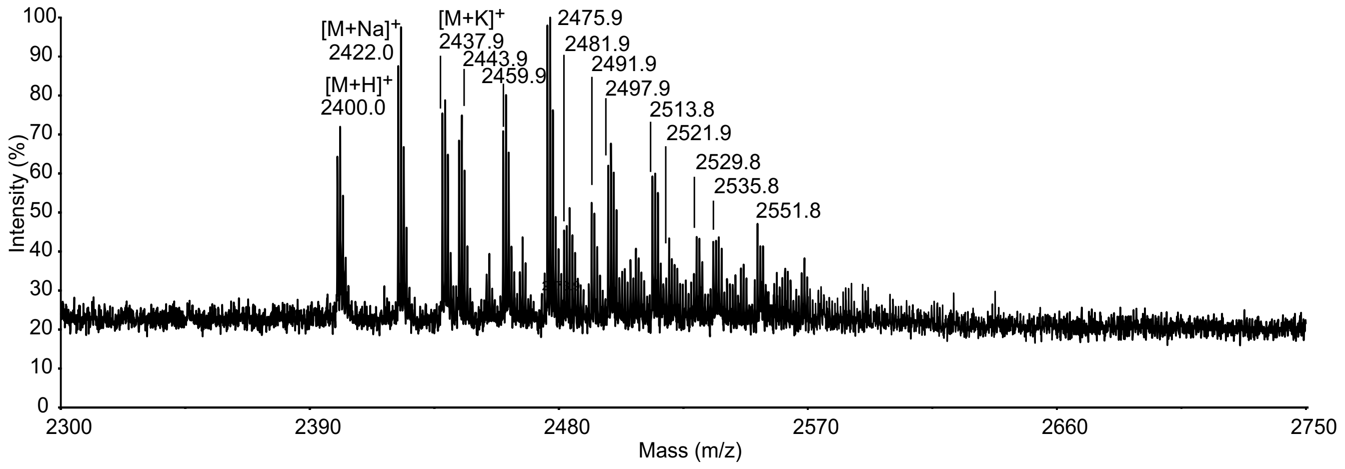

Tetra-BETA-Ahx-5-FAM

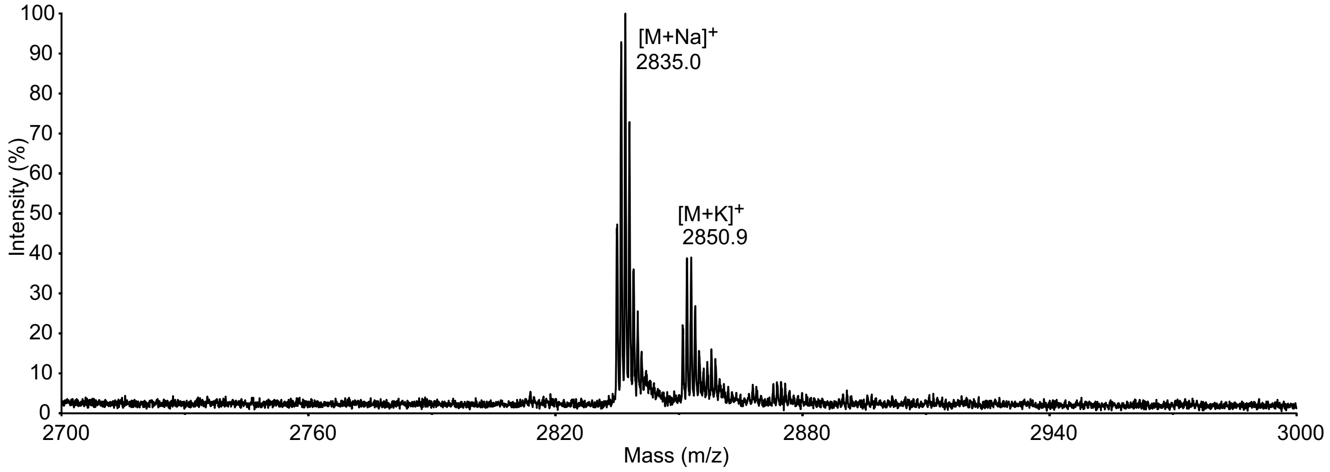

TetraP-BETA-Ahx-5-FAM

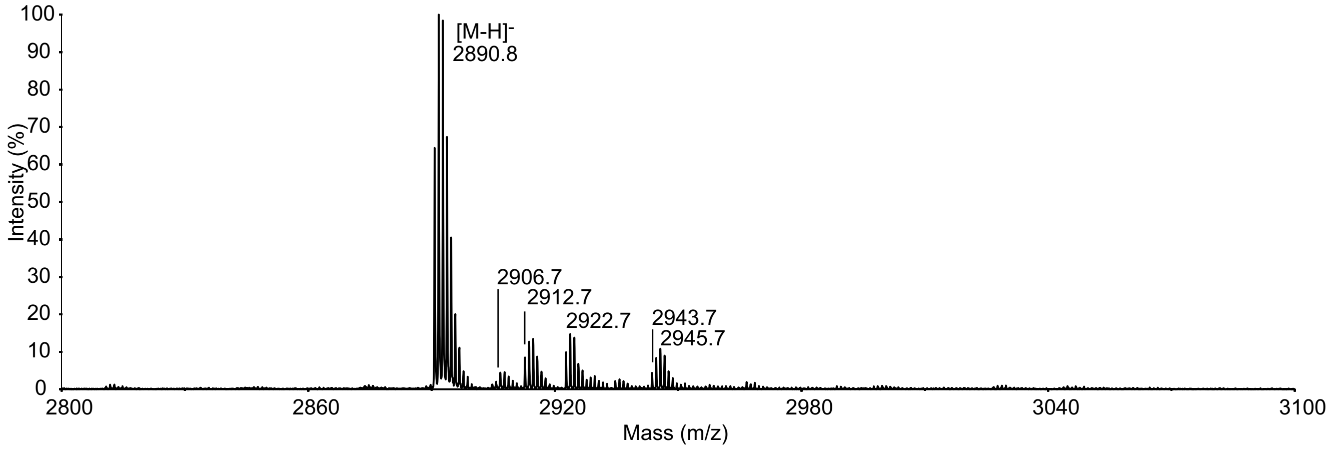

GlcNAc-Tetra-BETA-Ahx-5-FAM

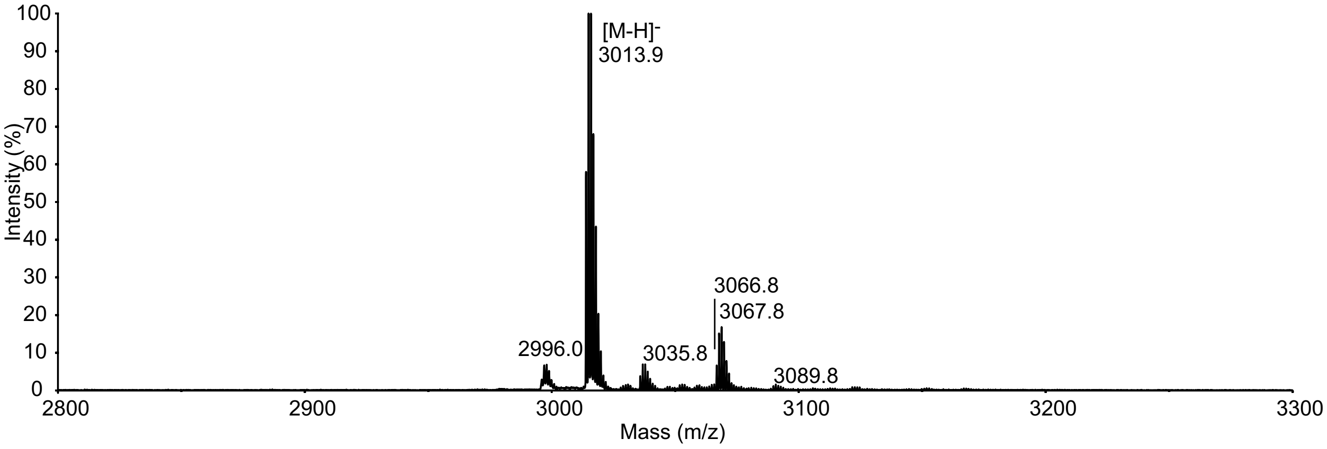

GlcNAc-TetraP-BETA-Ahx-5-FAM

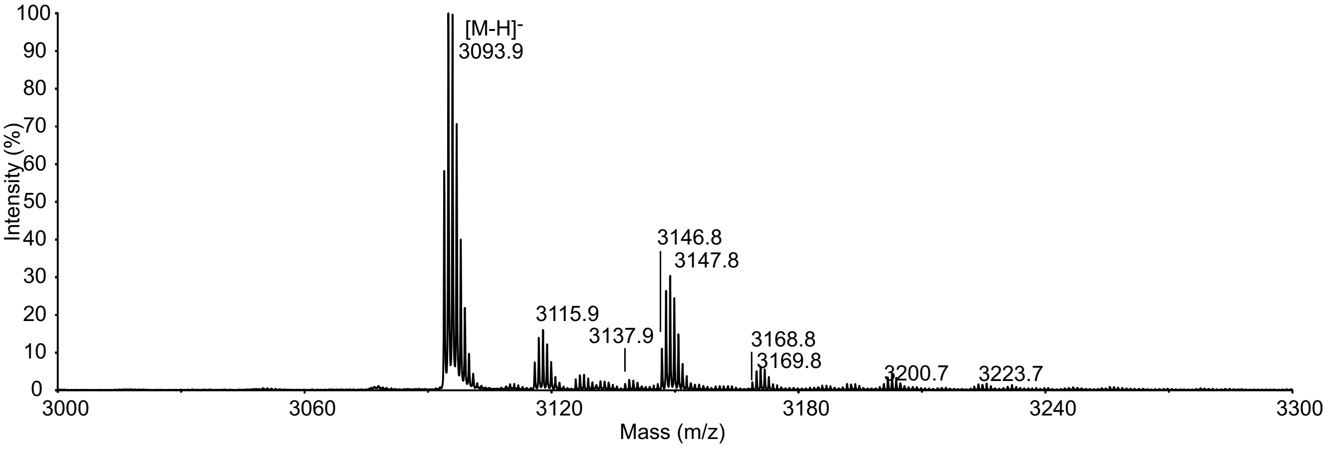

GalNAc-TetraP-BETA-Ahx-5-FAM

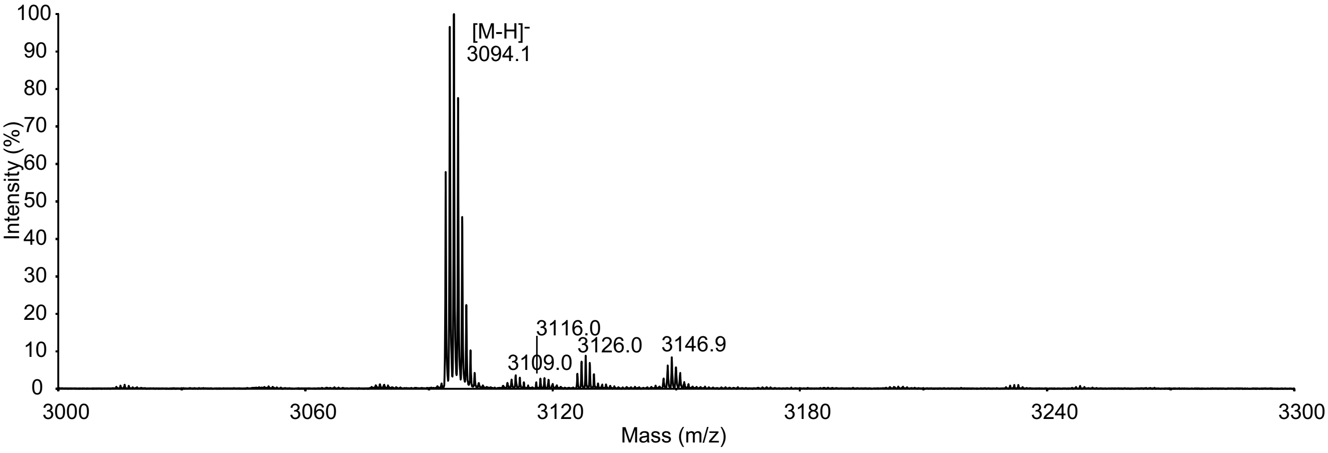

Tetra-CSPG4

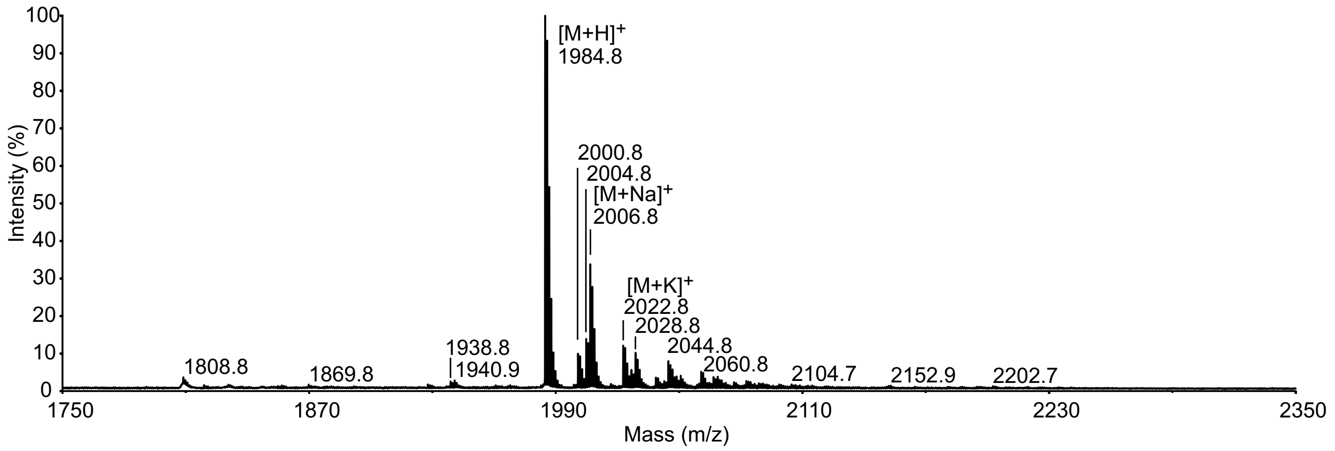

TetraP-CSPG4

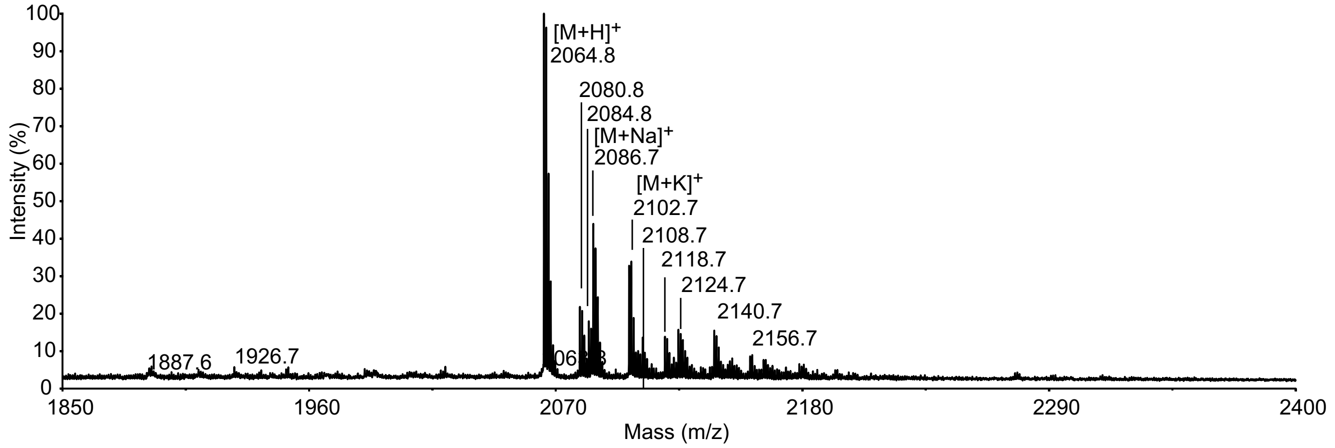

GlcNAc-Tetra-CSPG4

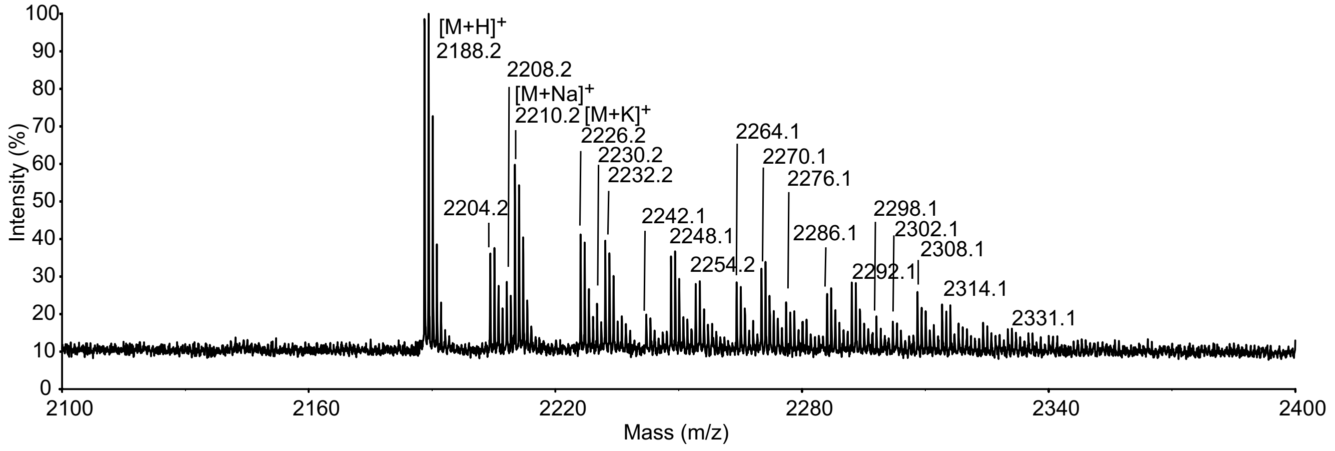

GlcNAc-TetraP-CSPG4

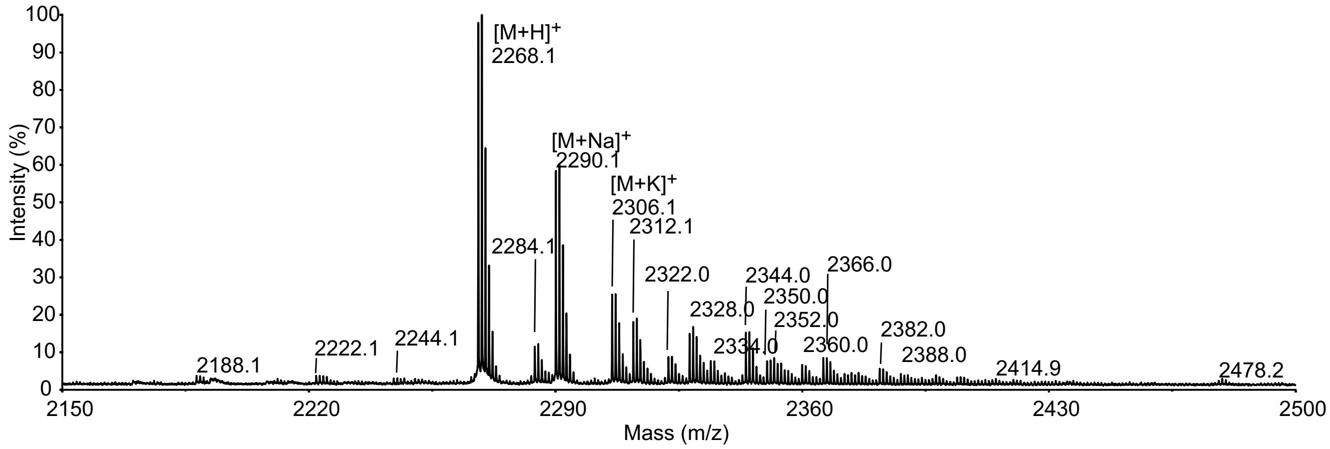

GalNAc-Tetra-CSPG4

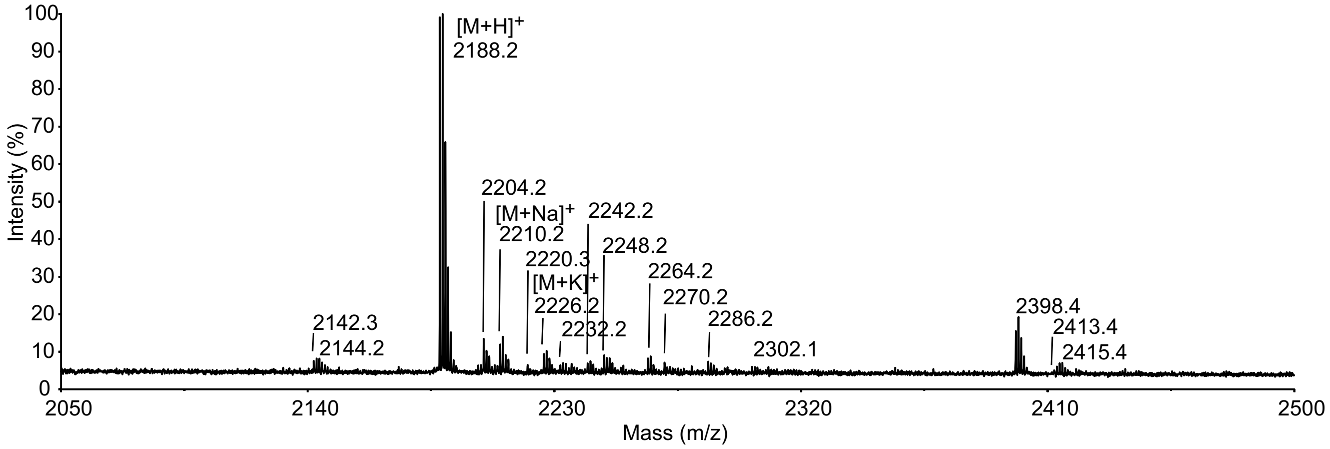

GalNAc-TetraP-CSPG4

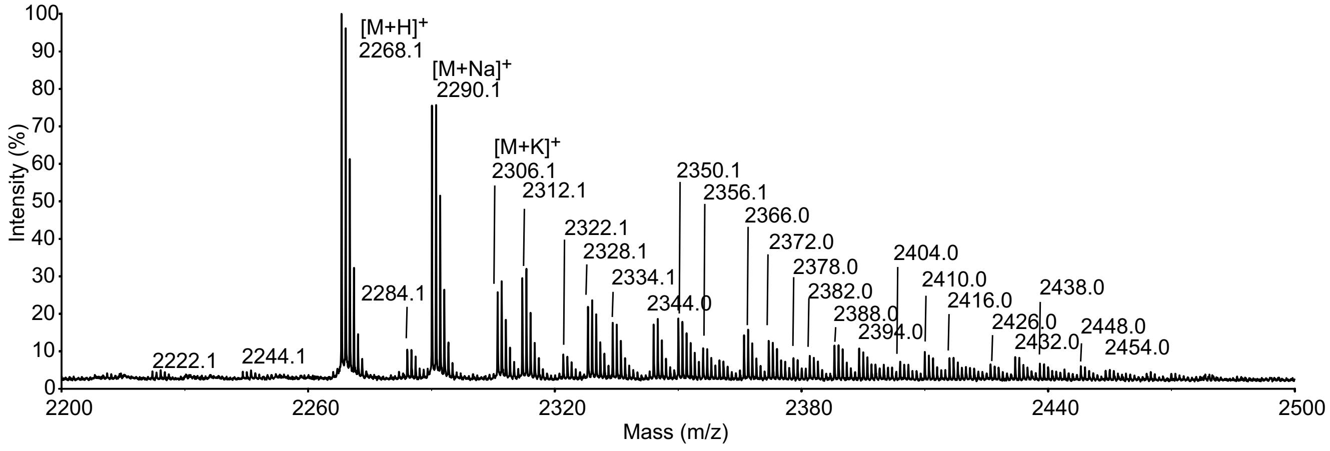

Tetra-SDC2

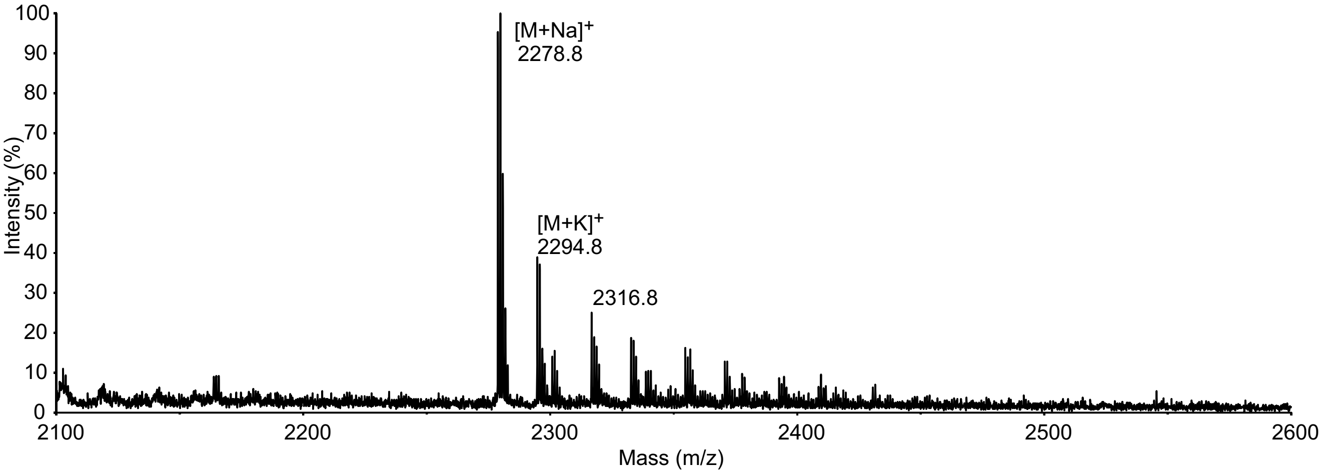

TetraP-SDC2

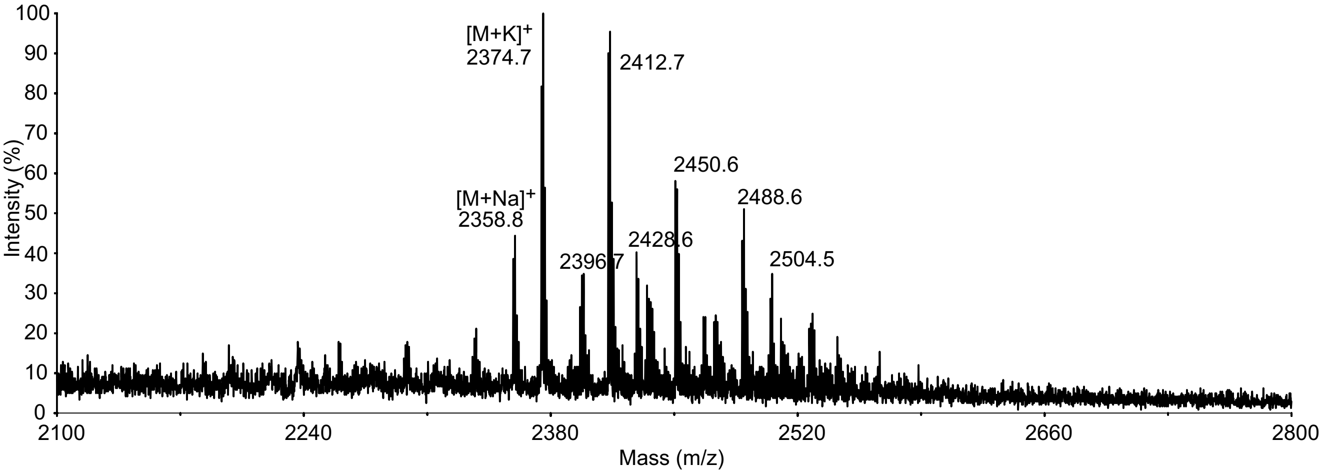

GlcNAc-Tetra-SDC2

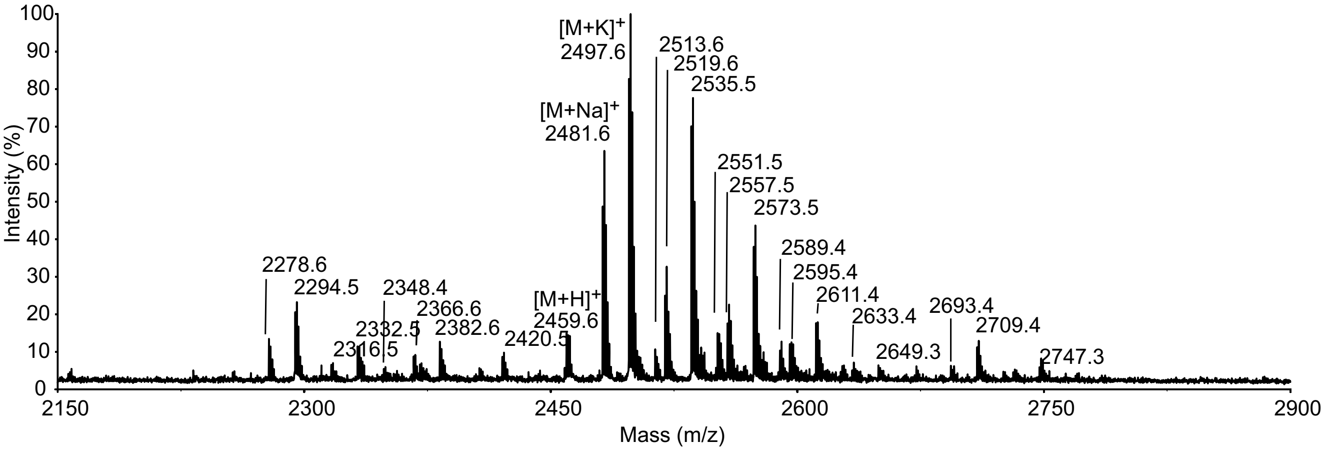

GlcNAc-TetraP-SDC2

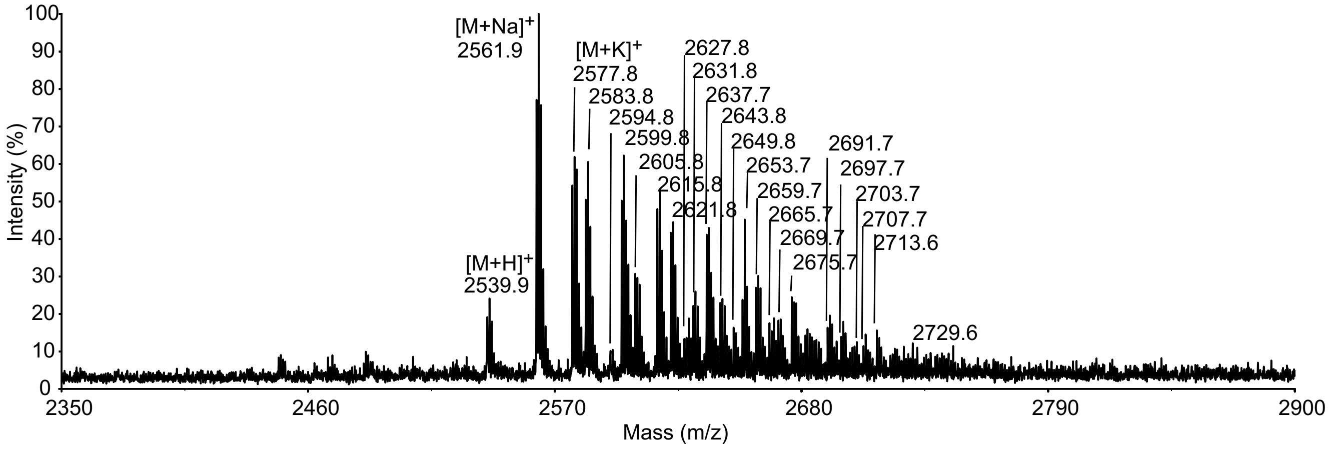

GalNAc-Tetra-SDC2

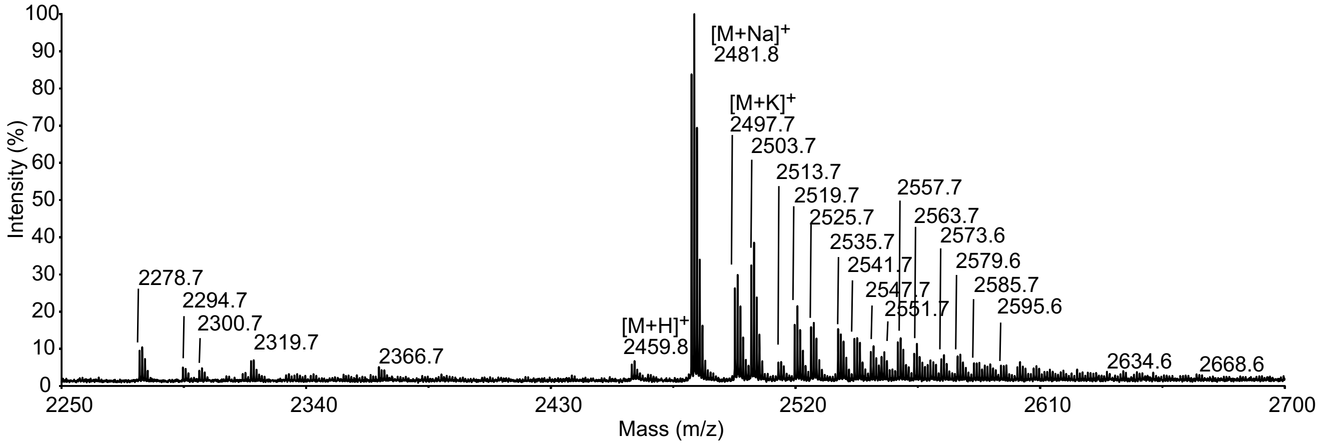

GalNAc-TetraP-SDC2

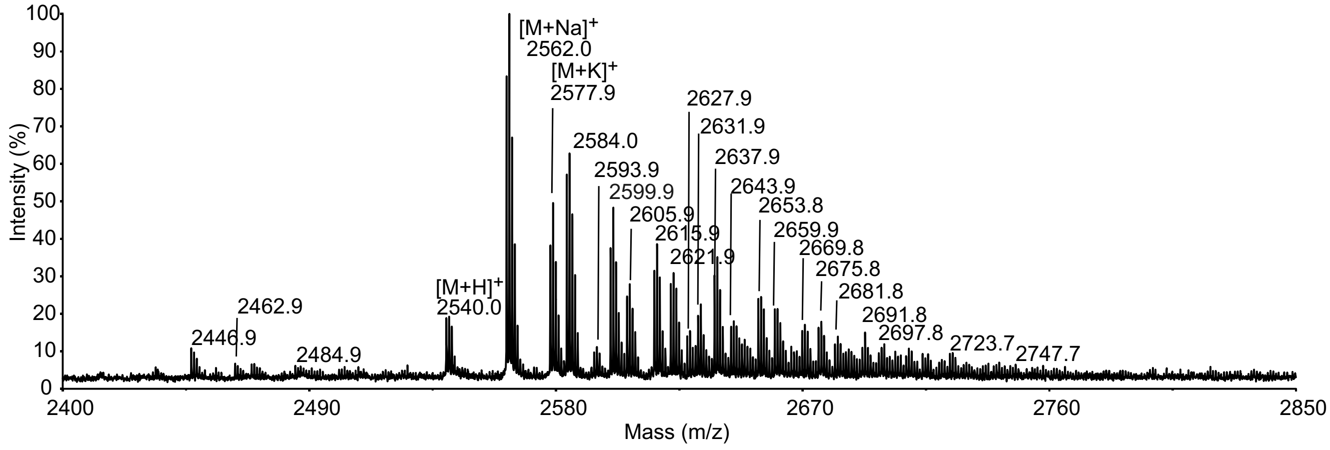

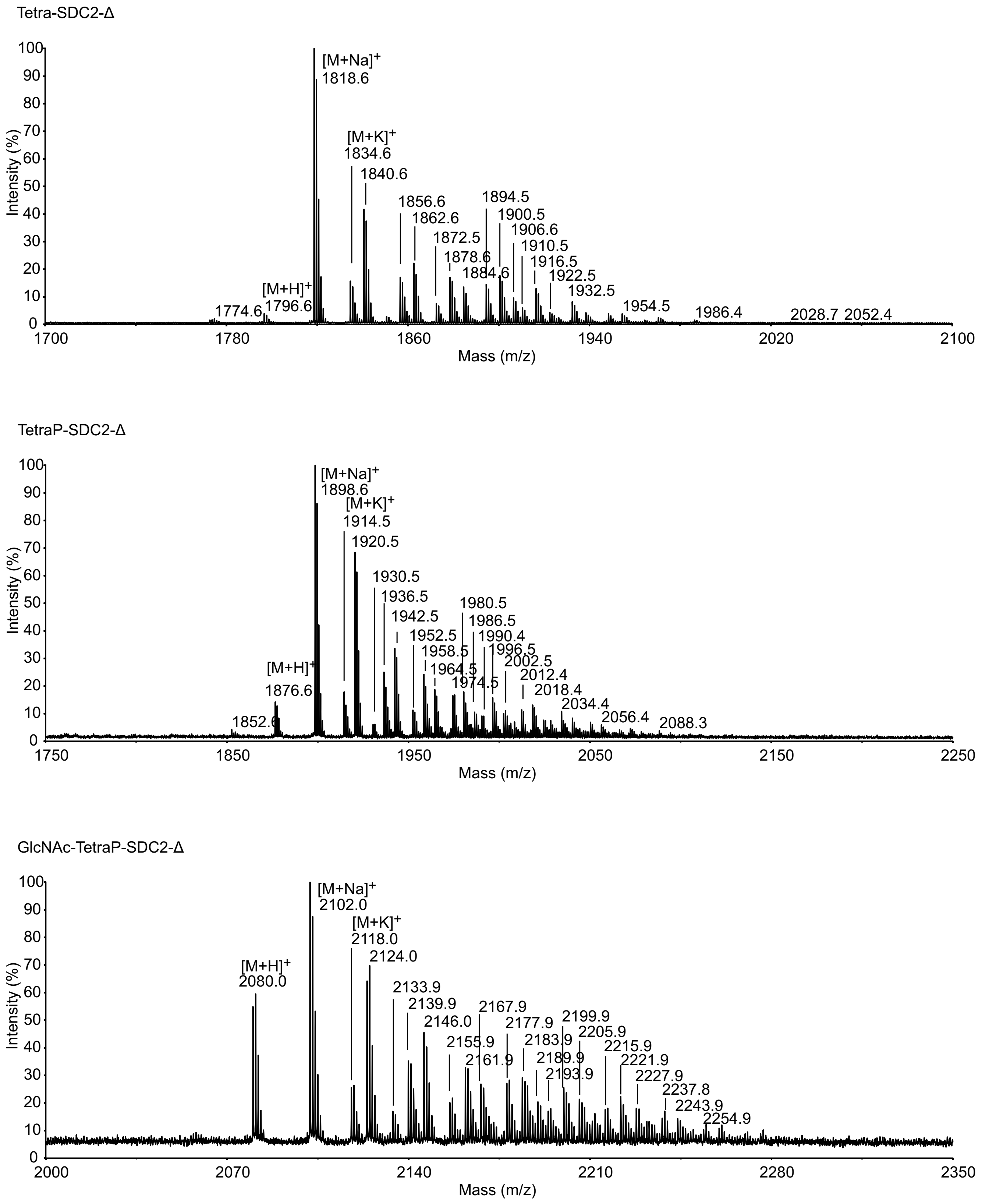
Tetra-SDC2-Δ
[M+Na]+ 1818.6
[M+K]+ 1834.6
[M+H]+ 1796.6
1774.6
1840.6
1856.6
1862.6
1872.5
1878.6
1884.6
1894.5
1900.5
1906.6
1910.5
1916.5
1922.5
1932.5
1954.5
1986.4
2028.7
2052.4
Intensity (%)
Mass (m/z)
TetraP-SDC2-Δ
[M+Na]+ 1898.6
[M+K]+ 1914.5
[M+H]+ 1876.6
1852.6
1920.5
1930.5
1936.5
1942.5
1952.5
1958.5
1964.5
1974.5
1980.5
1986.5
1990.4
1996.5
2002.5
2012.4
2018.4
2034.4
2056.4
2088.3
Intensity (%)
Mass (m/z)
GlcNAc-TetraP-SDC2-Δ
[M+Na]+ 2102.0
[M+K]+ 2118.0
[M+H]+ 2080.0
2124.0
2133.9
2139.9
2146.0
2155.9
2161.9
2167.9
2177.9
2183.9
2189.9
2193.9
2199.9
2205.9
2215.9
2221.9
2227.9
2237.8
2243.9
2254.9
Intensity (%)
Mass (m/z)

GalNAc-Tetra-SDC2-Δ

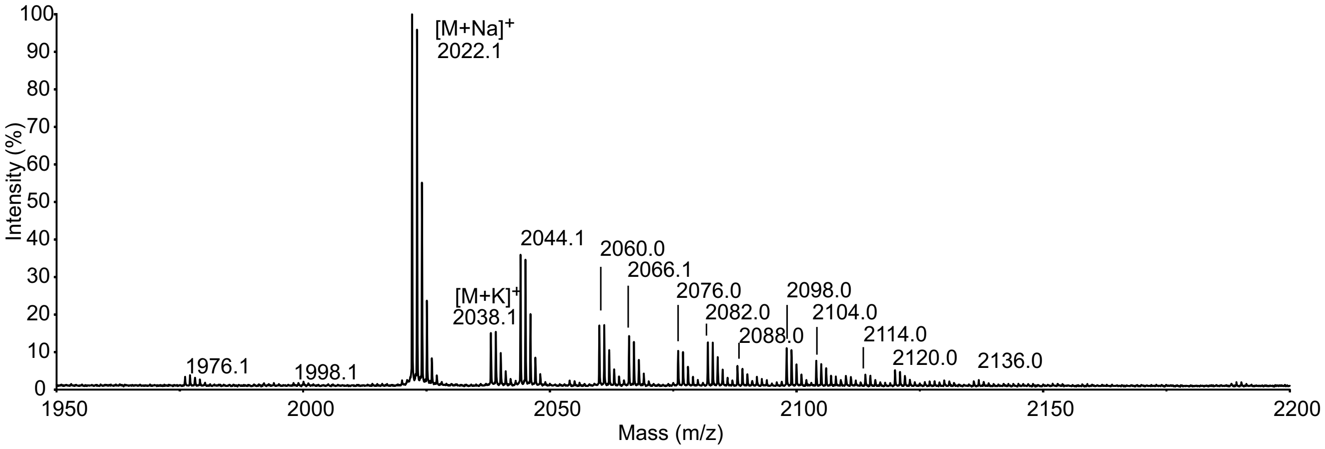

GalNAc-TetraP-SDC2-Δ

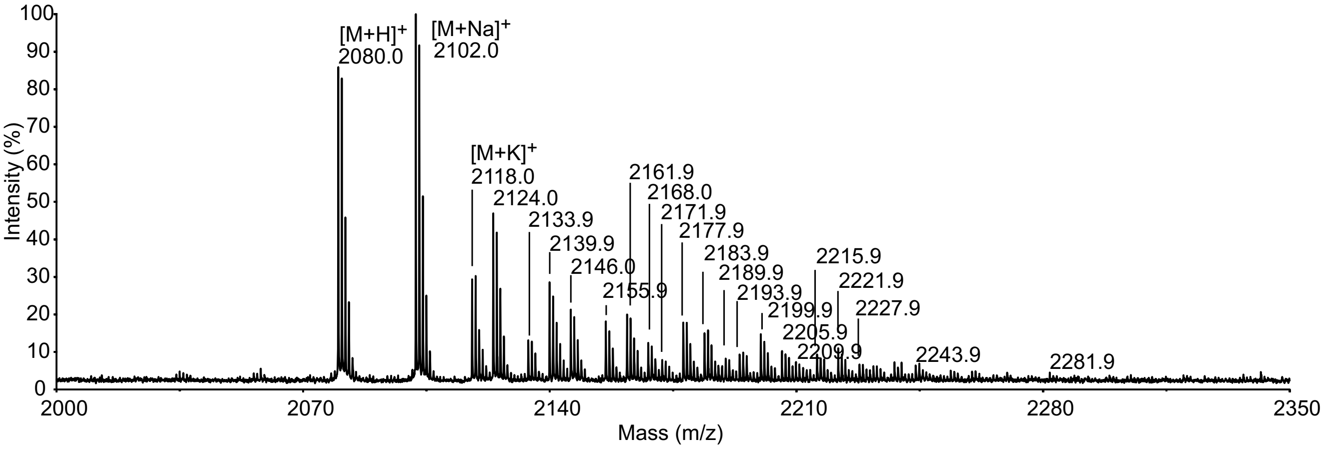

$Tetra_2$-SDC4

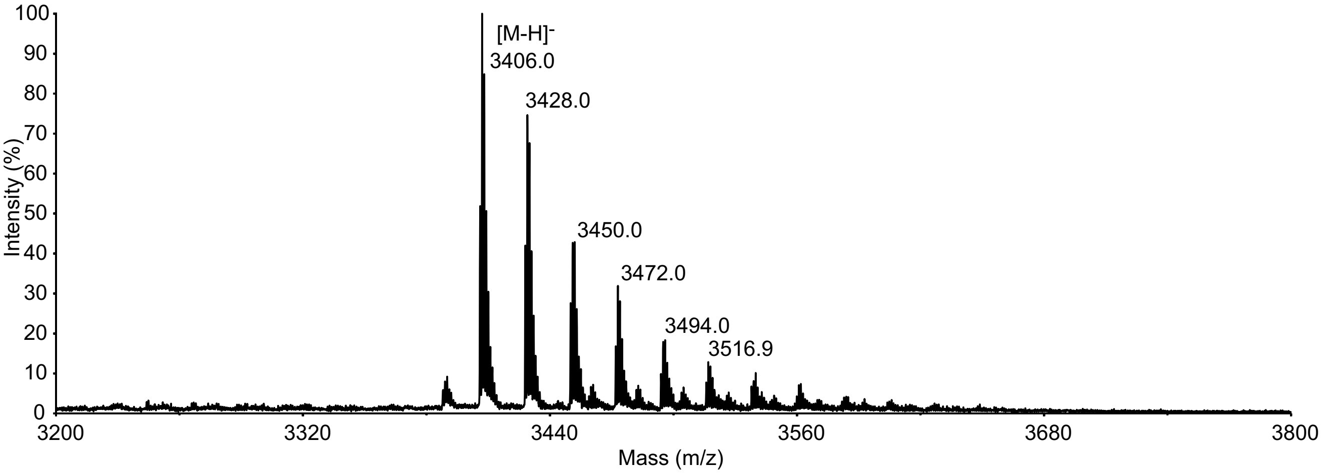

TetraP$_2$-SDC4

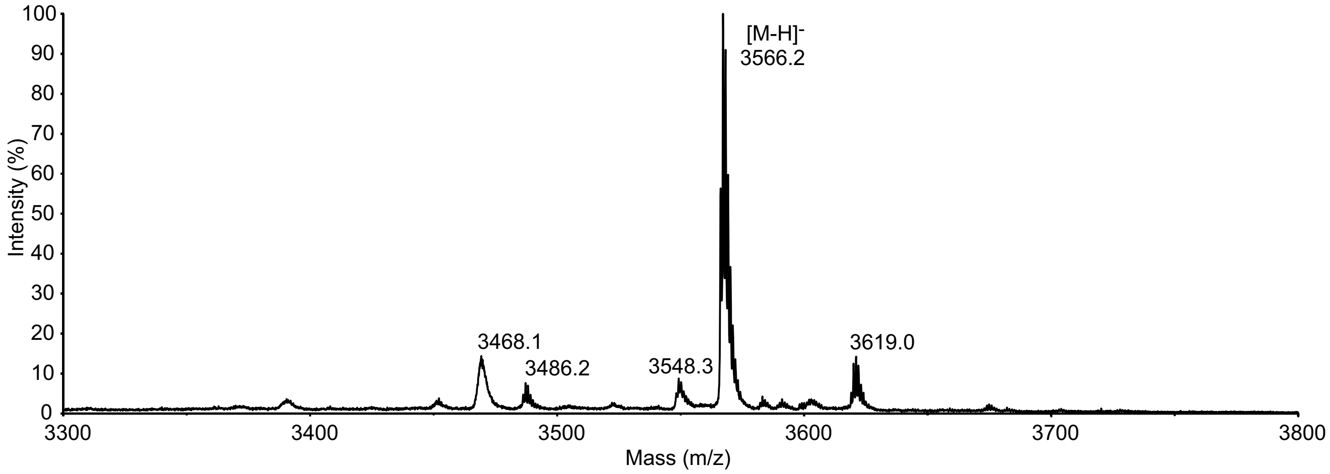

GlcNAc-Tetra$_2$-SDC4

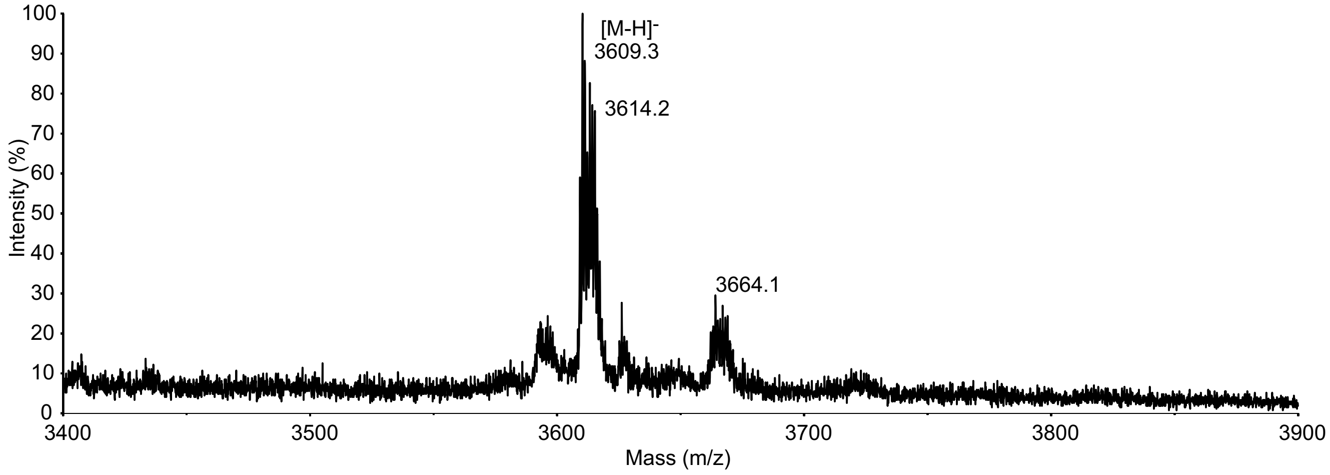

GlcNAc$_2$-Tetra$_2$-SDC4

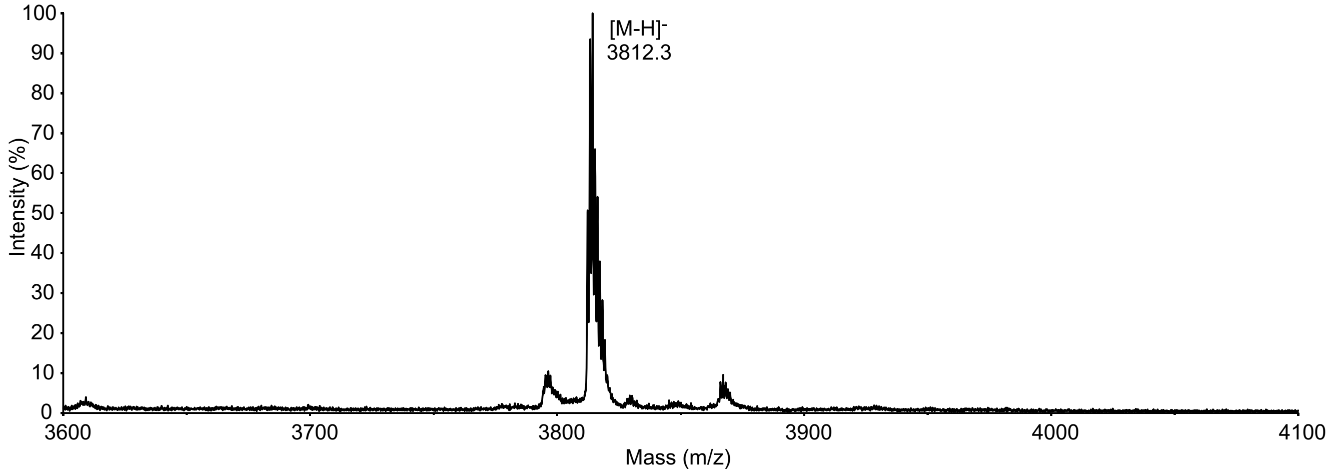

GalNAc-Tetra$_2$-SDC4

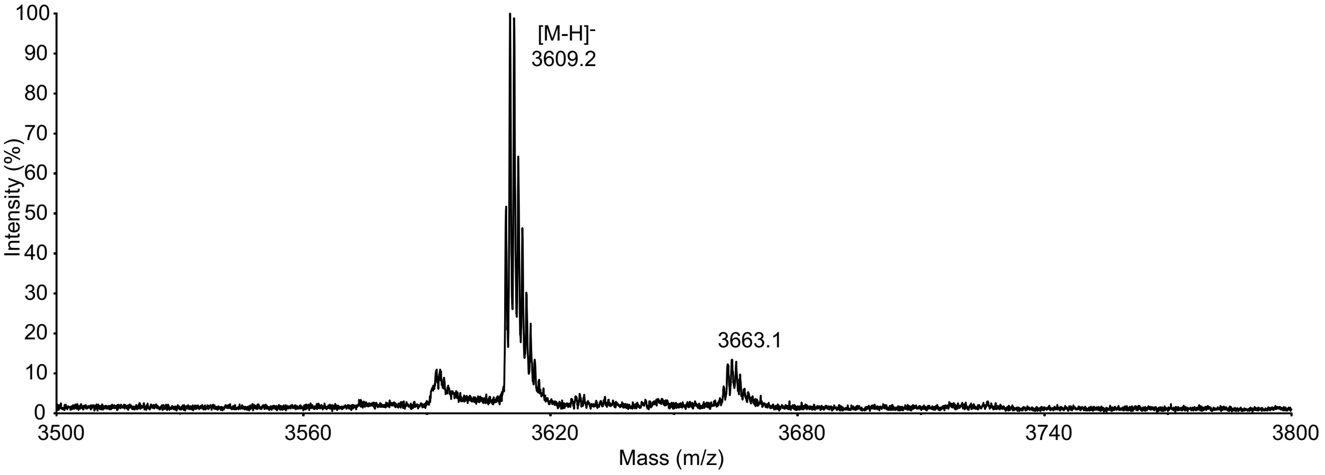

GalNAc$_2$-Tetra$_2$-SDC4

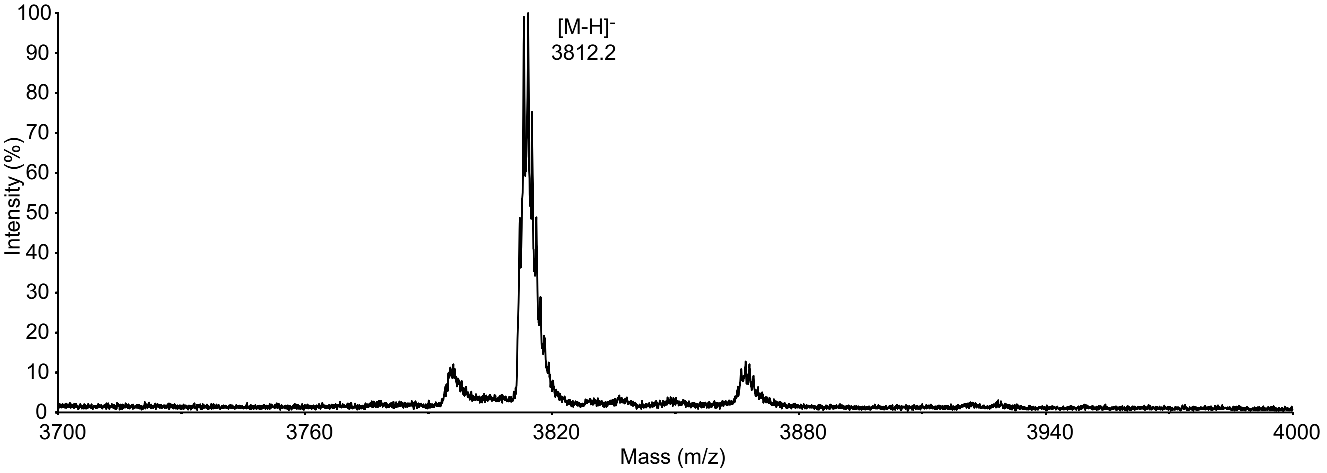

Tetra-SCD4-A

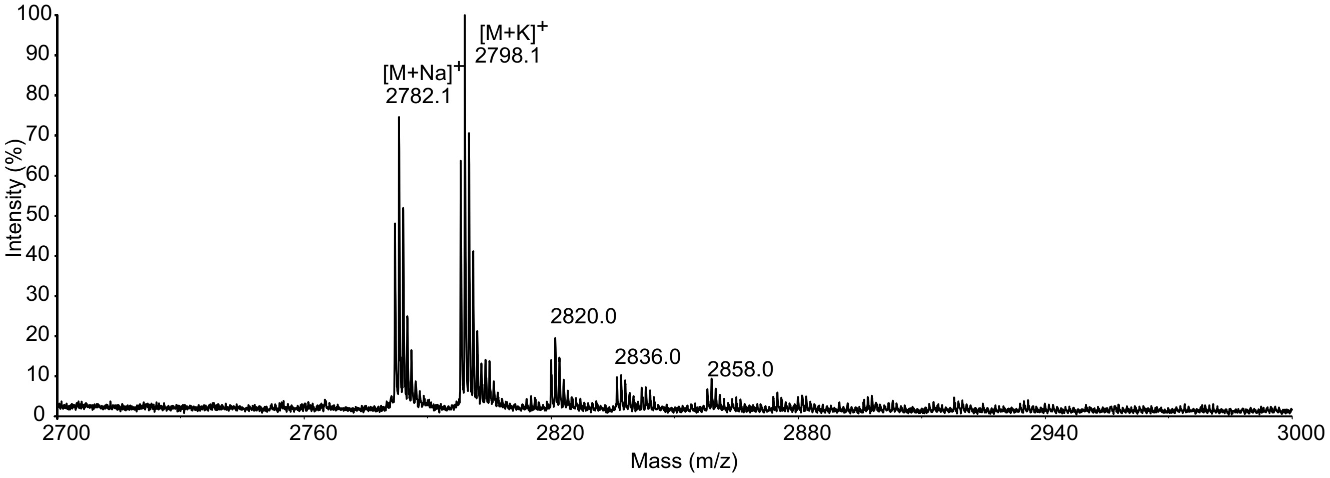

TetraP-SCD4-A

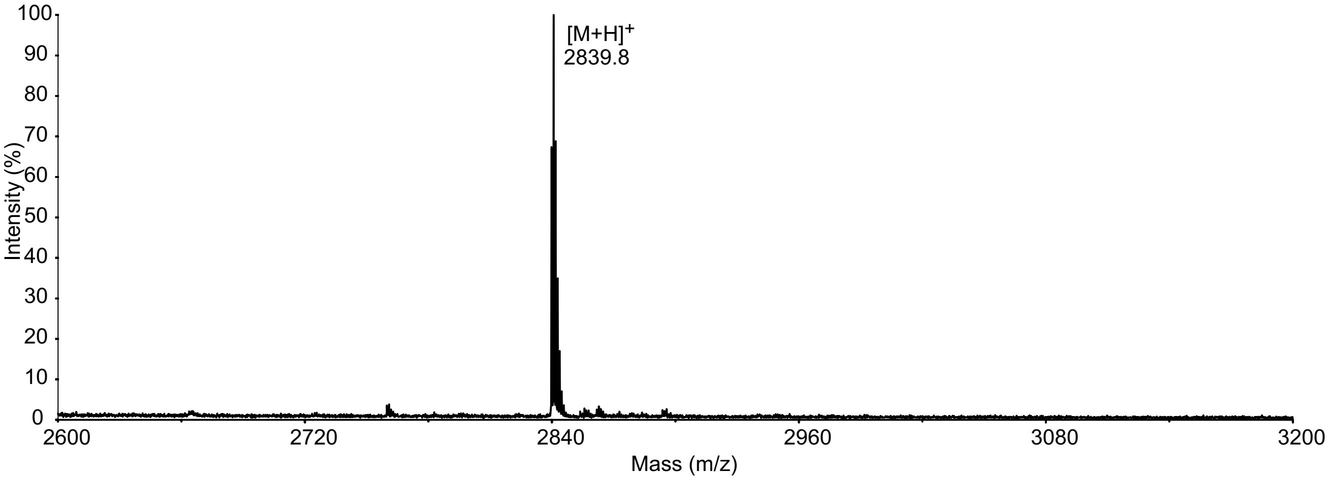

Xyl_3-GPC1

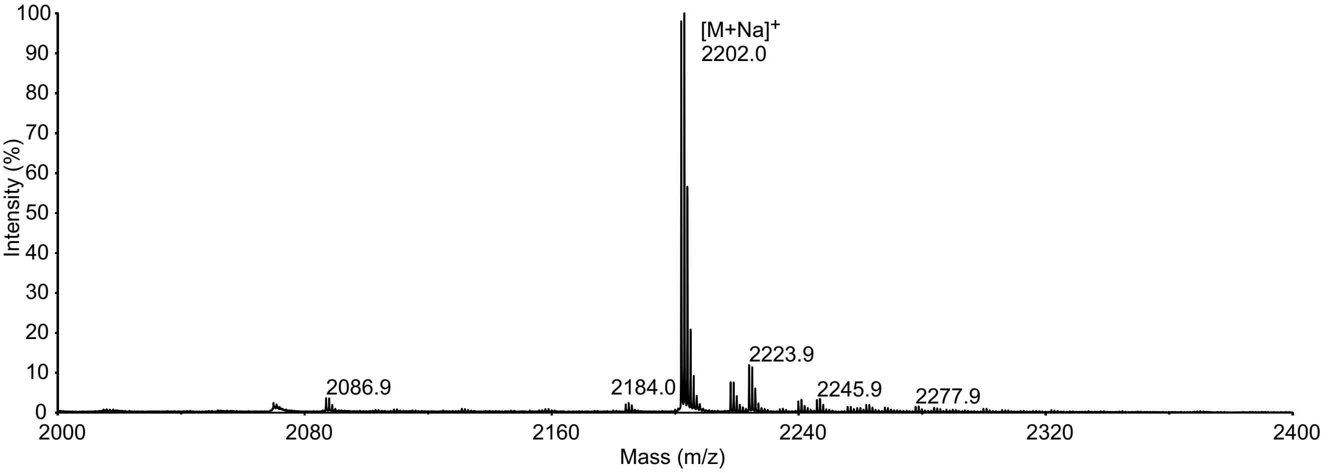

$(Gal\text{-}Xyl)_3$-GPC1

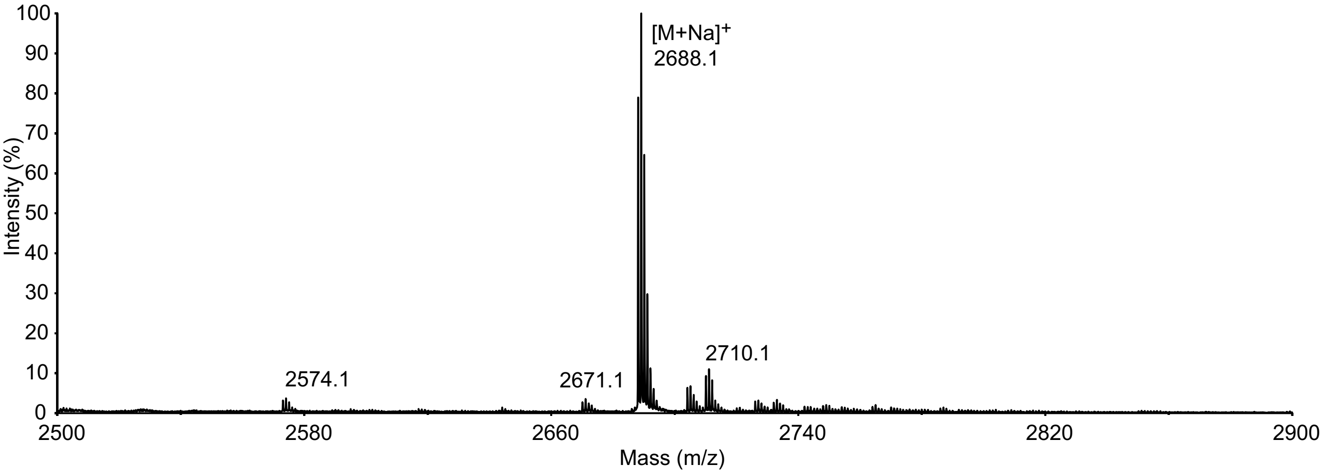

(Gal-Gal-Xyl)$_3$-GPC1

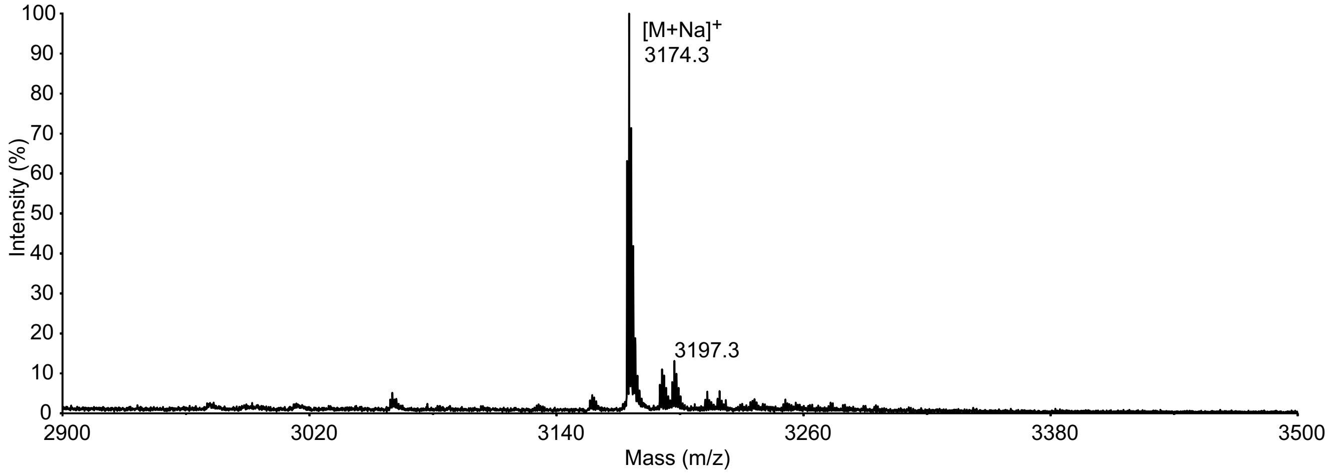

Tetra$_3$-GPC1

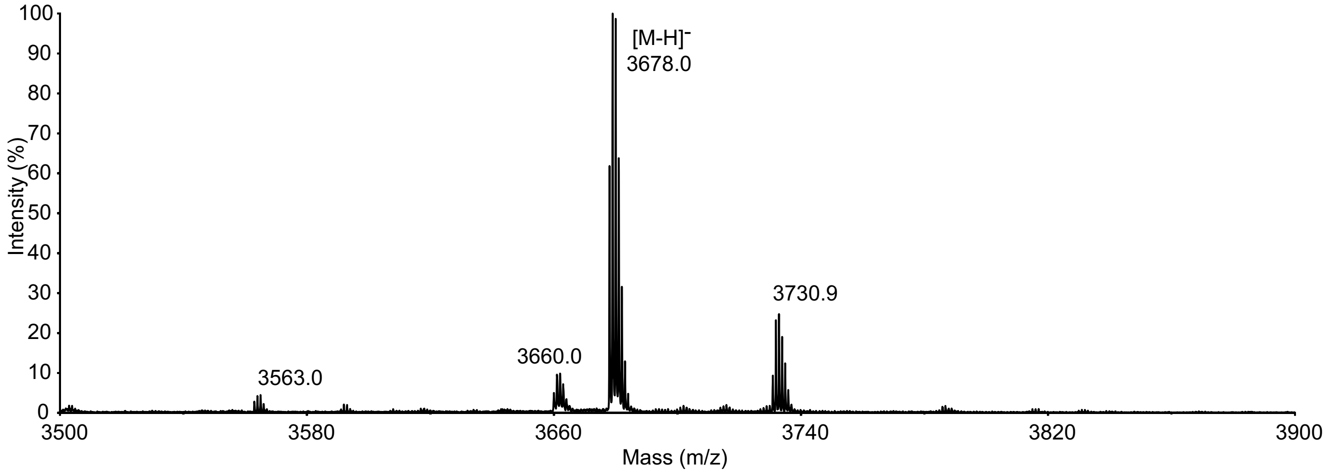

TetraP$_3$-GPC1

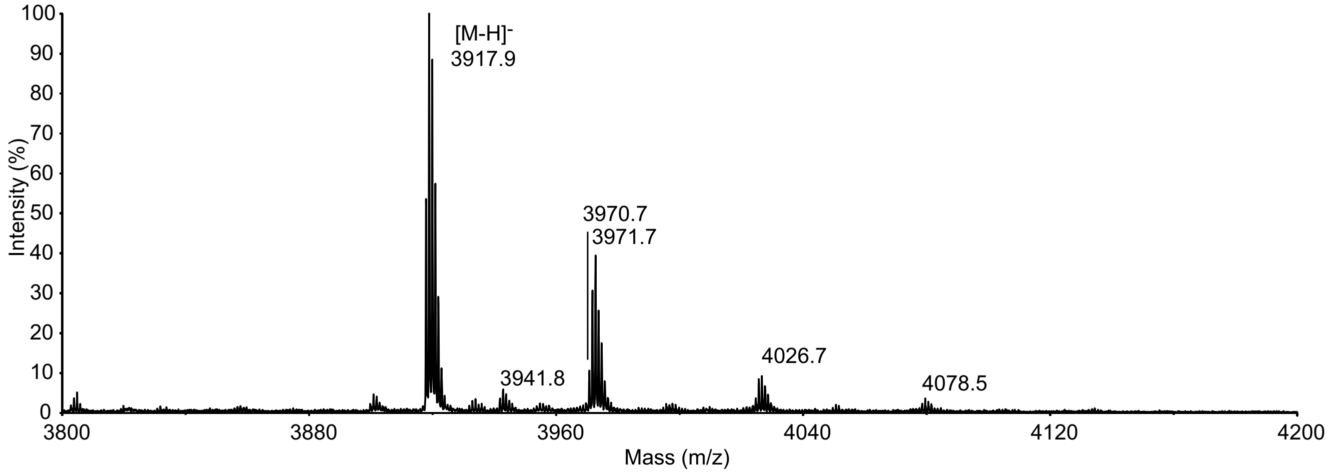

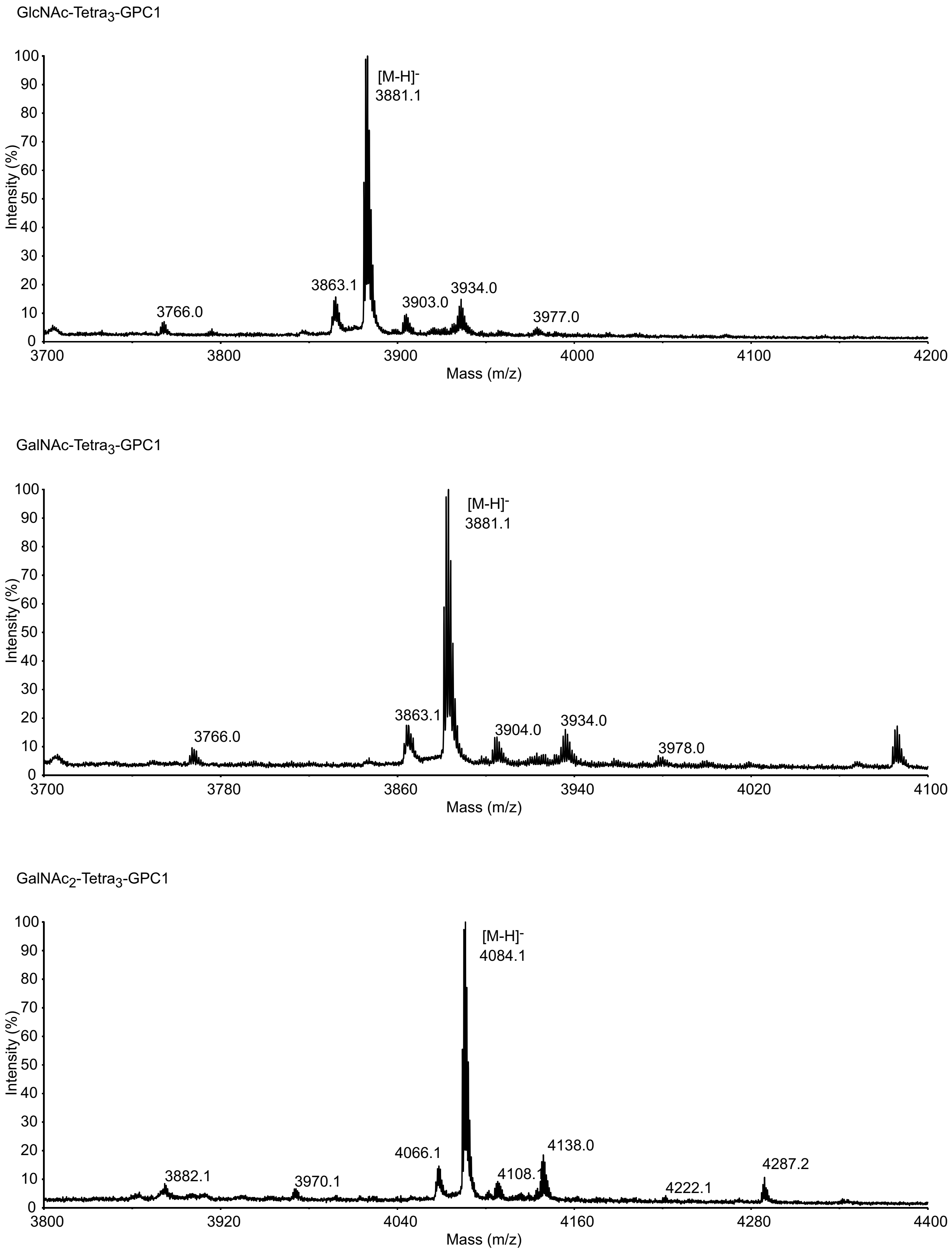
GlcNAc-Tetra$_3$-GPC1
Intensity (%)
Mass (m/z)
[M-H]$^-$
3881.1
3766.0
3863.1
3903.0
3934.0
3977.0
GalNAc-Tetra$_3$-GPC1
Intensity (%)
Mass (m/z)
[M-H]$^-$
3881.1
3766.0
3863.1
3904.0
3934.0
3978.0
GalNAc$_2$-Tetra$_3$-GPC1
Intensity (%)
Mass (m/z)
[M-H]$^-$
4084.1
3882.1
3970.1
4066.1
4108.1
4138.0
4222.1
4287.2

GalNAc$_3$-Tetra$_3$-GPC1

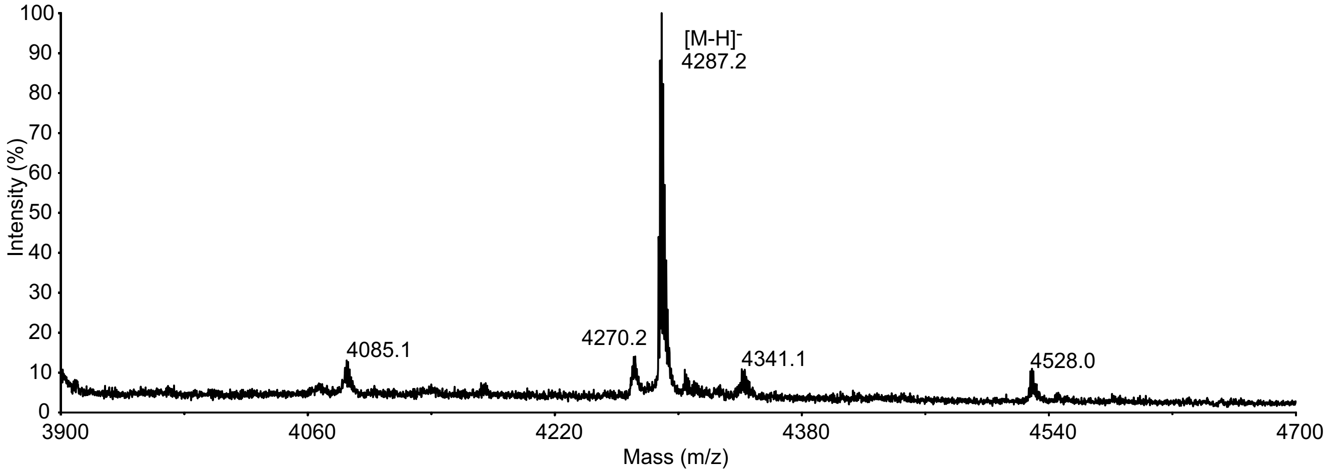

Supplement: Supplementary file 5 — Supplementary Dataset 1 [file 41467_2023_42236_MOESM5_ESM.pdf]
