# Supplementary figures and images for: Molecular mechanism of decision-making in glycosaminoglycan biosynthesis
Source: Nat Commun. 2023 Oct 13;14:6425. doi: 10.1038/s41467-023-42236-z (PMC10570366; doi:10.1038/s41467-023-42236-z)

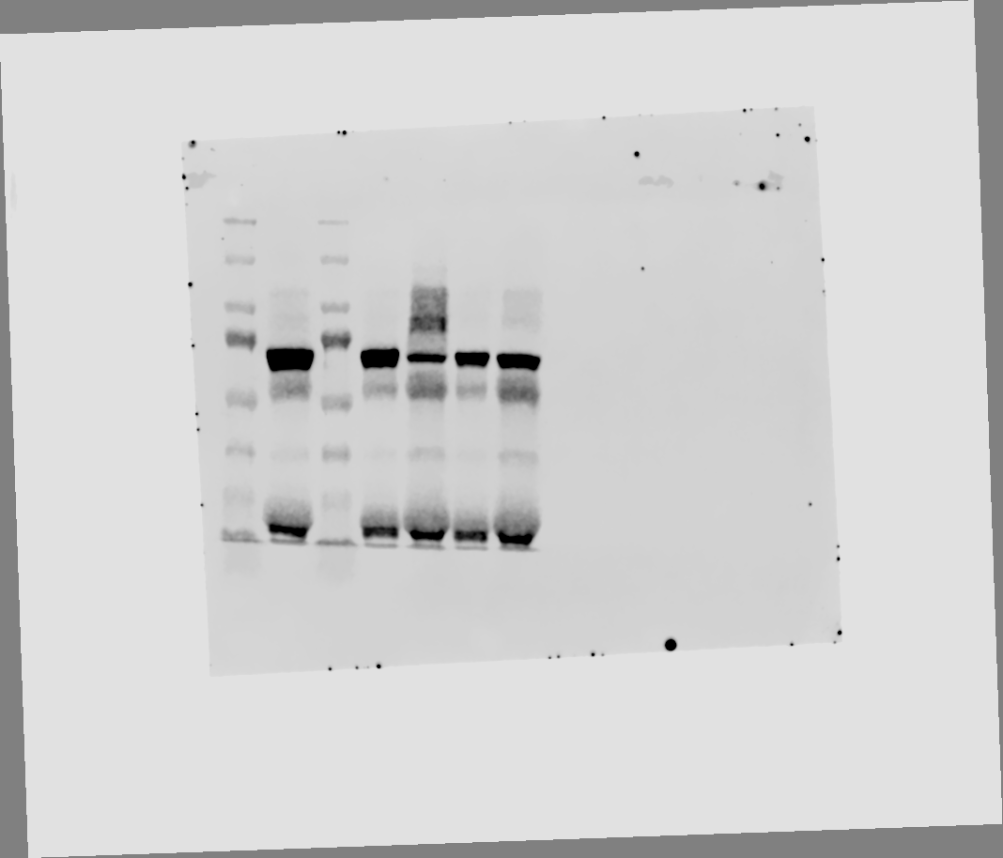

Supplement: Supplementary file 6 — Source Data [file 41467_2023_42236_MOESM6_ESM.zip › Source_Data/Supplementary_Figure_10a_uncropped.tif]

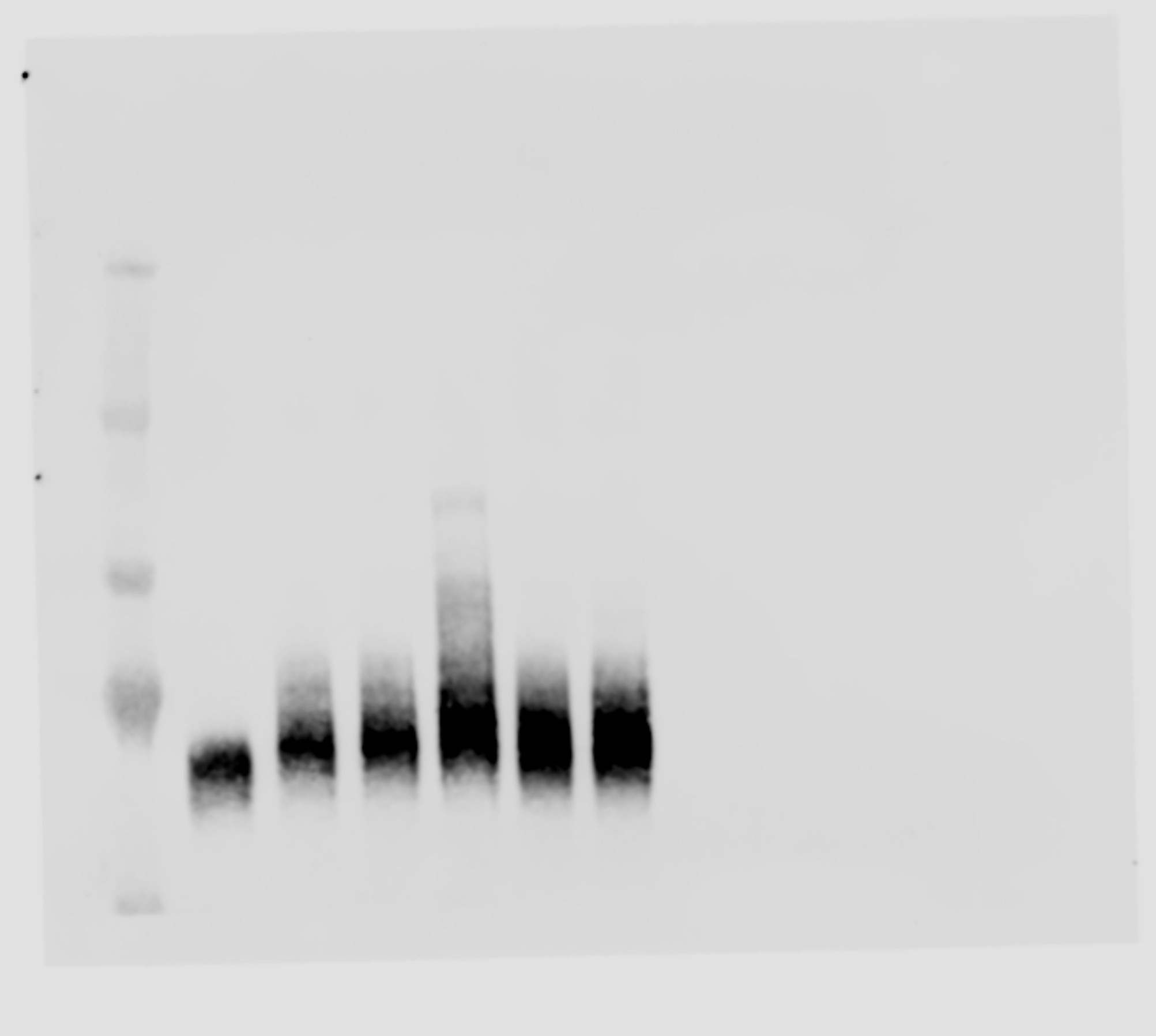

Supplement: Supplementary file 6 — Source Data [file 41467_2023_42236_MOESM6_ESM.zip › Source_Data/Figure_5a_uncropped.tif]

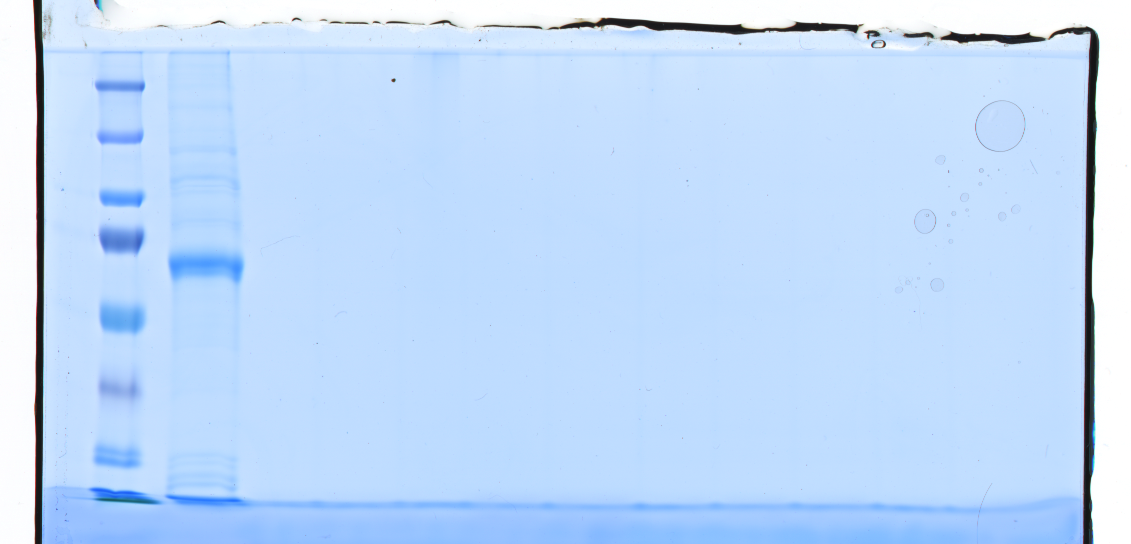

Supplement: Supplementary file 6 — Source Data [file 41467_2023_42236_MOESM6_ESM.zip › Source_Data/Supplementary_Figure_10b_uncropped.tif]

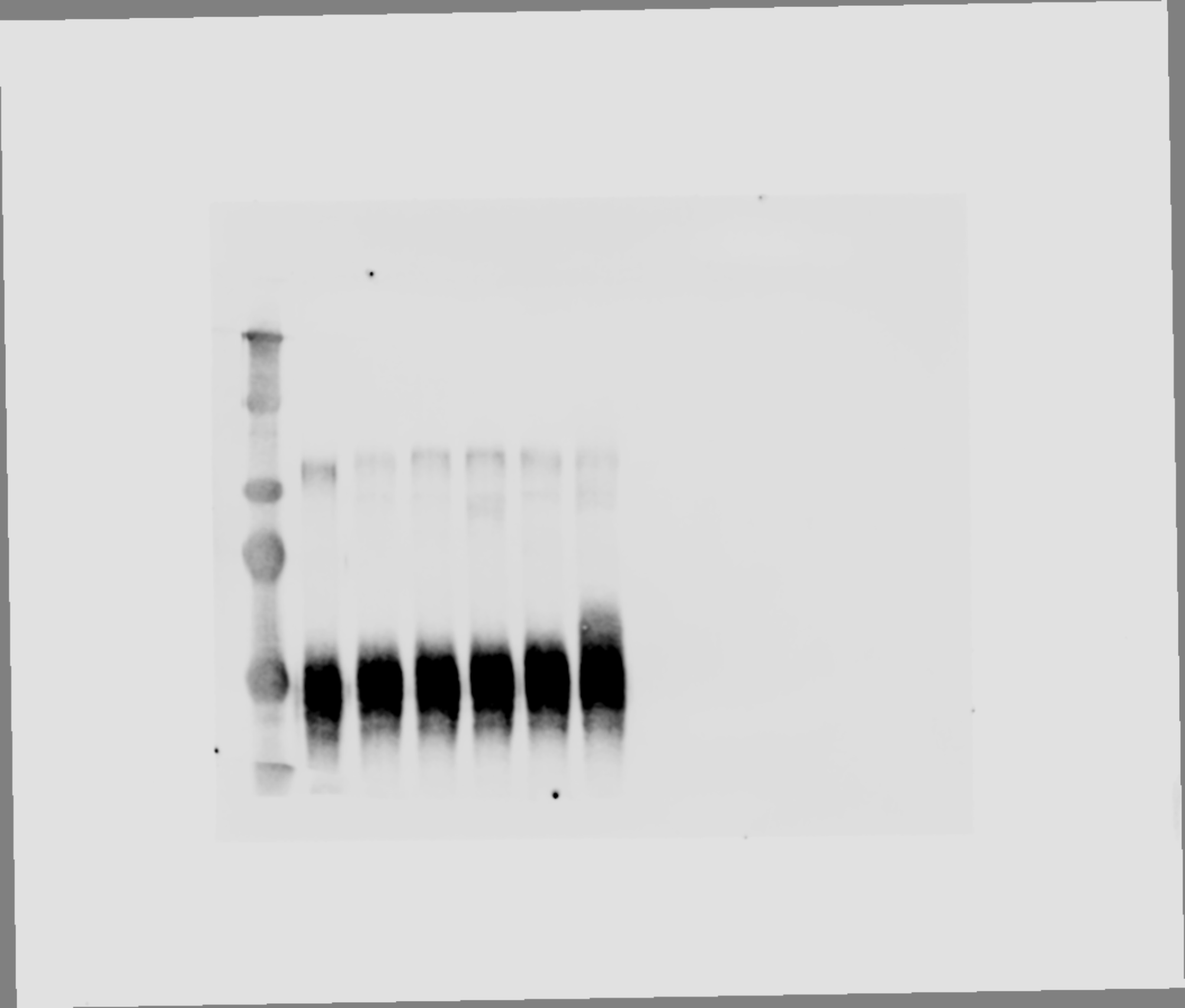

Supplement: Supplementary file 6 — Source Data [file 41467_2023_42236_MOESM6_ESM.zip › Source_Data/Figure_5b_uncropped.tif]

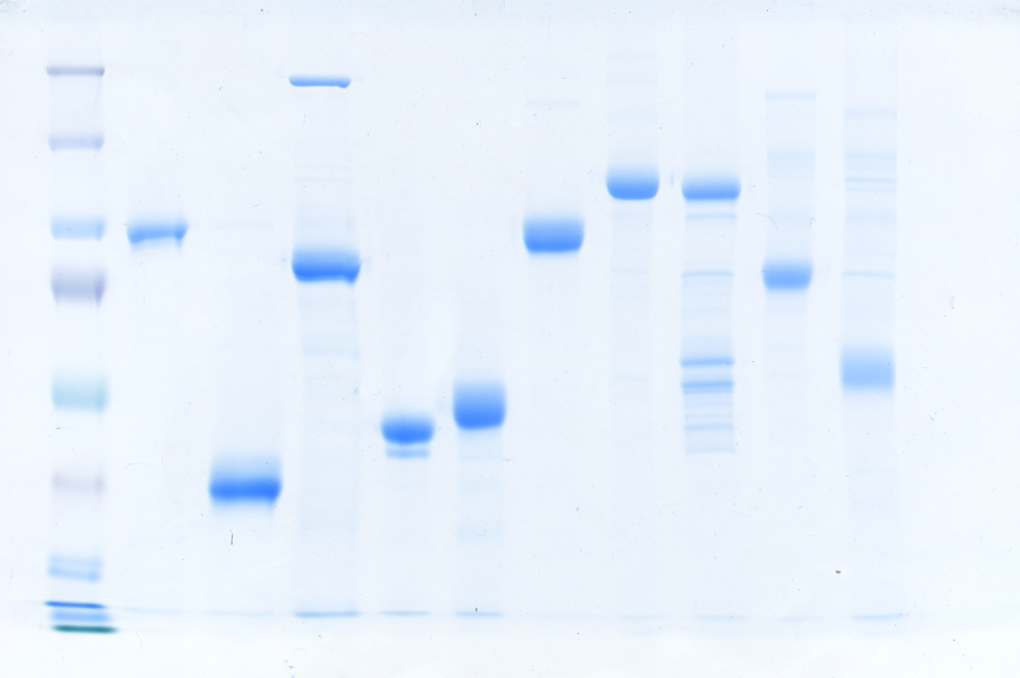

Supplement: Supplementary file 6 — Source Data [file 41467_2023_42236_MOESM6_ESM.zip › Source_Data/Supplementary_Figure_1a_uncropped.tif]

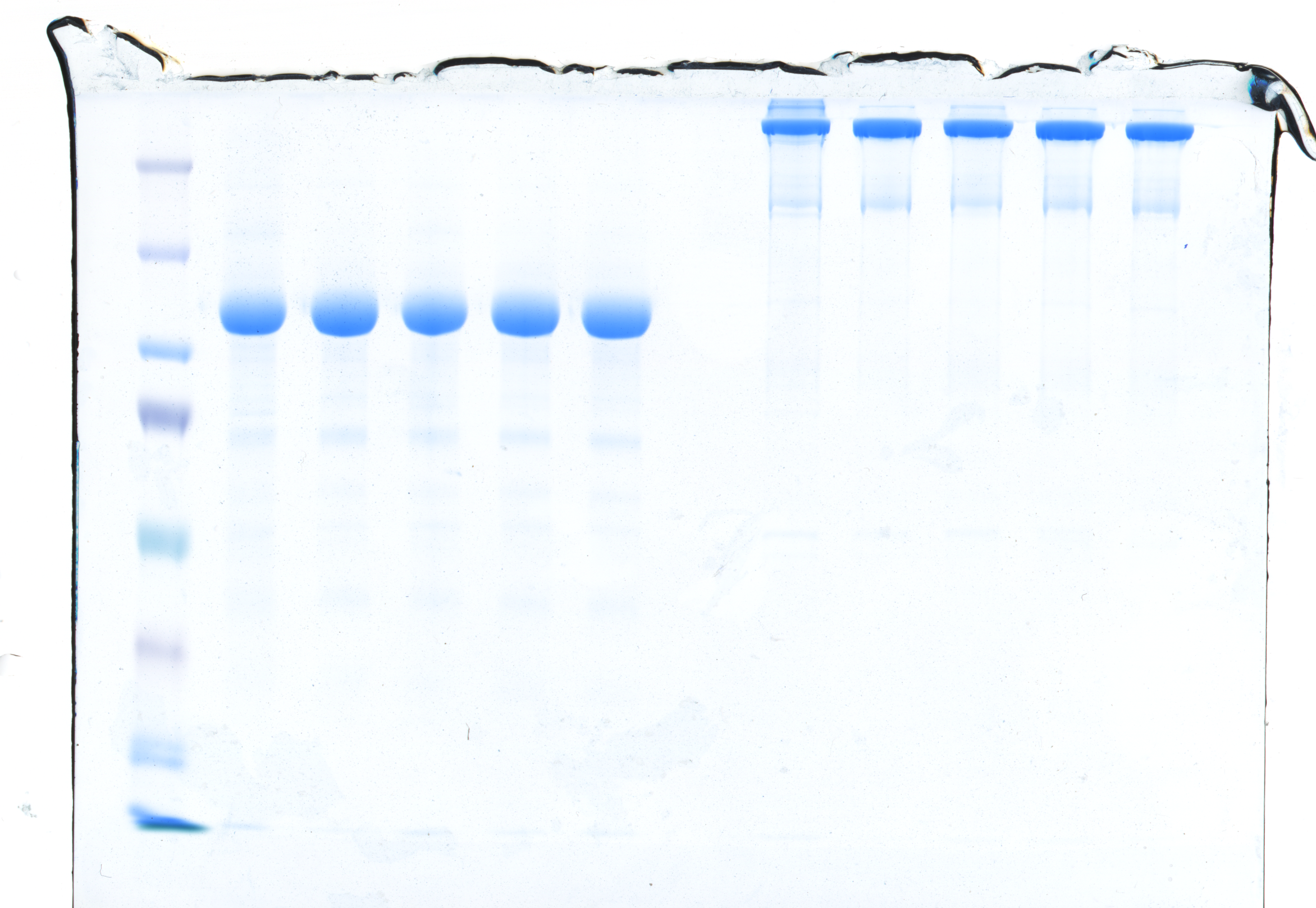

Supplement: Supplementary file 6 — Source Data [file 41467_2023_42236_MOESM6_ESM.zip › Source_Data/Supplementary_Figure_7b_uncropped.tif]

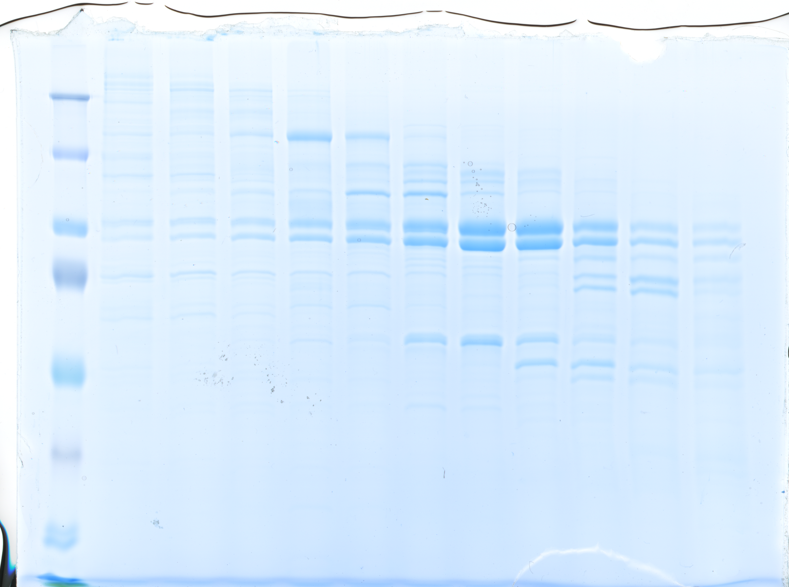

Supplement: Supplementary file 6 — Source Data [file 41467_2023_42236_MOESM6_ESM.zip › Source_Data/Supplementary_Figure_1b_uncropped.tif]

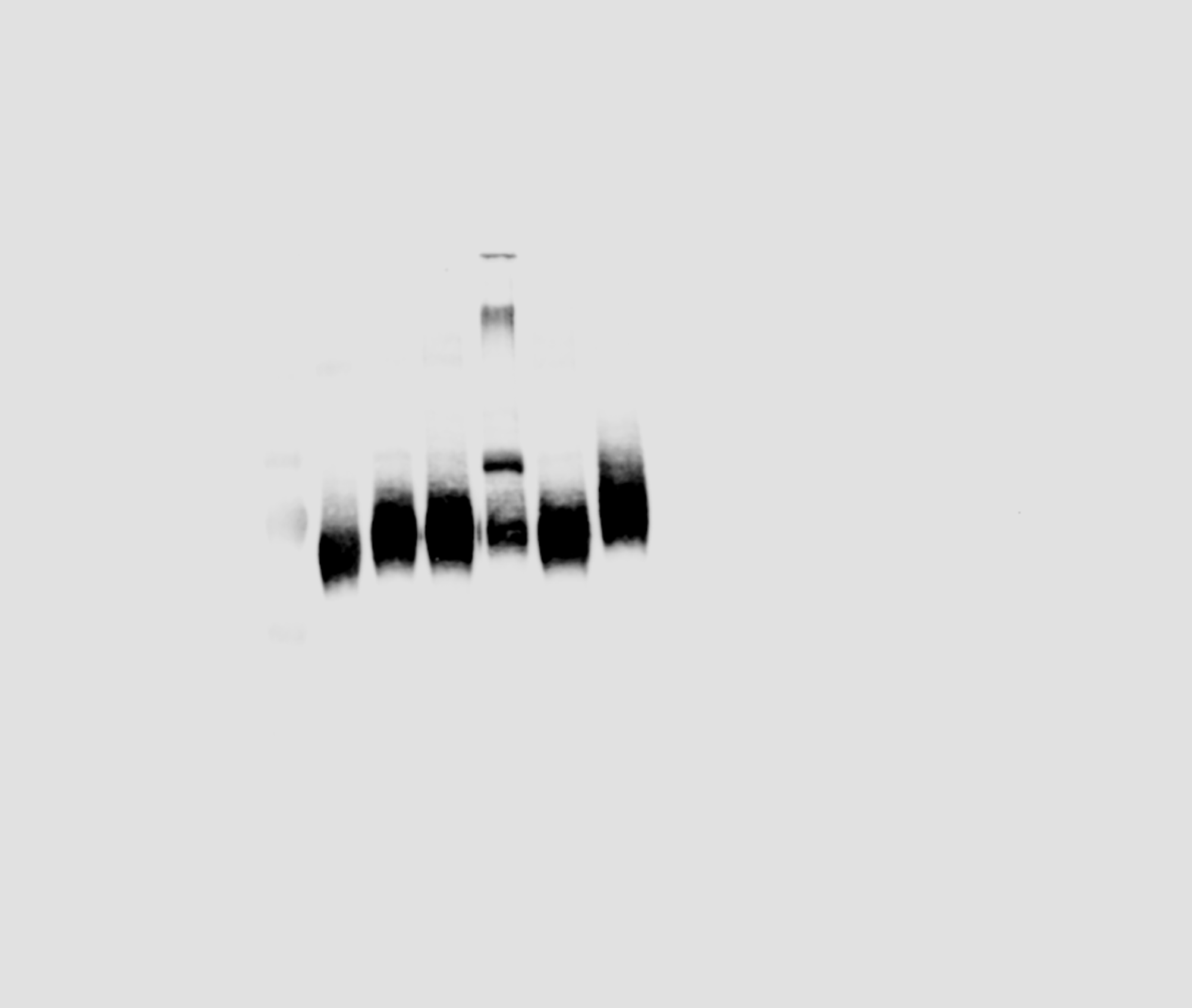

Supplement: Supplementary file 6 — Source Data [file 41467_2023_42236_MOESM6_ESM.zip › Source_Data/Supplementary_Figure_10c_uncropped.tif]

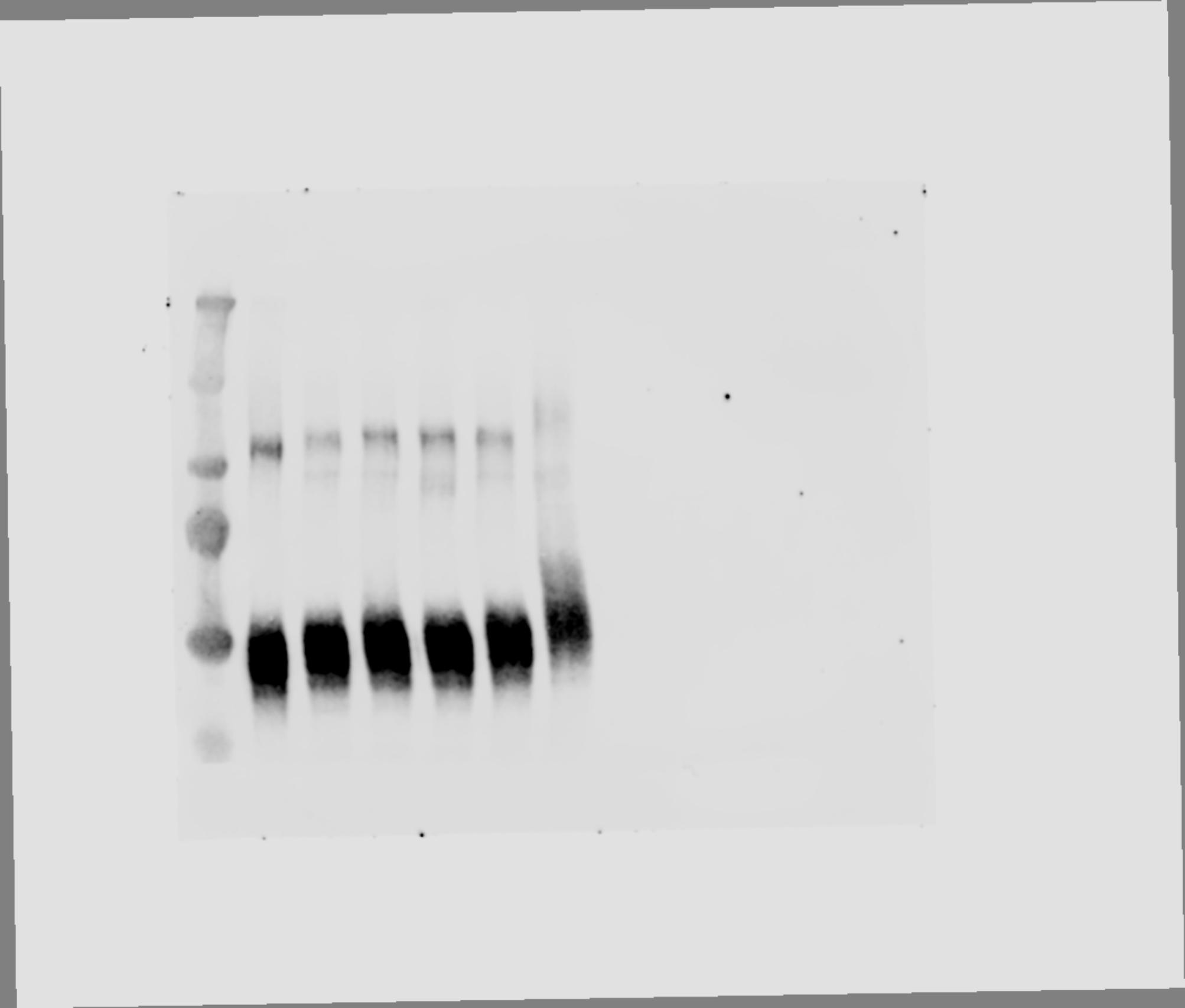

Supplement: Supplementary file 6 — Source Data [file 41467_2023_42236_MOESM6_ESM.zip › Source_Data/Supplementary_Figure_10d_uncropped.tif]
